# Atg18 interaction positions Atg2 for efficient lipid transfer into phagophore elongation

**DOI:** 10.1038/s44318-026-00802-3

**Published:** 2026-05-20

**Authors:** Sabrina Chumpen Ramirez, Dmitry Shvarev, Prado Vargas Duarte, Yara Ahmed, Jana Milach, Emma Lang, Stefan Kuchenbuch, Stefano Vanni, Fulvio Reggiori, Arne Moeller, Christian Ungermann

**Affiliations:** 1 Osnabrück University, Department of Biology/Chemistry, Biochemistry Section, Osnabrück, Germany; 2 Osnabrück University, Center of Cellular Nanoanalytics, Osnabrück, Germany; 3 Osnabrück University, Department of Biology/Chemistry, Structural Biology Section, Osnabrück, Germany; 4 Department of Biomedicine, Aarhus University, Aarhus C, Denmark; 5 Department of Biology, Fribourg, Switzerland; 6 Swiss National Center for Competence in Research Bio-inspired Materials, University of Fribourg, Fribourg, Switzerland

**Keywords:** Autophagy & Cell Death, Membranes & Trafficking

## Abstract

During macroautophagy, the de novo formation of the autophagosome at a membrane contact site (MCS) with the endoplasmic reticulum requires directional lipid flux for the growth of the initial phagophore before its sealing into an autophagosome and subsequent fusion with the lysosome/vacuole. It remains unclear, however, how the formation of this specialized MCS and the directionality of the lipid flux are controlled. Here, we present the structure of the key lipid transfer protein Atg2 from yeast solved together with its Atg18 binding partner, a phosphatidylinositol-3-phosphate (PtdIns3P) effector, using cryo-electron microscopy. We reveal a new interface in Atg2 that, together with PtdIns3P, is required for Atg18 recruitment and lipid transfer activity. Furthermore, we visualize lipid densities along the internal hydrophobic cavity of Atg2, providing structural evidence that Atg2 cavity is filled with lipids throughout the entire length, even when Atg2 is cytosolic. Finally, molecular dynamics simulations show that the complex generates membrane curvature, efficiently positioning the lipid channel of Atg2 towards the membrane to promote lipid transfer into the elongating phagophore.

## Synopsis

During the early steps of autophagy, Atg2-Atg18 protein pairs feed membrane lipids into the growing autophagosome through a hydrophobic channel. The molecular architecture presented in this article explains how PtdIns3P-dependent membrane binding positions this channel for efficient and directional lipid transfer.

- The structure of the Atg2-Atg18 complex from yeast is solved by cryo-EM.
- Cryo-EM analysis of Atg2-Atg18 revealed lipid densities in the hydrophobic cavity of Atg2.
- The amino acid residues 922-934 in Atg2 constitute an interaction interface with Atg18 that is essential for autophagy.
- The Atg2-Atg18 complex has the capability to curve membranes.
- Molecular dynamics simulations reveal Atg2 channel positioning and lipid transfer towards the phagophore membrane.

## Abstract (web summary)

Atg18 is a positioning factor that aligns the Atg2 lipid-transfer channel at the membrane during the early steps of autophagy.

## Graphical abstract

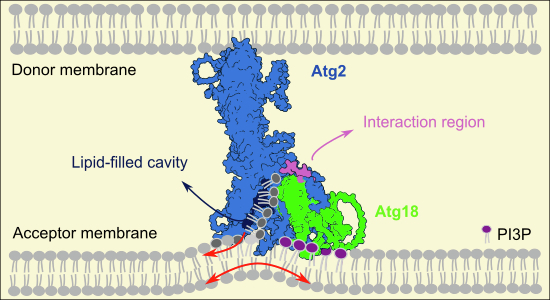

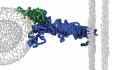

## Introduction

Macroautophagy, hereafter autophagy, is a conserved catabolic pathway mediated by a group of ~20 conserved autophagy-related (Atg) proteins (Nakatogawa, 2020; Vargas et al, 2023). These proteins are recruited to the phagophore assembly site (PAS) individually or as separate complexes, and coordinate the formation of the phagophore, the precursor of the autophagosome. Autophagy is for example regulated by the target of rapamycin complex 1 (TORC1), which phosphorylates Atg13 as part of the regulatory Atg1 master kinase. Upon nutrient starvation, TORC1 is inactivated, resulting in dephosphorylation of Atg13 and auto-activation of the kinase Atg1. Activated Atg1 triggers the recruitment and activation of downstream Atg proteins, including Atg9, the only transmembrane protein of the core Atg machinery. Atg9-positive vesicles are recruited to the PAS together with endoplasmic reticulum (ER)-derived COPII-vesicles, and they presumably fuse to form the phagophore as a seed structure for autophagosome biogenesis (Sawa-Makarska et al, 2020; Olivas et al, 2023). This early structure elongates through lipid transfer from the ER at a phagophore-ER MCS (Gómez-Sánchez et al, 2018; Schütter et al, 2020; Hao et al, 2025), and possibly at MCSs with other organelles (Bieber et al, 2022).

Lipid transfer toward the phagophore mainly depends on the bridge-like lipid transfer protein (BLTP) Atg2 and its binding partner Atg18 (WIPI4 in mammals), a β-propeller protein that binds PtdIns3P and PtdIns(3,5)P_2_ (Baskaran et al, 2012; Krick et al, 2012; Watanabe et al, 2012). Single copies of both proteins form a complex with Atg9 trimers (Vliet et al, 2022; Wang et al, 2025), which scrambles the lipids delivered by Atg2 to the phagophore (Chowdhury et al, 2018; Kotani et al, 2018). The Atg9-Atg2 interaction is essential for phagophore elongation in vivo (Gómez-Sánchez et al, 2018). In addition, Atg9 stimulates Atg2-mediated lipid transfer in vitro (Nguyen et al, 2023; Chumpen Ramirez et al, 2023), by either alleviating membrane tension and/or regulating Atg2 function. Furthermore, efficient lipid transfer requires membranes of high curvature containing negatively charged lipids, and local synthesis of PtdIns3P (Maeda et al, 2019; Osawa et al, 2019; Valverde et al, 2019; Gómez-Sánchez et al, 2025). Atg2/ATG2 proteins have been described as rod-shaped proteins capable of binding lipids (Valverde et al, 2019). X-ray crystallography of their N-terminal region revealed acyl-chain densities corresponding to phospholipids within a hydrophobic cavity (Osawa et al, 2019). These findings led to the current model proposing that the lipid transfer occurs through a bridge-like mechanism, in which a long hydrophobic groove within Atg2/ATG2 proteins shuttles lipids between the ER and the elongating phagophore. Importantly, this activity is strongly stimulated by the interaction of Atg2 with Atg18 (Maeda et al, 2019; Osawa et al, 2020), an observation that led to the hypothesis that Atg18 may mediate Atg2 recruitment to the PtdIns3P-positive PAS (Obara et al, 2008; Chowdhury et al, 2018; Kotani et al, 2018). However, Atg2 can bind and tether membranes independently from Atg18, and its localization at the phagophore is necessary for the recruitment of Atg18, and not vice versa (Rieter et al, 2013; Gómez-Sánchez et al, 2018, 2025). Thus, the molecular mechanism behind the Atg18-mediated stimulation of the lipid transfer by Atg2 remains unclear. To gain insight into this process, we solved the structure of the *Saccharomyces cerevisiae* Atg2-Atg18 complex by cryo-electron microscopy (cryo-EM) and identified an interface that is critical for both Atg2-mediated recruitment of Atg18 to the PAS and Atg18-mediated stimulation of Atg2. Importantly, our analyses confirmed the presence of lipids throughout the internal hydrophobic cavity of Atg2 providing structural evidence of its role as lipid-conducting channel during phagophore elongation. Finally, we investigated membrane binding and lipid transfer activity of the Atg2-Atg18 complex using coarse-grained (CG) molecular dynamics (MD) simulations, and observed that the complex binds to and remodels the membrane in the proximity of Atg2 hydrophobic tunnel, allowing for the direct exchange of lipids. Overall, our data suggest that the assembly with Atg18 positions Atg2 in a PtdIns3P-dependent manner at the ER-phagophore MCS to promote lipid flux toward the elongating phagophore.

## Results

### Atg18, Atg9 and PtdIns3P provide different levels of regulation to the Atg2-mediated lipid transfer

**Figure 1:**
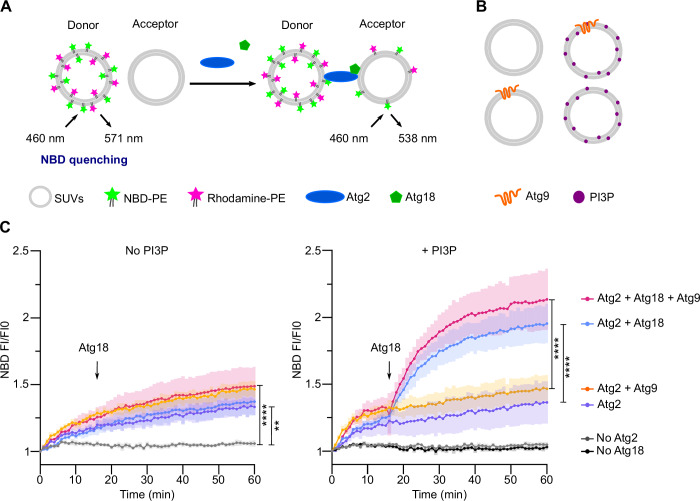
Stimulators of the Atg2-lipid transfer activity. (**A**) Schematic representation of the in vitro liposome-based lipid transfer assay. (**B**) Schematic representation of the acceptor vesicles composition in terms of PtdIns3P (PI3P) and Atg9 content. (**C**) In vitro Atg2-mediated lipid transfer assay in the presence or absence of Atg9 in acceptor vesicles with (right panel) or without (left panel) 5 mol% PtdIns3P. Atg18 was added to the reaction 15 min after Atg2 addition. The plots show the fluorescence intensity (FI) of NBD over time (min), normalized to the initial intensity at *t* = 0 (FI0) with mean values and the standard error of measurements from three independent experiments. Statistical analyses were performed by two-way ANOVA using Tukey correction and a 95% confidence interval. The adjusted *P* values for data shown in left panel (No PI3P) are as follows: No Atg2 vs Atg2, *P* = 0.0037; No Atg2 vs Atg2+Atg18+Atg9, *P* < 0.0001. The adjusted *P* values for data shown in right panel ( + PI3P) are as follows: Atg2 vs Atg2+Atg18, *P* < 0.0001; Atg2+Atg9 vs Atg2+Atg18+Atg9, *P* < 0.0001. No significant differences are not shown. Source data are available online for this figure.

Several factors regulate the lipid transfer activity of Atg2, including Atg18, Atg9 and PtdIns3P. To dissect their function and understand their interplay, we analyzed their individual contributions in an in vitro liposome-based, lipid transfer assay (Maeda et al, 2019; Valverde et al, 2019; Osawa et al, 2020; Chumpen Ramirez et al, 2023). This assay measures the transfer of lipids from donor vesicles, containing fluorescently labeled lipids, to non-labelled acceptor vesicles. In donor vesicles, the fluorescence of N-(7-nitrobenz-2-oxa-1,3-diazol-4-yl)-1,2-dihexadecanoyl-sn-glycero-3- phosphoethanolamine (NBD-PE) is quenched by the presence of a second fluorescent lipid, Rhodamine-PE, due to the proximity of the fluorophores. After lipid transfer to the acceptor vesicles, the fluorescent lipids are diluted, and the emission of the NBD fluorophore can be detected (Fig. 1A). Thus, an increase in the signal after the addition of proteins shows lipid transfer activity. To study the contribution of each factor to the lipid transfer, we purified the individual proteins from yeast and used small unilamellar vesicles (SUVs) containing or not Atg9, and/or 5 mol% PtdIns3P, as model acceptor membranes (Fig. 1B). The measurement of the NBD signal was performed immediately after the addition of Atg2 (*t* = 0). Where indicated, Atg18 was added 15 min later. We observed that Atg18 can stimulate Atg2´s basal activity only in the presence of PtdIns3P, as expected (Gómez-Sánchez et al, 2025) (Fig. 1C). In the absence of Atg18, PtdIns3P was unable to stimulate lipid transfer activity. The incorporation of Atg9 into PtdIns3P-containing acceptor vesicles only increased the lipid transfer activity of Atg2 further, when Atg18 was also present (Fig. 1C). Thus, Atg18 is a limiting factor for Atg2 activity, while PtdIns3P and Atg9 further support this stimulation.

### Identification of interaction sites between Atg2 and Atg18 by cryo-electron microscopy

**Figure 2:**
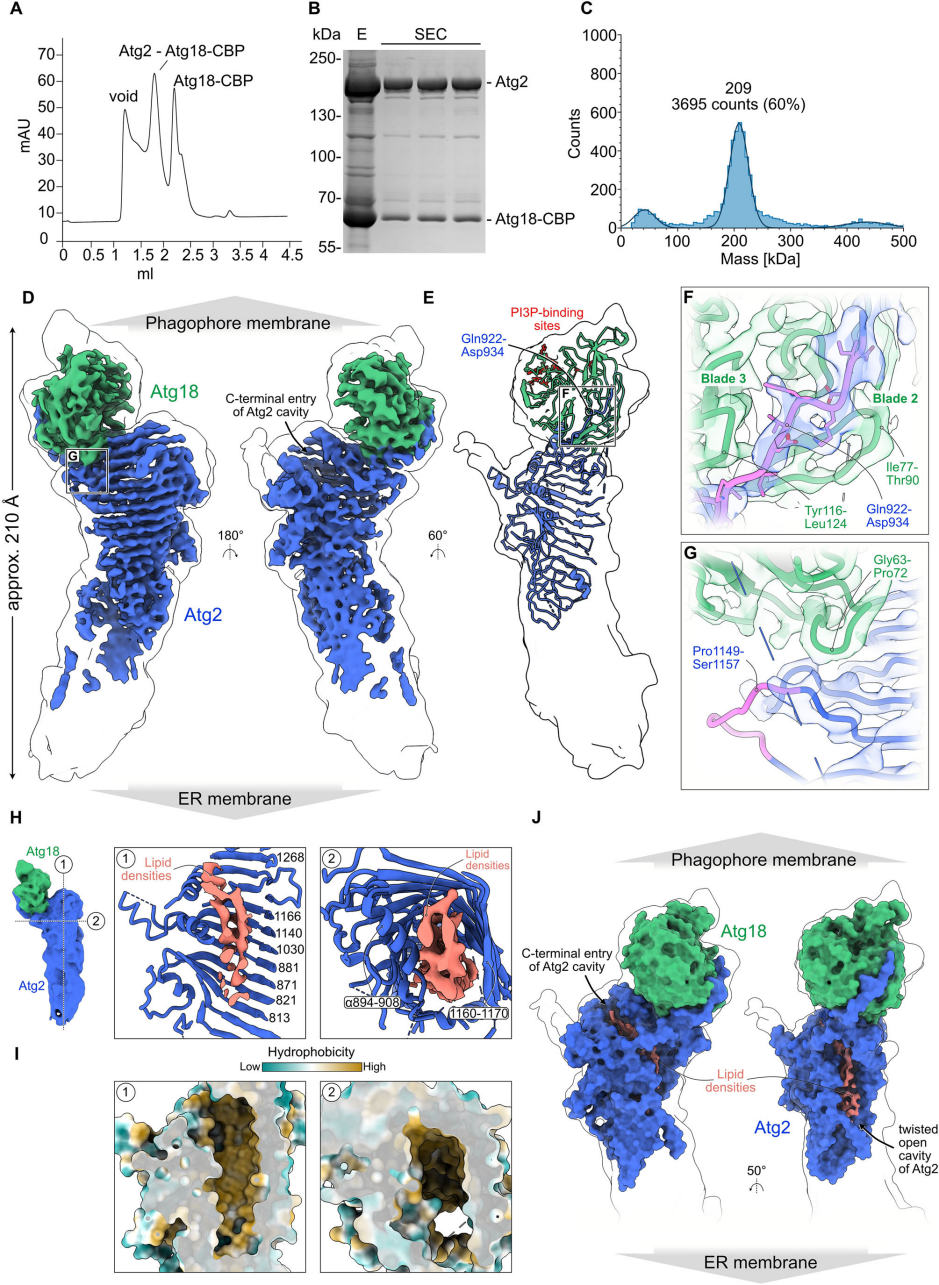
Purification and cryo-EM analysis of the Atg2-Atg18 complex. (**A**) Tandem affinity purification and size exclusion chromatography of the elution sample containing Atg18-CBP, alone and in complex with Atg2. (**B**) SDS–PAGE and Coomassie staining of samples containing Atg2-Atg18-CBP before (E: eluate from the affinity column) and after size exclusion chromatography. (**C**) Mass photometry analysis of the size exclusion chromatography peak fraction containing the Atg2-Atg18 complex. Expected molecular weight ~236 kDa. (**D**) Cryo-EM map of the Atg2-Atg18 complex viewed from two opposite orientations. The same low-pass-filtered map is shown as a transparent envelope with a black contour. Atg2 is colored in blue and Atg18 is colored in green. ER, endoplasmic reticulum. (**E**) Structure of the Atg2-Atg18 complex in cartoon representation viewed from the Atg18 face. The fitted low-pass-filtered map from (**D**) is shown as a transparent envelope with a black contour. (**F**) Close-up view of the interface between Atg2 and Atg18 framed in (**E**). The regions of Atg2 and Atg18 involved are indicated. Protein structures are shown in cartoon representation with the associated semi-transparent cryo-EM densities zoned around. Colors correspond to those in (**D**). (**G**) Close-up view of the region framed in (**D**, left panel) of the interface between Atg2 and Atg18 with the Atg2 and Atg18 regions involved indicated. Protein structures are shown in cartoon representation with the associated semi-transparent cryo-EM densities zoned around. Colors correspond to those in (**D**). (**H**) Vertical and horizontal cross-sections (1 and 2) of the Atg2-Atg18 structure through the Atg2 tunnel, as indicated in the schematic of the complex (left panel). Colors correspond to those in (**D**). Atg2 is shown in cartoon representation (blue) and cryo-EM densities corresponding to translocated lipids within the Atg2 cavity are depicted in salmon pink. Several Atg2 residues are labeled to aid orientation. (**I**) The same Atg2 cross-sections as in (**G**), with the Atg2 model shown in surface representation and colored by hydrophobicity (scale on top). (**J**) Structure of the Atg2-Atg18 complex in surface representation. The fitted low-pass-filtered map from (**D**) is shown as a transparent envelope with a black contour. Densities of the translocated lipids (salmon pink) are shown inside the twisted cavity along the Atg2 tube. Atg2 is colored in blue and Atg18 is colored in green. ER endoplasmic reticulum. Source data are available online for this figure.

**Figure EV1:**
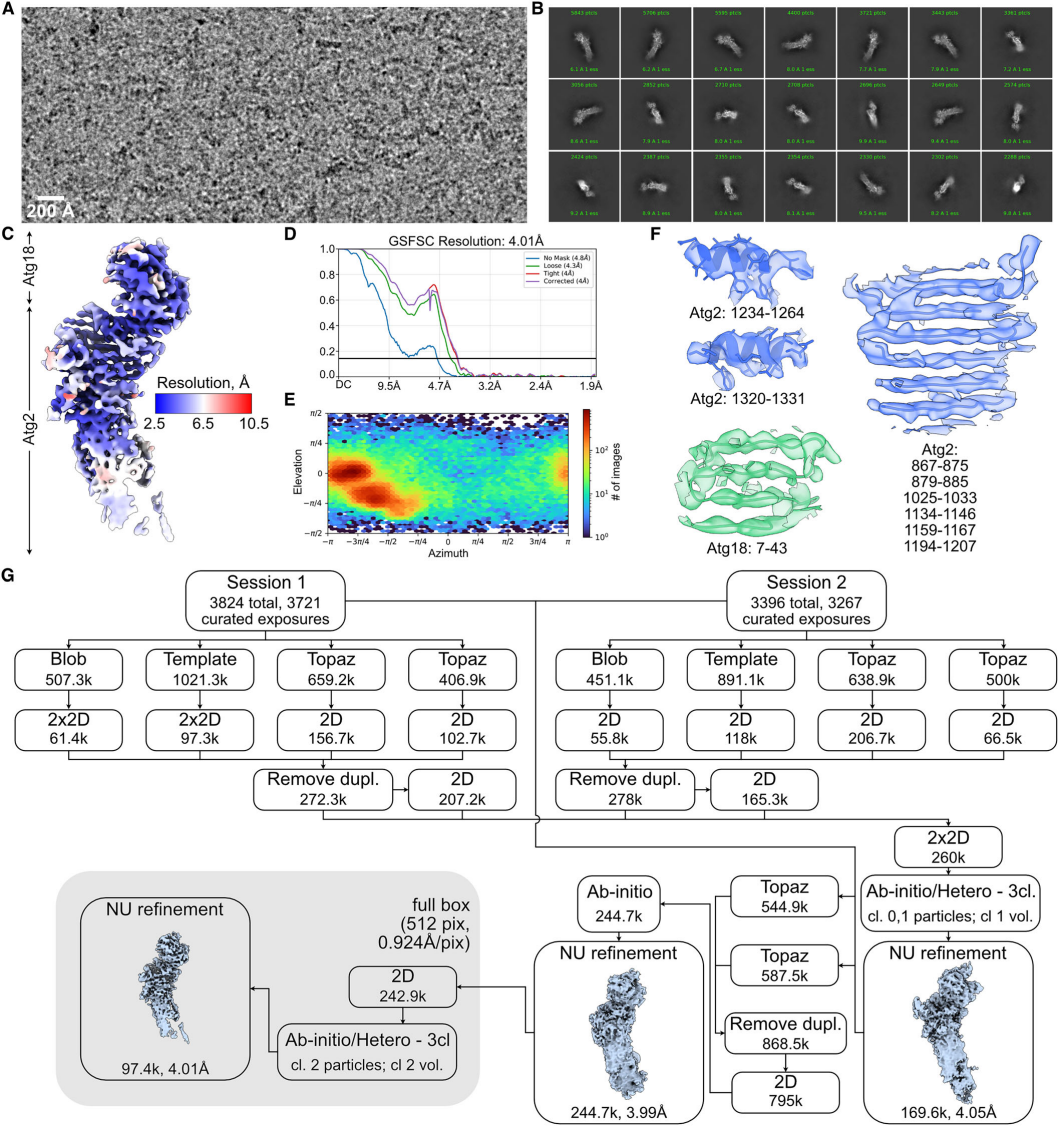
Atg2-18 complex cryo-EM single particle data analysis and validation. (**A**) Representative cryo-EM micrograph of the Atg2-Atg18 sample. (**B**) Representative 2D class averages, box size is 473 Å. (**C**) Local resolution estimation map generated using the cryoSPARC software, colored by local resolution. (**D**) cryoSPARC-generated Gold standard Fourier shell correlation (GSFSC) curve. (**E**) cryoSPARC-generated angular distribution heatmap plot. (**F**) Model/map fitting of selected segments (residues are indicated) of Atg2 (blue) and Atg18 (green). (**G**) Cryo-EM data processing pipeline. Numbers of particles used and resolutions achieved in the refinements are indicated. Cl. class, vol. volume, pix pixel.

**Figure EV2:**
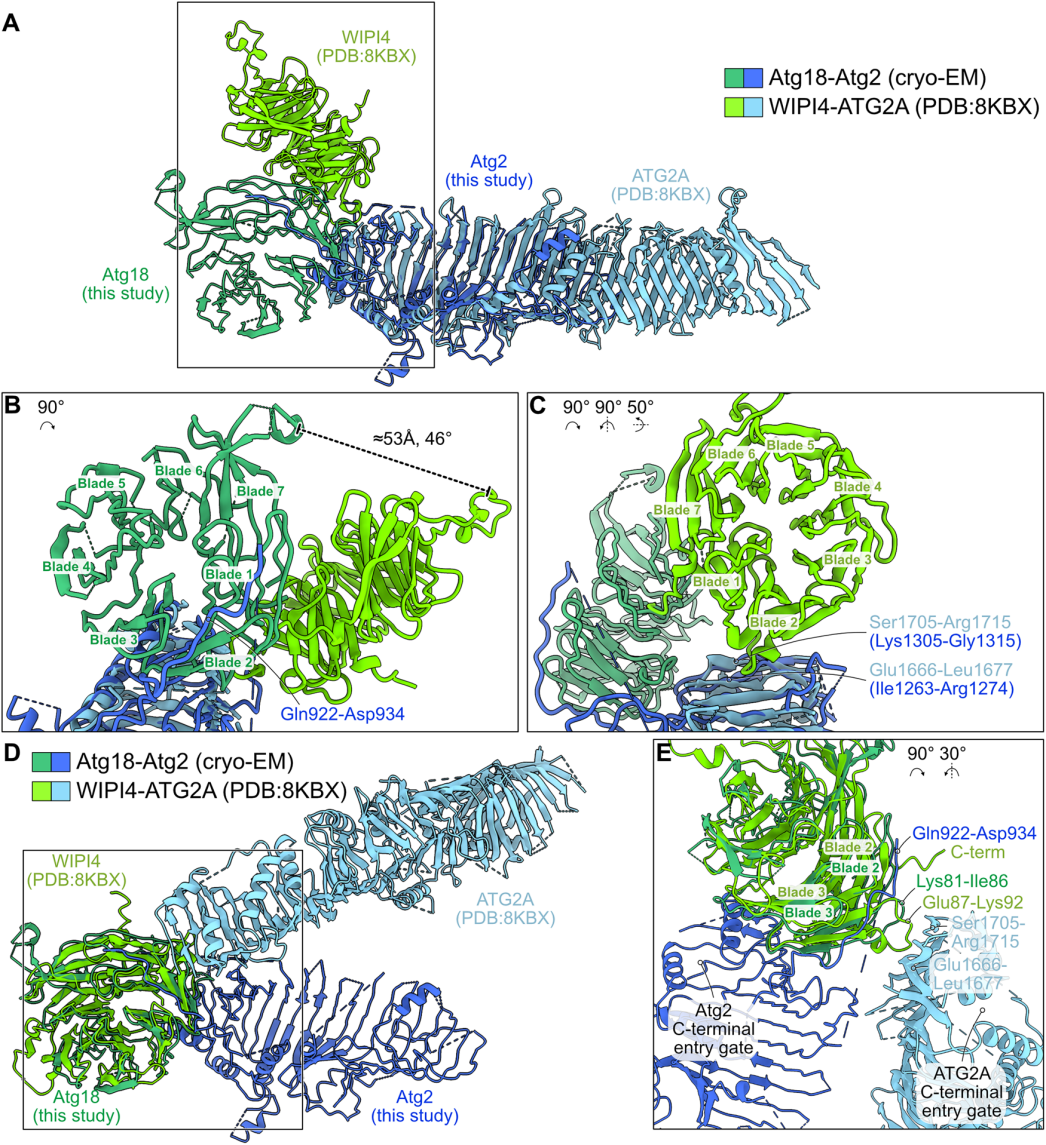
Comparison of the yeast Atg18-Atg2 structure (this study) with the human ATG2-WIPI4 structure (PDB:8KBX). (**A**) Superposition of the yeast (Atg2, dark blue, Atg18, dark green; this study) and human structure (ATG2A, light blue, WIPI4, light green; PDB:8KBX), aligned based on Atg2 using the *matchmaker* command in ChimeraX. (**B**) Close-up view of Atg18/WIPI4 from the superposition shown in (**A**). Blades of the Atg18 β-propeller are indicated. The distance between the most distal points of the β-propellers of Atg18 and WIPI4 relative to Atg2 and ATG2A, respectively, as well as the rotation angle of WIPI4 relative to Atg18, are indicated. The 922–934 region of Atg2 that is involved in the interaction with Atg18 is indicated. (**C**) Close-up view of the WIPI4 face of the superposition shown in (**A**). Blades of the WIPI4 β-propeller are indicated. The ATG2A regions that are involved in the interface with WIPI4, along with the corresponding regions of Atg2, are also highlighted. (**D**) Superposition of the yeast (Atg2, dark blue, Atg18, dark green; this study) and human structure (ATG2A, light blue, WIPI4, light green; PDB:8KBX) aligned based on Atg18 using the *matchmaker* command in ChimeraX. (**E**) Close-up view of Atg18/WIPI4 from the superposition shown in (**D**). Blades of the Atg18 and WIPI4 β-propellers are indicated. The 922–934 region of Atg2 (as in (**B**)), as well as the ATG2A regions that are involved in the interaction interface with WIPI4 are indicated (as in (**C**)).

**Figure EV3:**
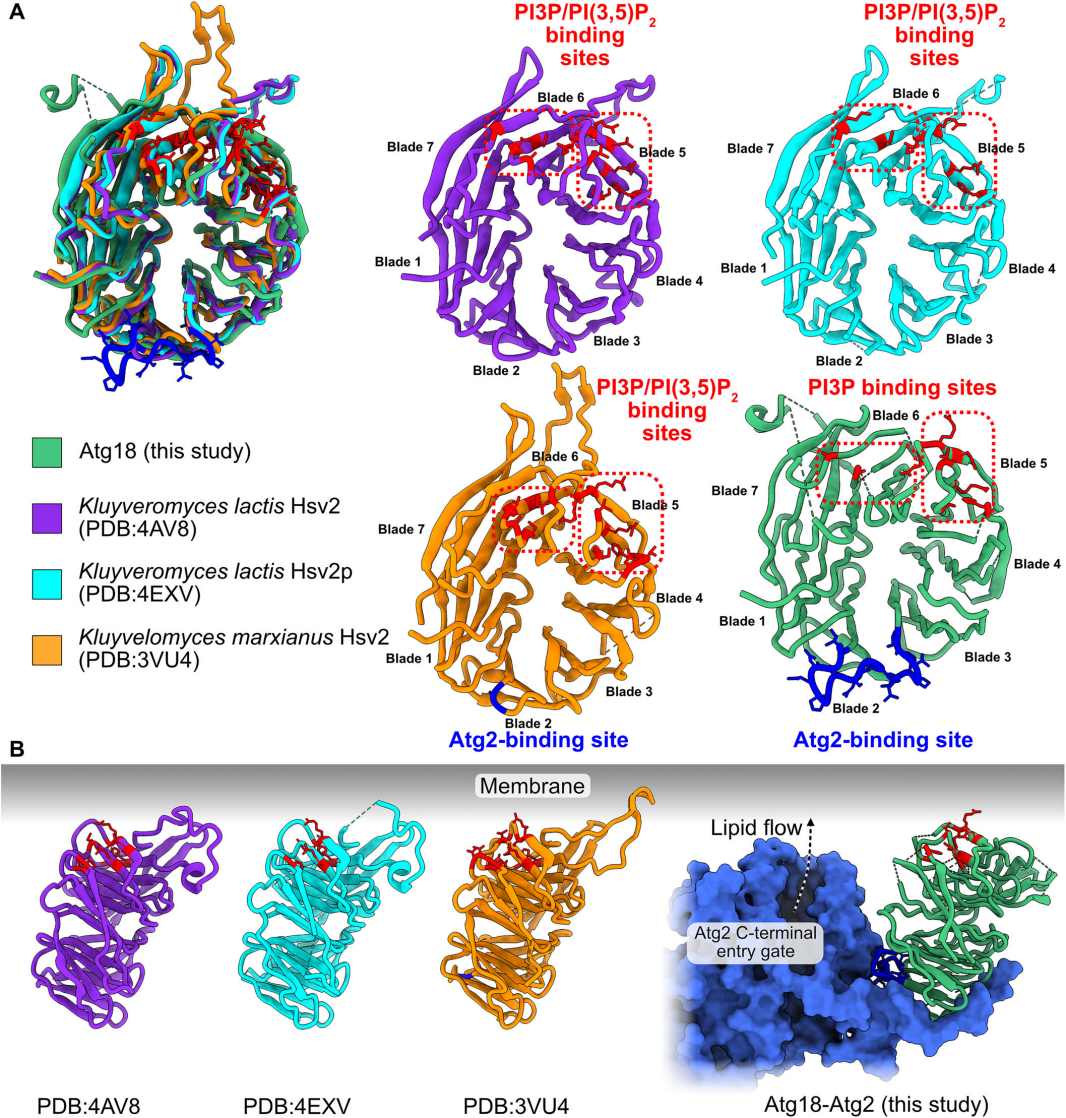
Comparison of the Atg18 and Hsv2 PROPPINs, highlighting the phosphoinositide binding sites. (**A**) Structural comparison of Atg18 (this study) and Hsv2 structures (PDB:4AV8, 4EXV, 3VU4): superposition (left top panel) and individual structures. Residues responsible for phosphoinositide binding are colored red and the phosphoinositide-binding pockets are highlighted by red dotted rounded rectangles. Amino acid residues involved in the interactions with Atg2 are colored blue. (**B**) Schematic model of Hsv2 and Atg18 interaction with membranes (gray). Left, Hsv2 proteins from (**A**) rotated by 90° with the phosphoinositide-binding sites (red) facing the membrane. Right, the Atg2-Atg18 structure (cryo-EM) with Atg18 positioned analogously to Hsv2 structures on the left. A hypothetical orientation of the Atg2-Atg18 complex with Atg18’s phosphoinositide-binding sites interacting with the membrane would permit lipid flow through the C-terminal gate of Atg2.

To gain molecular insight into the crosstalk between Atg2 and Atg18, we determined the structure of their complex. We purified TAP-tagged Atg18 from a yeast strain co-overexpressing Atg2, and subjected the sample to size exclusion and mass photometry analyses, which confirmed the presence of a preassembled Atg2-Atg18 complex (Fig. 2A–C). Samples containing the complex were subjected to cryo-EM. We determined the structure of the Atg2-Atg18 complex (Figs. 2D–H and EV1) at the overall resolution of 4 Å, locally reaching up to 3.5 Å (Fig. EV1C). The obtained cryo-EM map shows an extended Atg2-Atg18 heterodimeric complex that is ~210 Å in length. Atg2 resembles a tube-like structure formed by an extensive twisted β-sheet with distal entries at the N- and C-termini, in agreement with previous studies (Vliet et al, 2022; Wang et al, 2025). Along the structure, the tube has a twisted lateral opening toward the surrounding cytosol (Fig. 2D,E,J). The N-terminal region of Atg2 was not resolved to high resolution, however, it is visible at lower threshold levels (Fig. 2D,E, black outline), owing to apparent flexibility or structural instability of this part and the observed orientation bias in the data (Fig. EV1E). 3D variability analysis using cryoSPARC (Punjani and Fleet, 2021), as well as the “predicted local distance difference test” pLDDT score of the corresponding AlphaFold3 model of the Atg2-Atg18 complex, indicated increased flexibility and structural instability of the Atg2 N-terminal region (Appendix Fig. S1). The predicted peripheral loops and the C-terminal α-helical domains of Atg2 with similarly low AlphaFold3 pLDTT scores were also not resolved by cryo-EM (Fig. EV2A; Appendix Fig. S2A). The internal hydrophobic cavity of Atg2 was almost entirely filled with diffuse densities that we assigned as lipids (Fig. 2H–J). Atg18, resembling a 7-blade β-propeller, binds via its blade 2 (residues 63–72) to the Atg2 β-sheet at the C-terminal end (first five β-strands) behind the tube entry (Figs. 2D,E and EV2A,B). The Atg2 region potentially supporting this interaction, includes the residues 1149–1157, forming a loop positioned in proximity to residues 63–72 of Atg18 located in the blade 2 β-propeller (Fig. 2G). Blade 2 also contains the residues P72 and R73, previously described as essential for interaction between Atg2-Atg18 (Watanabe et al, 2012) and forms an interface with the Atg2 β-sheet tubular core. Notably, while human Atg18 homologue WIPI4 also uses blade 2 for the interaction within the WIPI4-ATG2A complex, it contacts ATG2A through a different set of residues that do not align with those from Atg18 and is positioned at a different orientation with respect to ATG2A (Fig. EV2B–E) (Wang et al, 2025). Except for the interactions with the Atg2 β-sheet wall, Atg18 binds to an extended loop in Atg2, i.e., residues 907-1024, which is mostly unresolved in our map. However, its structured fragment, i.e., residues 922–934, interacts with Atg18 blades 2 and 3, and encloses the β-propeller from the side opposite to the interface with the Atg2 β-sheet (Figs. 2D–F and EV2; Appendix Fig. S2). This interface is in proximity to the residues 89–95 of Atg18, a region previously described as essential for the interaction between both proteins in vivo (Rieter et al, 2013). Interestingly, sequence alignments show that Atg18 residues 89–95 overlap with a hydrophobic region conserved in other PROPPINs (Appendix Fig. S3A), previously identified in WIPI3 as binding sites for the WIR-motif in ATG2A (Ren et al, 2020). Our structure revealed that the interaction interface between the Atg2 region 922–934 extends along these conserved hydrophobic sites on Atg18 (Appendix Fig. S3B,C), similar to the previously reported interaction between WIPI3 and the ATG2A WIR peptide (Ren et al, 2020). Intriguingly, the proposed region as a WIR-motif in Atg2 includes the region 1193-1201 (Ren et al, 2020), which in our structure is located on the opposite side of the protein relative to the Atg18 interface and does not appear to participate in the binding with Atg18 (Appendix Fig. S3B). Importantly, our structure shows that the PtdIns3P-binding FRRG motif in Atg18, located in blades 5 and 6 (Baskaran et al, 2012; Krick et al, 2012; Watanabe et al, 2012), is positioned opposite to the Atg2 binding site and is oriented toward the same direction as the C-terminal gate of the Atg2 hydrophobic cavity, allowing them to simultaneously interact with the membrane (Figs. 2E and  EV3).

### The newly identified Atg18-binding region in Atg2 is essential for autophagy

**Figure 3:**
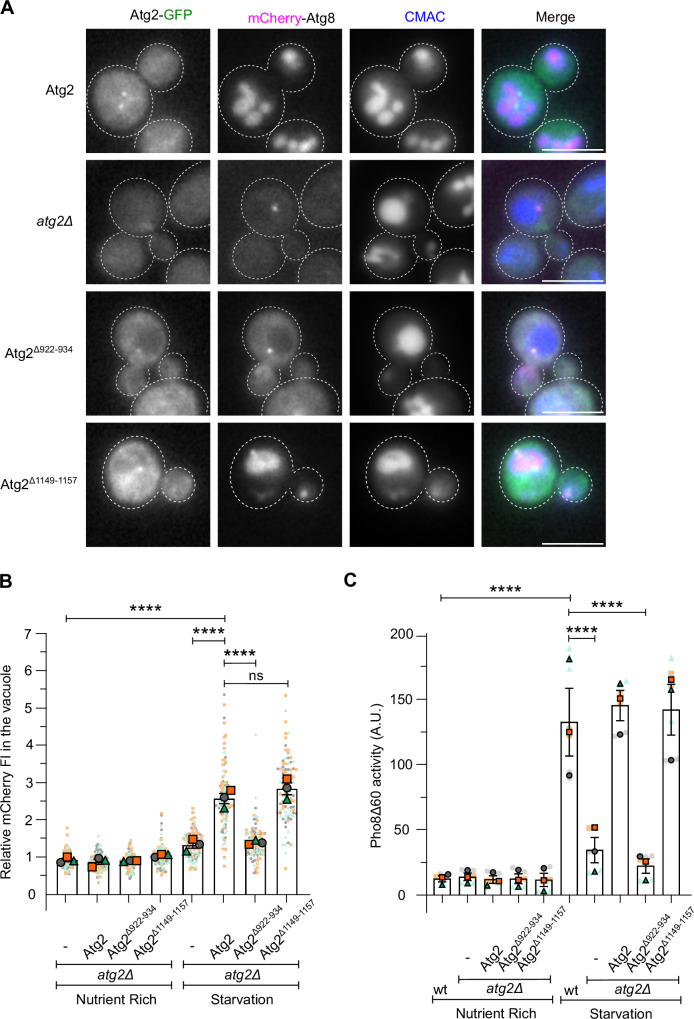
Flux of autophagy in strains expressing mutant versions of Atg2. (**A**) mCherry-Atg8 delivery to the lumen of the vacuole in cells during starvation. Integrative plasmids carrying Atg2 or the indicated mutants fused to GFP and under the control of the endogenous promoter were transformed in an *atg2Δ* strain also expressing mCherry-Atg8. Cells were grown in rich media or nitrogen starved by incubation in SD-N medium for 3 h. Vacuoles were stained with CMAC. The figure shows representative cells incubated under starvation conditions. Cells boundaries are marked (white dotted lines). BF Bright field. Images correspond to representative single planes (Z = 0.25 μm). Scale bar, 5 μm. (**B**) Quantification of (**A**). mCherry fluorescence intensity was measured in the lumen of the vacuole and was normalized to the average signal measured under nutrient-rich conditions. The plot shows the quantification performed in 100 cells from 3 independent experiments, the mean and the standard error. Statistical analysis was performed by two-way ANOVA using Dunnett´s correction for multiple comparisons, and a 95% confidence interval. The adjusted *P* values are as follow: *atg2Δ* Atg2 (CU15159) Starvation vs *atg2Δ* (CU15120) Starvation, *P* < 0.0001; *atg2Δ* Atg2 (CU15159) Starvation vs *atg2Δ* (CU15120) Nutrient rich, *P* < 0.0001; *atg2Δ* Atg2 (CU15159) Starvation vs *atg2Δ* Atg2^Δ922-934^ (CU15121*)* Starvation, *P* < 0.0001; *atg2Δ* Atg2 (CU15159) Starvation vs *atg2Δ* Atg2^Δ1149−1157^ (CU15158) Starvation, *P* = 0.1697 (no significant = ns). (**C**) Pho8Δ60 activity assay from cells expressing the Atg2 variants. The Pho8Δ60 *atg2Δ* strain (*atg2Δ*) (CU15300) was transformed with integrative plasmids carrying the wild-type version of Atg2 (CU16293) or the indicated mutants (CU16292 and CU16302) and under the control of the endogenous promoter. The Pho8Δ60 strain (CU10489) was included as a wild-type (wt) control. Cells were grown in YPD and analyzed under nutrient-rich conditions or nitrogen starved by incubation in SD-N for 3 h. The plot shows the enzymatic activity calculated as arbitrary units (A.U.). The data correspond to the mean value and the standard error of measurements from three independent experiments. The statistical analysis was performed by two-way ANOVA using Dunnett´s correction for multiple comparisons, and a 95% confidence interval. The adjusted *P* values are as follows: wt (CU10489) Starvation vs wt (CU10489) Nutrient rich, *P* < 0.0001; wt (CU10489) Starvation vs *atg2Δ* (15300) Starvation, *P* = 0.0001; wt (CU10489*)* Starvation vs *atg2Δ* Atg2^Δ922–934^ (CU16292) Starvation, *P* < 0.0001. No significant differences are not shown. Source data are available online for this figure.

To test the functional role of the two loop regions that we identified in Atg2 at the interfaces with Atg18, we generated the corresponding deletion mutants in Atg2, i.e., Atg2^∆922-934^ and Atg2^∆1149-1157^, and fused them with GFP to examine their localization and functionality in vivo. The wild-type and mutant variants were expressed in an *atg2Δ* strain carrying the PAS/phagophore marker mCherry-Atg8 to analyze the flux of autophagy. Atg8 is mostly cytosolic under nutrient-rich conditions, while after autophagy induction, it is recruited to the phagophore and in part delivered into the lumen of the vacuole upon autophagosome fusion, evidenced by the accumulation of mCherry within this organelle (Fig. 3A). We found that in contrast to cells expressing wild-type Atg2, the deletion of the residues 922–934 abolished the delivery of Atg8 to the vacuoles, as no mCherry signal was detected in the vacuole lumen after 3 h of nitrogen starvation (Fig. 3A,B). Cells expressing Atg2^∆1149–1157^ showed no defects in autophagy (Fig. 3A,B), suggesting that the Atg2-Atg18 interaction is mainly provided by residues 922–934 in Atg2. Given that the analysis was performed using fluorescent tags fused to the Atgs, we wonder whether this could affect their function and decided to test autophagy using the Pho8Δ60 assay, a more sensitive and quantitative method. This assay measures the phosphatase activity of Pho8Δ60 (Guimaraes et al, 2015), a truncated version of the vacuolar alkaline phosphatase that remains inactive in the cytosol under normal growth conditions. During autophagy, Pho8∆60 delivery into the vacuole by autophagosomes results in its activation, which can be detected by measurement of its enzymatic activity. We indeed found that the tagging of Atg8 with mCherry resulted in ~50% reduction in the Pho8Δ60 activity, while the GFP tagging of Atg2 did not impair the flux of autophagy (Appendix Fig. S4A). We then used the Pho8Δ60 assay to test our mutants and analyzed autophagy in Pho8Δ60 *atg2Δ* cells carrying integrative plasmids that express the Atg2 variants under the control of the endogenous promoter. After 3 h of nitrogen starvation, cells expressing wild-type Atg2 had increased phosphatase activity in comparison to the nutrient-rich conditions (Fig. 3C). In contrast, the *atg2Δ* mutant had no increase in Pho8Δ60 activity, indicating that the autophagy flux is blocked. Consistent with our fluorescence microscopy observations, deletion of residues 922–934 in Atg2 drastically reduced Pho8Δ60 activity, while deletion of amino acids 1149–1157 did not impair autophagy (Fig. 3C). These observations were also replicated in samples from cells expressing the GFP-tagged versions of Atg2 (Appendix Fig. S4B).

**Figure 4:**
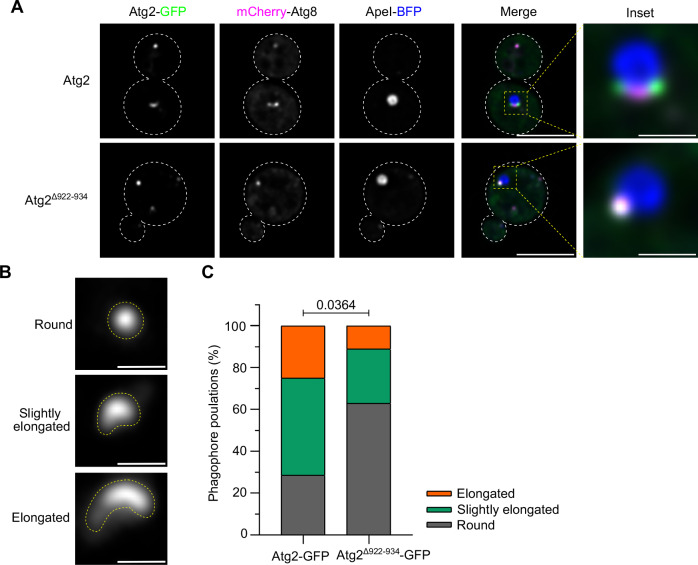
Giant Ape1 assay in cells expressing Atg2 variants. (**A**) Fluorescence microscopy analysis of the phagophore elongation. BFP-Ape1 was overexpressed using a *CUP1* promoter in *atg2Δ* cells expressing genomically tagged mCherry-Atg8 and carrying integrative plasmids expressing the indicated Atg2-GFP variants under the control of the endogenous *ATG2* promoter. Cells were nitrogen starved in SD-N medium supplemented with 250 μm CuSO_4_ for 2 h before imaging. Deconvolved representative images are shown. The insets show the phagophores (magenta) around or next to the giant Ape1 cargo (blue), and the overlapping signal between Atg2 and Atg8 (white). Cells boundaries are marked with white dotted lines, and the inset region is indicated with yellow dotted lines. Scale bar in the Merge panels, 5 μm. Scale bar in the Inset panels, 1 μm. (**B**) Deconvolved representative images of the phagophore classification according to their shape and elongation. The phagophores were classified according to the ratio between the width and height as round (ratio = 1–1.14), slightly elongated (ratio = 1.15–1.4) or elongated (ratio >1.4). The signal corresponds to mCherry-Atg8 and the boundaries of the phagophore are marked (yellow dotted lines). Scale bar: 1 μm. (**C**) Quantification of the phagophore populations in cells analyzed in (**A**), according to the classification described in (**B**). The plot represents the data from the analysis of ≥27 phagophores per strain. The statistical significance was determined by Chi-Square analysis with a 95% interval confidence. Source data are available online for this figure.

The Atg2-Atg18 complex plays a key role in supplying the phagophore with lipids to promote its elongation. Because the Atg2^∆922–934^ mutant showed a strong autophagy defect, we focused on this variant to assess its impact on the elongation of this cisterna. The phagophore can be distinctively visualized using the giant Ape1 assay. Overexpression of Ape1 generates a giant cargo and the phagophores elongation around the giant Ape1 can be resolved by fluorescence microscopy (Suzuki et al, 2013). We therefore analyzed the phagophore size in cells expressing mCherry-Atg8, giant BFP-Ape1, and Atg2-GFP or Atg2^∆922-934^-GFP (Fig. 4A). We measure the diameter and longitude of the phagophore as parameters to define the structures as punctuate, slightly elongated, or elongated (Fig. 4B), and quantified their relative populations. In cells expressing Atg2, almost 50% of the phagophores were slightly elongated and 25% completely elongated. In contrast, cells expressing the mutant version Atg2^∆922–934^ had only a minor fraction of elongated phagophores (around 10%) and a higher population of punctuate structures (> 60%) (Fig. 4C), suggesting that the Atg2 mutant is strongly defective in promoting lipid flux into the phagophore. Given that the size of the giant Ape1 cargo can be variable, we compared its size in both strains to rule out the possibility that the phagophore elongation is biased by the cargo size. We found no significant differences in the area or diameter of the giant Ape1 cargoes between strains (Appendix Fig. S5A,B), nor correlations between the Ape1 cargo size and phagophore elongation (Appendix Fig. S5C,D).

### The ∆922–934 truncation in Atg2 impairs Atg18 recruitment to the PAS

**Figure 5:**
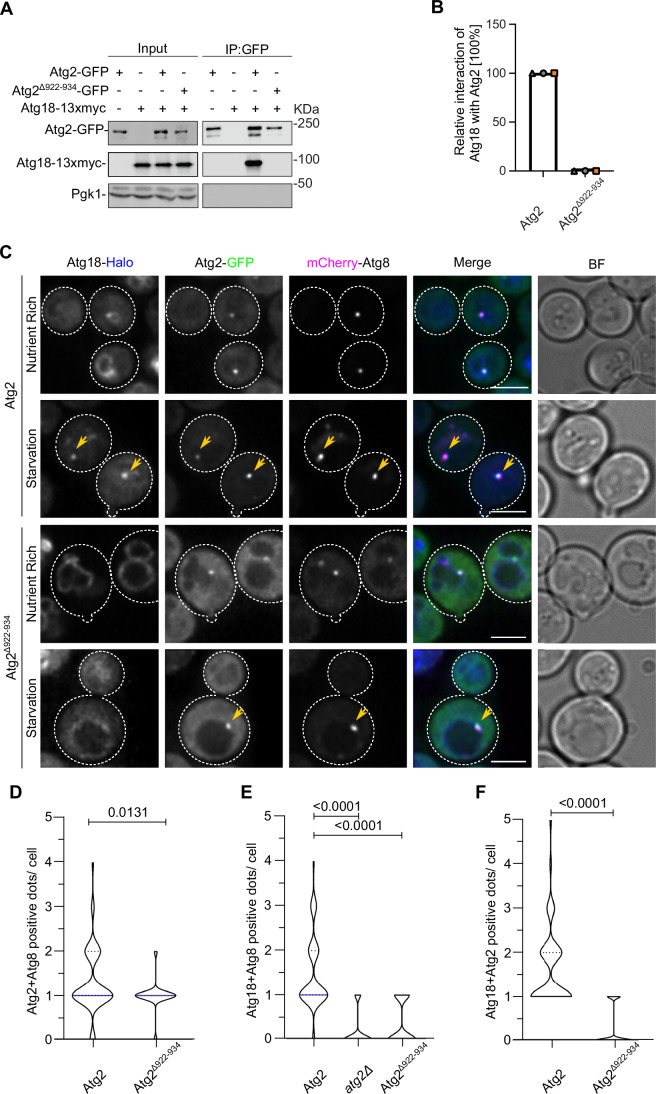
Analysis of Atg18-Atg2 interaction and PAS recruitment in cells expressing the Atg2 variants. (**A**) The *atg2Δ* and *atg2Δ* Atg18-13xmyc strains were transformed with integrative plasmids expressing Atg2-GFP or Atg2^∆922-934^-GFP, or an empty vector as negative control. Cells were grown in YPD to log phase and starved in SD-N medium for 1 h. The Atg2 variants were immunoprecipitated from total cell lysates using GFP-Trap beads (IP:GFP). Proteins eluted from the beads were analyzed by western blot using anti-GFP, anti-MYC or anti-Pgk1 antibodies. Pgk1 served as the loading control for the total lysates. (**B**) Quantification of (**A**). The plot shows the % of co-immunoprecipitation of Atg18-13xmyc with Atg2^∆922-934^-GFP relative to Atg2-GFP, considered as 100% binding. The plot displays the mean of three independent experiments. (**C**) Endogenous Atg18 was tagged with HaloTag in *atg2Δ* strains expressing genomically mCherry-Atg8 and carrying integrative plasmids expressing Atg2-GFP or Atg2^∆922-934^-GFP under the control of the endogenous *ATG2* promoter. Cells were grown in YPD until early log-phase and then transferred in SD-N medium for 1 h to induce autophagy or in YPD as control (nutrient-rich). Cells were stained with the HaloTag ligand FJX650 before live-cell imaging. The figure shows single stacks representative of each strain and growth condition. Overlapping signals at the PAS during starvation are indicated with yellow arrows. Cells boundaries are marked with white dotted lines. BF: Bright field. Scale bar: 5 μm. (**D**–**F**) Quantification of (**C**). The plots show the distribution of cells according to the amount of colocalizing puncta. The median is indicated in blue and the quartiles are indicated with black dot lines. The data correspond to the quantification of at least 50 cells from three biological replicates. (**D**) The plot shows the frequency distribution of Atg2-GFP or Atg2^∆922-934^-GFP-positive dots overlapping with mCherry-Atg8 per cell. The statistical analysis was performed using two-tailed Mann–Whitney *U* test with 95% confidence intervals. (**E**) The plot shows the frequency distribution of Atg18-Halo dots per cell overlapping with the PAS, positive for mCherry-Atg8 and Atg2-GFP (wild-type or mutant) or positive for Atg8 in *atg2Δ* strain. The statistical analysis was performed by Kruskal–Wallis test with Dunn´s correction for multiple comparisons and 95% confidence interval. (**F**) The plot shows the frequency distribution of dots positive for Atg2-GFP or Atg2^∆922–934^-GFP overlapping with Atg18-Halo per cell. The statistical analysis was performed using two-tailed Mann–Whitney *U* test with 95% confidence intervals. Source data are available online for this figure.

**Figure EV4:**
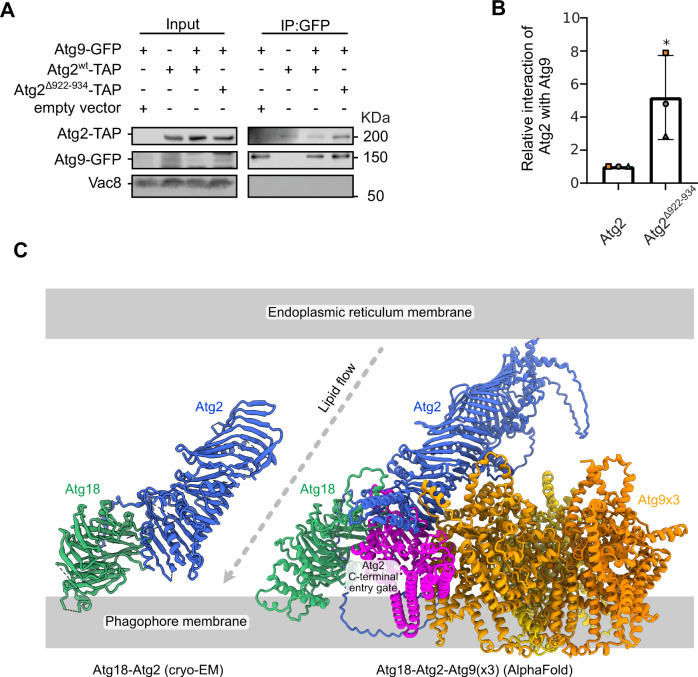
Possible interaction of Atg2-Atg18 with Atg9. (**A**) The *atg2Δ* and *atg2Δ* Atg18-13xmyc strains expressing endogenous Atg9-GFP and transformed with integrative plasmids carrying Atg2-TAP or Atg2^Δ922-934^-TAP, or an empty vector were grown in YPD to a log phase and starved for 1 h in SD-N medium to induce autophagy. Atg9-GFP and associated proteins were co-immunoprecipitated from total cell lysates using GFP-Trap beads (IP:GFP). Proteins eluted from the beads were analyzed by western blot using anti-GFP, anti-TAP or anti-Vac8 antibodies. Vac8 served as the loading control for the total lysates. (**B**) Quantification of (**A**). The graph shows the ratio of co-immunoprecipitation of Atg2^Δ922-934^-TAP with Atg9-GFP relative to that of Atg2-TAP. The graph shows the mean value and the standard deviation of three independent experiments. Statistical analysis was conducted by two-tailed unpaired *t* test with 95% confidence intervals. *P* value is as follows: Atg2^∆922-934^ (PVY129) vs Atg2 (RGY590), *P*  =  0.0465. (**C**) Hypothetical positioning of Atg2-Atg18 complex at the membrane (left, cryo-EM structure) and the AlphaFold3 model of the Atg2-Atg18 complex in association with the Atg9 trimer, positioned similarly in the membrane. The fragment of Atg2 corresponding to the ATG9A-interacting ATG2A fragment 4 (van Vliet et al, 2022) is colored in magenta. The orientation of the proteins in both the cryo-EM structure and the AlphaFold3 model allows the simultaneous interaction of Atg2 with Atg9 and Atg18, membrane binding by Atg18, and positioning of the Atg2 C-terminal channel gate toward the membrane for lipid transfer (gray dotted arrow).

Our functional analyses suggest that truncation of the 922–934 region in Atg2 could impair its interaction with Atg18. Therefore, we first examined whether this was indeed the case. To this end, we expressed Atg18–13xMyc in cells also carrying Atg2-GFP or Atg2^∆922-934^-GFP and performed GFP immunoprecipitations from total cell lysates. We found that Atg18 co-eluted only with wild-type Atg2 as expected (Rieter et al, 2013; Gómez-Sánchez et al, 2018, 2025), but not with Atg2^∆922-934^ (Fig. 5A,B). Next, we analyzed whether the 922–934 truncation affected the recruitment of Atg18 to the phagophore. For this, we generated strains expressing Atg18-HaloTag, mCherry-Atg8, and the GFP-tagged Atg2 variants. As control, we tested the flux of autophagy in cells expressing Atg18-Halo and corroborated that the HaloTag did not impair this process (Appendix Fig. S4A). We therefore monitored the localization of Atg18–HaloTag in cells expressing either Atg2-GFP or Atg2^∆922-934^-GFP. Under nutrient-rich conditions, Atg18 localized mainly on the vacuolar membrane as expected, due to the presence of PtdIns3P and PtdIns(3,5)P₂, while both versions of Atg2 remained largely cytosolic and were occasionally detected at the mCherry-Atg8-positive PAS (Fig. 5C). After induction of autophagy, Atg2-GFP and Atg2^∆922-934^-GFP were most frequently detected at the PAS (Fig. 5C). Although the brightness of the puncta positive for both Atg8 and Atg2^∆922–934^ was reduced compared to the expression of wild-type Atg2, the ∆922–934 truncation did not abolish the recruitment of Atg2 to the PAS (Fig. 5D). In contrast, Atg18 localization to Atg8- and Atg2-positive structures was strongly reduced in cells carrying Atg2^∆922–934^, similar to the phenotype observed in an *atg2Δ* strain and in clear contrast to the cells expressing wild-type Atg2 (Fig. 5E,F). Considering that Atg9 participates in the formation of this complex and the recruitment of Atg18 to the PAS requires the prior interaction between Atg2 and Atg9 (Gómez-Sánchez et al, 2018), it remains unclear whether these observations resulted from the specific disruption of the Atg2–Atg18 interface. Therefore, we performed an in vivo pull-down experiment to test the interaction between the Atg2^∆922-934^ mutant and Atg9. We found that the mutant co-elutes with Atg9, and in a higher proportion than the wild-type (Fig. EV4A,B), indicating that residues within the segment 922–934 are involved in the specific interaction between Atg2 and Atg18. Consistent with these observations, the AlphaFold3 prediction of the Atg2-Atg18-Atg9 complex reproduced the orientation of Atg2 and Atg18 observed in our cryo-EM structure. It also showed that Atg2 interacts with the Atg9 trimer via the C-terminal α-helical domains, which mediate the interaction between these proteins (Vliet et al, 2022), and are attached to the tunnel β-sheet wall at the side opposite to the Atg18 binding interface (Fig. EV4C).

**Figure 6:**
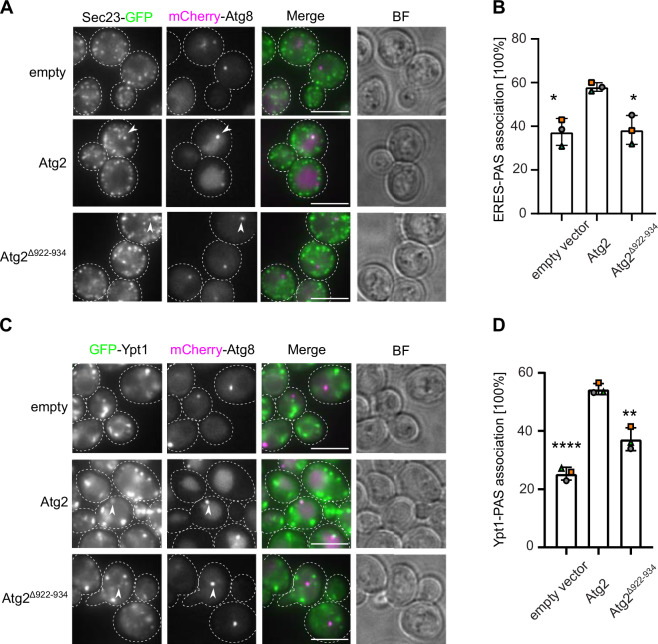
Effect of the 922–934 deletion in Atg2 on ERES-phagophore contact site. (**A**) The *atg2Δ* strains expressing endogenous Sec23-GFP, mCherry-Atg8 and transformed with an integrative empty vector (RGY541), or a plasmid carrying Atg2-TAP (RGY637) or Atg2^Δ922-934^-TAP (PVY122), were grown in YPD and then transferred in SD-N medium for 1 h to induce autophagy. Images were collected with a Oxford Nanoimager microscope system. Associations between ERES and PAS are indicated with white arrowhead. Cells boundaries are marked (white dotted lines). BF: Bright field. Scale bar: 5 μm. (**B**) Quantification of (**A**). The percentage of mCherry-Atg8-positive structures associated with the ERES was determined by analyzing ≥100 cells from three independent experiments. The plot shows the mean value and the standard error of the three independent measurements. Statistical analysis was conducted by two-tailed unpaired *t* test with Welch’s correction with 95% confidence intervals. *P* values are as follows: Atg2^∆922-934^ (PVY122) vs Atg2 (RGY637), *P*  =  0.0278; empty vector (RGY541) vs Atg2 (RGY637), *P*  =  0.0206. (**C**) The *atg2Δ* cells expressing endogenous GFP-Ypt1, mCherry-Atg8 and transformed with an empty vector (RGY1015), or a plasmid carryring Atg2-TAP (PVY125) or Atg2^Δ922-934^-TAP (PVY126), were examined as in (**A**). White arrowheads denote Ypt1 localization to the PAS. Cells boundaries are marked (white dotted lines). BF: Bright field. Scale bar: 5 μm. (**D**) Quantification of (**C**). The percentage of GFP-Ypt1-positive mCherry-Atg8 foci was determined by analyzing ≥100 cells from three independent experiments. The plot shows the mean value and the standard error of the three independent measurements. Statistical analysis was conducted by two-tailed unpaired *t* test with Welch’s correction with 95% confidence intervals. *P* values are as follows: Atg2^∆922-934^ (PVY126) vs Atg2 (PVY125), *P*  =  0.0074 ; empty vector (RGY1015) vs Atg2 (PVY125), *P*  <  0.0001. Source data are available online for this figure.

We recently showed that the recruitment of Atg18 to the PAS requires the Ypt1-mediated activation of the PI3K-I (Gómez-Sánchez et al, 2025). Thus, we tested the Ypt1 and Trs85 recruitment to the PAS, as well as the association of the PAS to the ER and the ERES, in cells expressing the wild-type and mutant Atg2. We found that the association of the PAS to the ER and the localization of the Trs85 were not affected by the expression of the mutant Atg2 (Appendix Fig. S6). However, the association of the PAS to the ERES was reduced in cells expressing Atg2^Δ922-934^ (Fig. 6A,B) as well as the recruitment of Ypt1 (Fig. 6C,D), possibly due to the role of the ERES in the TRAPPIII activation (Gómez-Sánchez et al, 2025).

### Atg2^Δ922-934^ is insensitive to the Atg18-mediated stimulation of lipid transfer

**Figure 7:**
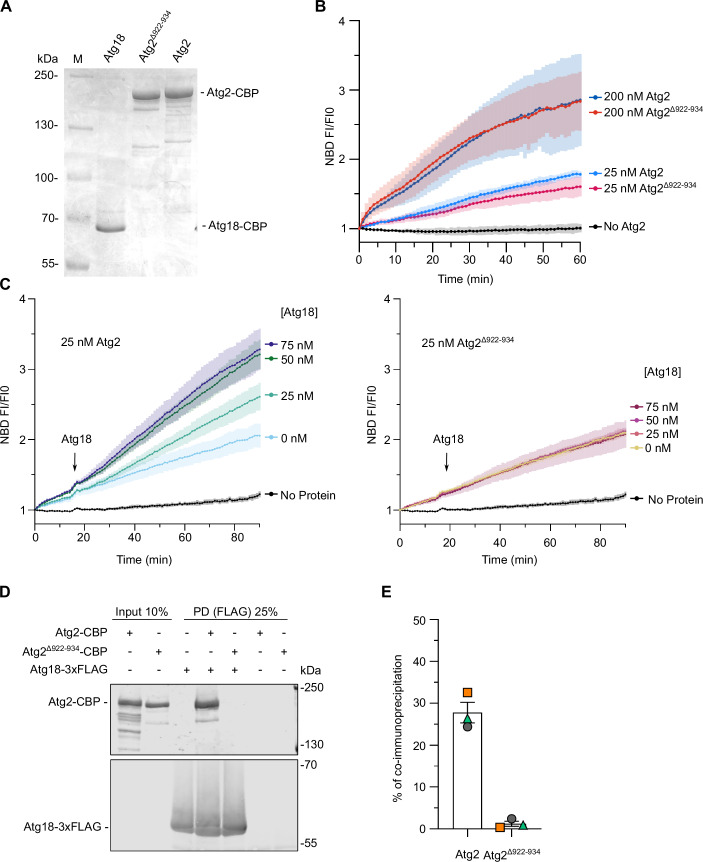
Effect of the 922–934 deletion in Atg2 on in vitro interactions and lipid transfer activity. (**A**) SDS–PAGE and Coomassie blue staining of the purified proteins tested in the in vitro assays. The figure shows representative elution fractions from tandem affinity purifications of the indicated proteins. The eluted proteins are fused to CBP. M: Protein molecular weight marker. (**B**) In vitro lipid transfer assay performed with purified Atg2 or Atg2^∆922-934^ at different protein concentrations. Liposomes incubated in the absence of proteins were included as control. (**C**) Effect of Atg18 in the activity of Atg2 or Atg2^∆922-934^. The lipid transfer assays were performed with 25 nM wild-type Atg2 (left panel) or the mutant version (right panel), in the absence or presence of Atg18 at the indicated concentrations. The NBD signal was measured immediately after the addition of Atg2 and Atg18 was added 15 min later. (**B**, **C**) The plots show the fluorescence intensity (FI) of NBD over time (min), normalized to the initial intensity at *t* = 0 (FI0), corresponding to the mean values and the standard error of measurements from three independent experiments. (**D**) In vitro pull-down (PD) assay showing the interaction between Atg18 and either wild-type Atg2 or Atg2^∆922-934^. Atg18-3×FLAG from cell lysates was immobilized on beads and incubated with purified Atg2–CBP or Atg2^∆922-934^-CBP to assess protein binding. As controls, Atg2–CBP and Atg2^∆922-934^-CBP were incubated with beads exposed to lysates lacking Atg18-3×FLAG. Proteins retained on the beads were eluted (PD) and analyzed by SDS–PAGE and Western blotting using anti-FLAG and anti-TAP antibodies. The input lanes correspond to 10% of the total purified protein used. The image shows a representative western blot from three independent experiments. (**E**) Quantification of (**D**). The plot shows the percentage of Atg2 co-eluted with Atg18 relative to the input. Values were calculated based on the input signal and normalized to the Atg18-3×FLAG signal detected under each condition. Data represent mean ± SD from three independent experiments. Source data are available online for this figure.

Given that the mutation in Atg2 seems to affect the integrity of the membrane contact site (Fig. 6), we could not exclude that the 922–934 truncation in Atg2 cause a structural instability compromising its enzymatic function. We therefore investigated if the strong phenotypes we observed in vivo arise from defects in Atg2-Atg18 complex formation or conformation, or from an intrinsic loss of the lipid-transfer activity. To this end, we purified Atg2-TAP and Atg2^∆922-934^-TAP from yeast (Fig. 7A) and performed the in vitro lipid transfer assays. We observed that Atg2 and Atg2^∆922-934^ exhibited a similar basal activity that was concentration dependent (Fig. 7B). Importantly, while the addition of Atg18 strongly stimulated the transfer activity of Atg2, the one of Atg2^∆922-934^ remained unaffected even in the presence of a four-fold excess of Atg18 (Fig. 7C). To determine whether this defect resulted from impaired complex formation, we performed an in vitro pull-down by incubating immobilized Atg18–3×FLAG on beads with the purified Atg2 variants. Consistent with our in vivo observations, deletion of residues 922–934 completely abolished the interaction between Atg18 and Atg2 (Fig. 7D,E).

### Molecular dynamics simulations reveal lipid-delivery capability of the resolved structure

**Figure EV5:**
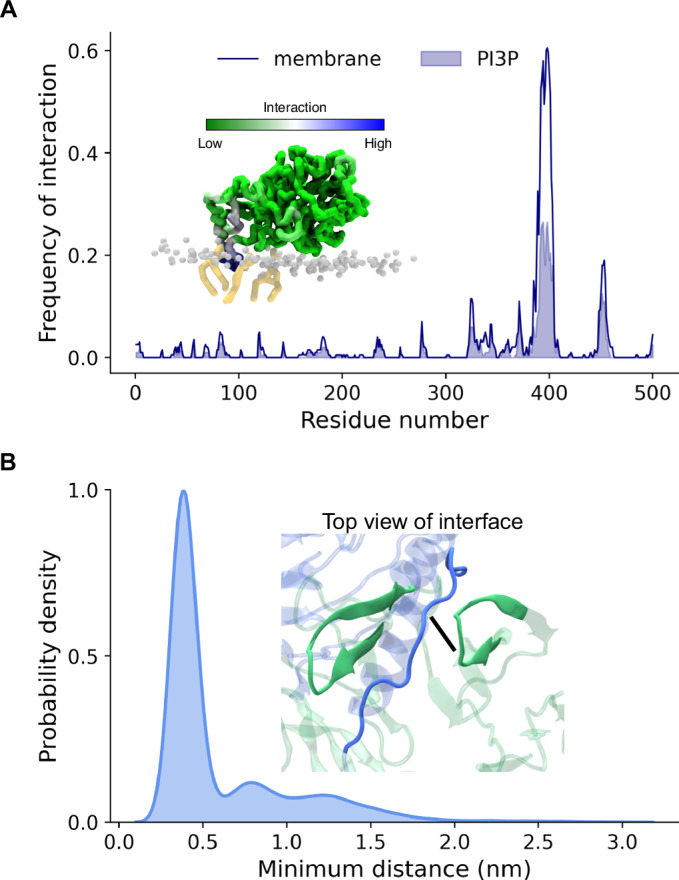
CG-MD analysis of Atg18 interaction with the membrane and its association with the Atg2 loop. (**A**) Atg18 spontaneously binds to a membrane primarily through interactions with PtdIns3P. Dark traces show the binding to membranes, while the lighter traces show interactions with PtdIns3P only. The inset shows the protein colored by its membrane interaction frequency. (**B**) Probability density of the interactions between Atg18 and Atg2 (residues 922–934) at their binding interfaces.

**Figure 8:**
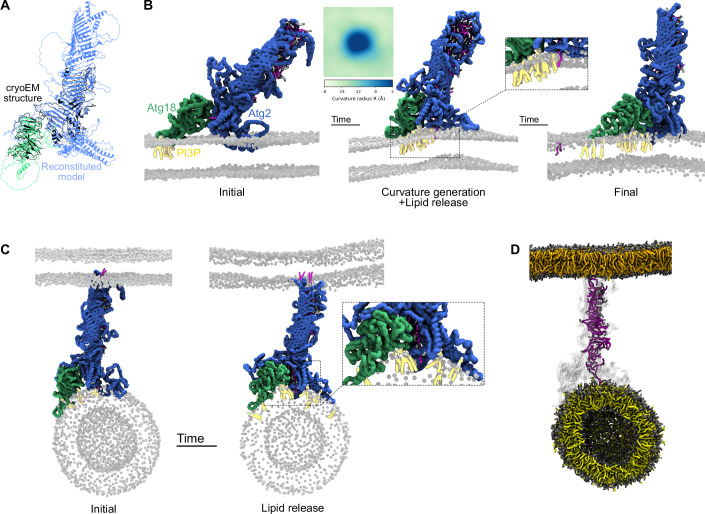
MD simulations reveal lipid-delivery capability of the resolved structure. (**A**) Cartoon representation of the reconstructed full-length model of the complex (Atg2 in light blue, Atg18 in light green) using the resolved portion as a scaffold (black). (**B**) Sample snapshots showing the initial setup of the lipid-filled complex on a flat bilayer and the subsequent release of one lipid into the bilayer. The inset shows a quantification of the membrane curvature generated by the complex. Atg18 is in green, Atg2 is in blue, the lipids inside the tunnel are shown in purple, the bilayer surface is shown by the grey PO4 beads, and the PtdIns3P (PI3P) lipids in the vicinity of Atg18 are shown in faint yellow. (**C**) Representative snapshots of the in-silico ER–vesicle contact site model, illustrating the initial configuration with the N-terminal region of Atg2 bound to the ER and the C-terminal Atg2–Atg18 complex bound to the vesicle, followed by lipid release. (**D**) Snapshot from the same system illustrating lipid continuity between the ER and the vesicle through the protein complex. Lipids inside the tunnel are shown in purple.

Our structural and biochemical analyses revealed a biologically relevant interaction interface between Atg2 and Atg18. However, it remains unclear whether the structure of the soluble complex represents a conformation compatible with the phagophore-ER MCS architecture. To address this question, we performed CG-MD simulations to evaluate the compatibility of the resolved complex structure with its lipid-transport function. We first assessed Atg18’s propensity to bind PtdIns3P-rich membranes, independently of Atg2. To do so, we placed Atg18 at a distance of at least 2 nm from the membrane and ran unbiased MD to determine membrane binding (Srinivasan et al, 2024). In the simulations, we observed that Atg18 was spontaneously binding to the flat bilayer, mainly through interactions with PtdIns3P via its FRRG motif (Fig. EV5A), as expected (Baskaran et al, 2012; Krick et al, 2012), as well as via an unstructured loop at residues 392 to 405. Next, we reconstituted the full-length model of the Atg2-Atg18 complex using the resolved portion as a scaffold (Fig. 8A). We set up a system where the Atg2-Atg18 complex is bound to a flat bilayer, mimicking the phagophore membrane (see “Methods”). In our simulations, the complex was stably binding the lipid bilayer, establishing frequent interactions with PtdIns3P lipids via Atg18 while the Atg2 tunnel was oriented towards the bilayer through an interaction between the C-terminal disordered motif of Atg2 (Atg2_C domain) and the membrane (Fig. 8B).

To further assess the role of the residues 922–934 in Atg2 at maintaining the interface with Atg18, we released the elastic network in this region, allowing the loop formed by these amino acids to freely sample other possible conformations in the CG simulations. Intriguingly, throughout the simulation, the loop remained within a 2 nm radius of Atg18, spending ~70% of the simulation time within 0.7 nm of the protein (Fig. EV5B). This result confirmed that the identified binding region in Atg2 is key for a stable, persistent interaction of Atg2 with Atg18. Interestingly, we also observed that the complex induces significant membrane curvature (Fig. 8B, inset), likely due to the geometrical arrangement of the two proteins in the complex, which is kept fixed by the elastic network in our CG model. The resulting curved bilayer is directly positioned below the opening of the Atg2 tube, potentially priming the process of lipid release. Indeed, during the simulations, we observe the release of one lipid from the Atg2 cavity into the membrane (Fig. 8B).

Based on the observed preference of the Atg2-Atg18 complex for membrane curvature, and considering that specific regions of the nascent phagophore are characterized by high membrane curvature (Mari et al, 2010; Yamamoto et al, 2012; Sawa-Makarska et al, 2020), we next opted to explicitly incorporate membrane curvature in our minimal model of a phagophore-ER MCS in the following way: At the C-terminus of Atg2, we modeled the interaction of the Atg2-Atg18 complex with a lipid vesicle of around 20 nm in diameter; at the N-terminus, we modeled the interaction of the N-terminal domain Atg2 with a flat lipid bilayer mimicking the ER (Fig. 8C). In the simulations, the protein complex remains stably bound with the two membranes, and, consistent with our previous observations, the interface with the lipid vesicle was primarily established via interactions between PtdIns3P and Atg18 and the C-terminus of Atg2 (Fig. 8C). Finally, also in this case, we observe lipid exchange at both ends, and the complex establishes lipid continuity between the two bilayers (Fig. 8D), further supporting our working model that our Atg2-Atg18 structure is compatible with a mechanism of bulk lipid transport via the Atg2 cavity from the ER to the isolation membrane of the nascent autophagosome.

## Discussion

Atg2 and Atg18 have a fundamental role as part of lipid transfer machinery that allows the rapid growth of the phagophore into an autophagosome. However, the precise interplay of both proteins and the regulation of the lipid transfer activity at the phagophore-ER MCS have remained unclear. Here, we solved the structure of the Atg2-Atg18 complex and characterized an interface between the amino acids 922–934 in Atg2 and the blades 2 and 3 of Atg18. These amino acid residues of Atg2 are required for Atg18 recruitment to the PAS, efficient Atg2-mediated lipid transfer, phagophore elongation and ultimately autophagy progression. These findings agree with and refine previous observations that describe the Atg2 binding site in Atg18 located in the loop 2 (residues 90–94, TFPTS, between blade 2 and 3) of its ß-propeller (Rieter et al, 2013), which we also found contacting Atg2 in our structural analysis (Figs. 2D,E and EV2B). This interface resembles previously reported WIR-peptide in WIPI3 (Ren et al, 2020), which is considered to act redundantly with WIPI4. Although the 922–934 region does not fully match with the proposed WIR-motif sequence, its hydrophobic nature seems to still promote the interaction.

Deletion of residues 922–934 in Atg2 impaired phagophore elongation (Fig. 4), resembling the phenotype observed in mutants that disrupt Atg2 activity (Kotani et al, 2018) or its interaction with Atg9 (Gómez-Sánchez et al, 2018). This is likely due to the specific impairment of the Atg2-Atg18 complex formation at the PAS, as the basal lipid transfer activity of Atg2 in vitro was not affected by the 922–934 truncation as well as Atg2 interaction with Atg9 (Fig. EV4A). However, Atg2^∆922–934^ was insensitive to the presence of Atg18 in vitro (Fig. 7). In contrast, the deletion of residues 1149–1157 in Atg2, which we also found to contact Atg18, did not impair autophagy (Fig. 3). Thus, the 1149–1157 amino stretch does not provide a functionally critical interface with Atg18 despite its proximity to the Atg18 residues known to be essential for binding with Atg2 (Watanabe et al, 2012). Our structural findings also agree with the recent work (Sakai et al, 2025) that predicted Atg2-Atg18 complex structure using AlphaFold3. The authors identified similar residues whose mutations likewise abolish autophagy and disrupt the complex formation. However, our structure of the Atg2-Atg18 complex appears to be slightly different from the AlphaFold3 prediction, in which Atg18 is located much closer to Atg2 (approximately by 5 Å). Likewise, the structure of the Atg2 fragment 922–934 and its position at the interface with blades 2 and 3 of Atg18 in the AlphaFold3 prediction differ from our cryo-EM data (Appendix Fig. S2B,C). Our cryo-EM structure of yeast Atg2-Atg18 shares overall similarities (Fig. EV2) with the human ATG2A–WIPI4 complex (Wang et al, 2025). However, a notable difference between the structures is the rotation and ~46° tilt of the WIPI4 position compared to the Atg18 position in our structure (Fig. EV2B), though the ATG2A loop with the corresponding residues 922–934 of yeast Atg2 is not solved in the ATG2A-WIPI4 structure. WIPI4 binds via its blade 2 to the C-terminal β-strands of ATG2A, similar to the interaction between Atg18 and Atg2 in yeast, however, employing a different set of residues for the interaction (Figs. 2G and EV2E). The yeast complex also requires the 922–934 region of Atg2 (Figs. 2F and EV2B), which we identified as an essential interface. Whether this structural variation reflects a functional state or is mitigated in the context of lipid bilayers and the lipid scramblase Atg9, remains to be determined.

Consistent with previous analysis in yeast and mammals (Zheng et al, 2017; Maeda et al, 2019; Valverde et al, 2019; Vliet et al, 2022), our structure reveals that Atg2 has a tube-like architecture with a central hydrophobic groove (cavity) that is laterally open to the cytosol (Fig. 2D,H,J). Our cryo-EM structure closely resembles the structure of *C. thermophilum* VPS13 (Li et al, 2020) and *C. elegans* LDP-3 (Kang et al, 2025), a protein involved in ER to plasma membrane lipid transfer. All three proteins are proposed to mediate bulk transfer of lipids between organelles (Kang et al, 2025; Reinisch et al, 2025). As we purified the Atg2-Atg18 complex from the cytosol, our data suggest that the entire central hydrophobic tunnel of Atg2 and likely also other BLTPs, may be permanently filled with lipids (Figs. 2H–J and 8D). This observation is consistent with previous reports showing lipids bound to purified ATG2A (Valverde et al, 2019), suggesting that the lipid loading occurs independently of Atg18 and may therefore contribute to stabilize the structure of the protein, especially when it is cytosolic. Atg2 activity as a lipid transfer protein is, however, strongly dependent on its interaction with Atg18, though the precise mechanism of Atg18-mediated activation remains speculative. Atg18 was initially proposed as PtdIns3P-dependent recruiter of Atg2 to the phagophore (Obara et al, 2008; Chowdhury et al, 2018; Kotani et al, 2018), and this was the main explanation of the stimulatory effect of Atg18 on Atg2’s lipid transfer activity. Although this Atg18-mediated recruitment may occur under certain experimental conditions, such as when membranes of low curvature are used in vitro assays (Osawa et al, 2019), our in vivo observations contrast with this hypothesis and support more recent findings showing that Atg2 is recruited to the PAS and can tether membranes independently of Atg18, yet the phagophore elongation remains blocked (Rieter et al, 2013; Gómez-Sánchez et al, 2018, 2025). Although our structural analysis does not clarify how Atg18 contributes to the lipid transfer, we speculate that the interaction with Atg18 is required to stimulate Atg2-mediated lipid transport through conformational rearrangements in the membrane-binding region. Our CG-MD simulations show that the arrangement of the complex on the membrane, mediated by PtdIns3P binding through Atg18 and the C-terminal of Atg2, is compatible with regions of the membrane with high curvature (Fig. 8C), reminiscent of small Atg9-vesicles or the extremity of the phagophore. Indeed, this conformation seems to be responsible of membrane perturbations (Fig. 8B) (Sakai et al, 2025), which in turn might facilitate the incorporation of lipid molecules into the acceptor membrane. In vivo, this might be even further regulated, as additional factors like Atg9, TRAPPIII and Ypt1 also play an essential role in the phagophore elongation (Gómez-Sánchez et al, 2025). Furthermore, Atg18 oligomerization, as observed in isolation, may contribute to higher-order MCS formation at the phagophore-ER interface (Maeda et al, 2019; Mann et al, 2023).

Our in vitro assays suggest that the purified proteins can interact and perform lipid transfer, and the efficiency of this process might result from a coordinated and synergic interplay of multiple factors, including the scramblase activity of the Atg9 trimers. All these regulations converge in efficient Atg2-mediated lipid transfer activity. Notably, our in vitro functional analyses, cryo-EM, structural modelling, and MD simulations collectively demonstrate that Atg2 forms a stable complex with Atg18 through a distinct set of residues. In this arrangement, Atg18 adopts an orientation that is favorable for its interactions with PtdIns3Ps in the membrane, while simultaneously positioning the Atg2 tunnel entry towards the membrane and allowing concurrent interaction of Atg2 with the Atg9 scramblase (Figs. 2, 8, EV3, and EV4C). How such a lipid transfer and scrambling machinery is coordinated and eventually turned off, belongs to the exciting questions that future studies must address.

## Methods

**Table:** Reagents and tools table

Reagent/resource	Reference or source	Identifier or catalog number
**Experimental models**		
BY4727 GAL1pr-Atg18-TAP (*Saccharomyces cerevisiae*)	Gómez-Sánchez et al, 2018	CU 9028
BY GALpr-Atg2 GALpr-Atg18-TAP (*Saccharomyces cerevisiae*)	Gómez-Sánchez et al, 2018	CU 9058
BY4741 GALpr-Atg2-TAP (*Saccharomyces cerevisiae*)	Gómez-Sánchez et al, 2018	CU 9030
BY 4727 GAL1pr-Atg9-3xFLAG (*Saccharomyces cerevisiae*)	Gómez-Sánchez et al, 2018	CU 10110
BY4742 pho8∆60 (*Saccharomyces cerevisiae*)	Reggiori´s lab	CU 10489
SEY 6210 atg2∆ ADHp-mCherry-Atg8 (*Saccharomyces cerevisiae*)	This study	CU 15120
SEY 6210 atg2∆ pRS404-ATG2pr-ATG2(∆922-934)-GFP ADHp-mCherry-Atg8 (*Saccharomyces cerevisiae*)	This study	CU 15121
SEY6210 atg2∆ pRS404-ATG2pr-ATG2(∆1149-1157)-GFP AHDp-mCherry-Atg8 (*Saccharomyces cerevisiae*)	This study	CU 15158
SEY6210 atg2∆ pRS404-ATG2pr-ATG2-GFP ADHp-mCherry-Atg8 (*Saccharomyces cerevisiae*)	This study	CU 15159
BY4742 pho8∆60 atg2∆ (*Saccharomyces cerevisiae*)	This study	CU 15300
BY4742 pho8∆60 atg2∆ pRS405-ATG2p- ATG2-GFP (*Saccharomyces cerevisiae*)	This study	CU 15429
BY4742 pho8∆60 atg2∆ pRS405-ATG2p- ATG2(∆922-934)-GFP (*Saccharomyces cerevisiae*)	This study	CU 15430
BY4742 pho8∆60 atg2∆ pRS405-ATG2p- ATG2(∆1149-1157)-GFP (*Saccharomyces cerevisiae*)	This study	CU 15432
SEY6210 atg2∆ pRS404-ATG2pr-ATG2(∆922-934)-GFP ADHp-mCherry-Atg8 Atg18-Halo (*Saccharomyces cerevisiae*)	This study	CU 15251
SEY6210 atg2 ∆ pRS404-ATG2pr-ATG2-GFP ADHp-mCherry-Atg8 Atg18-Halo (*Saccharomyces cerevisiae*)	This study	CU 15253
BY4741 atg2∆ pRS406-GAL1p-ATG2(∆922-934)-TAP (*Saccharomyces cerevisiae*)	This study	CU 15620
BY4732 GAL1p-ATG18-3xFLAG (*Saccharomyces cerevisiae*)	This study	CU 15287
BY4742 pho8∆60 atg2∆ pRS405-ATG2p- ATG2-GFP mCherry-Atg8 Atg18-Halo (*Saccharomyces cerevisiae*)	This study	CU 16286
BY4742 pho8∆60 mCherry-Atg8 (*Saccharomyces cerevisiae*)	This study	CU16316
BY4742 pho8∆60 Atg18-Halo (*Saccharomyces cerevisiae*)	This study	CU 16317
BY4742 pho8∆60 atg2∆ pRS405-ATG2prom-ATG2(D922-934) (*Saccharomyces cerevisiae*)	This study	CU 16292
BY4742 pho8∆60 atg2∆ pRS405-ATG2prom-ATG2 (*Saccharomyces cerevisiae*)	This study	CU 16293
BY4742 pho8∆60 atg2∆ pRS405-ATG2prom-ATG2(D1149-1157) (*Saccharomyces cerevisiae*)	This study	CU 16302
SEY6210 ATG18-13xmyc::TRP1 atg2Δ::HIS5S.p. pRS405-ATG2pr-ATG2-GFP (*Saccharomyces cerevisiae*)	This study	PVY111
SEY6210 ATG18-13xmyc::TRP1 atg2Δ::HIS5S.p. pRS405-ATG2pr-ATG2D922-934-GFP (*Saccharomyces cerevisiae*)	This study	PVY112
SEY6210 ATG18-13xmyc::TRP1 atg2Δ::HIS5S.p. pRS405::LEU2 (*Saccharomyces cerevisiae*)	Gómez-Sánchez et al, 2018	RSGY015
SEY6210 ATG9-GFP::TRP1 atg2Δ:HIS5S.p. pRS405::LEU2 (*Saccharomyces cerevisiae*)	Reggiori´s lab	RSGY006
SEY6210 ATG18-13xmyc::TRP1 atg2Δ::HIS5S.p. pATG2-TAP::LEU2 (*Saccharomyces cerevisiae*)	Gómez-Sánchez et al, 2018	RGSY012
SEY6210 ATG9-GFP::NatMX6 ATG18-13xMYC::HIS3MX6 atg2Δ::hphNT1 pRS404ATG2-TAP::TRP1 (*Saccharomyces cerevisiae*)	Reggiori´s lab	RGY590
SEY6210 ATG9-GFP::NatMX6 ATG18-13xMYC::HIS3MX6 atg2Δ::hphNT1 pRS405-ATG2(∆922-934)-TAP::LEU2 (*Saccharomyces cerevisiae*)	This study	PVY129
SEY6210 atg8Δ::loxP-kanMX-loxP pCumCheV5ATG8::URA3 SEC23-GFP::HIS3MX6 atg2Δ::hphNT1 pRS::TRP1 (*Saccharomyces cerevisiae*)	Reggiori´s lab	RGY541
SEY6210 atg8Δ::loxP-kanMX-loxP pCumCheV5ATG8::URA3 SEC23-GFP::HIS3MX6 atg2Δ::hphNT1 pATG2-TAP::TRP1 (*Saccharomyces cerevisiae*)	Reggiori´s lab	RGY637
SEY6210 atg8Δ::loxP-kanMX-loxP pCumCheV5ATG8::URA3 SEC23-GFP::HIS3MX6 atg2Δ::hphNT1 pRS405-ATG2(∆922-934)-TAP::LEU2 (*Saccharomyces cerevisiae*)	This study	PVY122
SEY6210 atg8Δ::loxP-kanMX-loxP pCumCheV5ATG8::URA3 SEC63-GFP::HIS3MX6 atg2Δ::hphNT1 trs85Δ::TRP1 LEU2::pRS405 (*Saccharomyces cerevisiae*)	This study	PVY118
SEY6210 atg8Δ::loxP-kanMX-loxP pCumCheV5ATG8::URA3 SEC63-GFP::HIS3MX6 atg2Δ::hphNT1 trs85Δ::TRP1 pRS405-ATG2-TAP::LEU2 (*Saccharomyces cerevisiae*)	This study	PVY123
SEY6210 atg8Δ::loxP-kanMX-loxP pCumCheV5ATG8::URA3 SEC63-GFP::HIS3MX6 atg2Δ::hphNT1 trs85Δ::TRP1 pRS405-ATG2(∆922-934)-TAP::LEU2 (*Saccharomyces cerevisiae*)	This study	PVY124
SEY6210 atg8Δ::loxP-kanMX-loxP pCumCheV5ATG8::URA3 pGFP-YPT1::TRP1 atg2Δ::hphNT1 pRS::LEU2 (*Saccharomyces cerevisiae*)	Gómez-Sánchez et al, 2025	RGY1015
SEY6210 atg8Δ::loxP-kanMX-loxP pCumCheV5ATG8::URA3 pGFP-YPT1::TRP1 atg2Δ::hphNT1 pRS405-ATG2-TAP::LEU2 (*Saccharomyces cerevisiae*)	This study	PVY125
SEY6210 atg8Δ::loxP-kanMX-loxP pCumCheV5ATG8::URA3 pGFP-YPT1::TRP1 atg2Δ::hphNT1 pRS405-ATG2(∆922-934)-TAP::LEU2 (*Saccharomyces cerevisiae*)	This study	PVY126
SEY6210 atg8Δ::loxP-kanMX-loxP pCumCheV5ATG8::URA3 TRS85-mNeonGreen-3xHA::TRP1 atg2Δ::hphNT1 pRS::LEU2 (*Saccharomyces cerevisiae*)	Gómez-Sánchez et al, 2025	RGY1024
SEY6210 atg8Δ::loxP-kanMX-loxP pCumCheV5ATG8::URA3 TRS85-mNeonGreen-3xHA::TRP1 atg2Δ::hphNT1 pRS405-ATG2-TAP::LEU2 (*Saccharomyces cerevisiae*)	This study	PVY127
SEY6210 atg8Δ::loxP-kanMX-loxP pCumCheV5ATG8::URA3 TRS85-mNeonGreen-3xHA::TRP1 atg2Δ::hphNT1 pRS405-ATG2(∆922-934)-TAP::LEU2 (*Saccharomyces cerevisiae*)	This study	PVY128
**Recombinant DNA**		
pRS315 CUP1pr-BFP-Ape1	Pfaffenwimmer et al, 2014	4202
pRS404-ATG2pr-ATG2-GFP	Reggiori´s lab	5285
pRS404-ATG2pr-ATG2(∆21)-GFP	Reggiori´s lab	5289
pRS404-ATG2pr-ATG2(∆922-934)-GFP	This study	6080
pRS404-ATG2pr-ATG2(∆993-999)-GFP	This study	6081
pRS404-ATG2pr-ATG2(∆1124-1157)-GFP	This study	6082
pRS405-ATG2p- ATG2-GFP	This study	6142
pRS405-ATG2p- ATG2(D922-934)-GFP	This study	6143
pRS405-ATG2p- ATG2(D993-999)-GFP	This study	6144
pRS405-ATG2p- ATG2(D1149-1157)-GFP	This study	6145
pRS406-GAL1p-ATG2(D922-934)-TAP	This study	6155
pKS133-6 pFA6a-hphNT1	Janke et al, 2004	741
pYM-N9 ADHp-mCherry	Ungermann lab	3338
pYM26 (mGFP) MET15	Ungermann lab	3883
pYM25 HALO-tag	Ungermann lab	3601
pYM26 (3xFLAG) MET15	Ungermann lab	3588
pYM mCherry-FYVE (EEA1) hphNT1	This study	6030
pYM-N Gal1pr-TAP	Ungermann lab	5623
pYM-N23 (GAL1pr)	Janke et al, 2004	1097
pRS405-ATG2prom-ATG2(D922-934)	This study	6520
pRS405-ATG2prom-ATG2	This study	6526
pRS405-ATG2prom-ATG2(D1149-1157)	This study	6528
pRS405-ATG2prom-ATG2(D922-934)-TAP	This study	pl-PV23
**Antibodies**		
Anti-FLAG M2	Sigma-Aldrich	Cat. #F1804
Anti-TAP	Invitrogen	Cat. #CAB1001
Anti-mouse DyLight800	Invitrogen	Cat. #SA5-35521
Anti-rabbit DyLight680	Invitrogen	Cat. #35568
Goat anti-rabbit, HRP-conjugated	Cell Signaling	Cat. #7074S
Rabbit anti-mouse, HRP-conjugated	Sigma-Aldrich	Cat. #A9044
Anti-GFP	Clontech	cat# 632381
Anti-Myc	Santa Cruz Biotechnology	cat# SC-40
Anti-Vac8	Veit et al, 2001	
Anti-Pgk1	Sánchez-Wandelmer et al, 2017	
**Oligonucleotides and other sequence-based reagents**		
PCR primers	This study	Table EV1
**Chemicals, enzymes, and other reagents**		
Protease inhibitor Mix FY	SERVA	cat# 39104.01
PMSF	Sigma-Aldrich	cat# P7626
cOmplete Mini Protease Inhibitor Cocktail	Roche	cat# 11836153001
IgG Sepharose 6 Fast Flow	Cytiva	cat# 17096901
Ni-NTA agarose	QIAGEN	cat# 30210
Triton X-100	Sigma-Aldrich	cat# 9036-19-5
n-dodecyl β-D-maltoside	Carl Roth GmbH	CN26.4
anti-FLAG M2 affinity gel	Sigma-Aldrich	A2220
dioleoyl-phosphatidylcholine (DOPC)	Avanti Polar Lipids	cat# 850375
dioleoyl-PE (DOPE)	Avanti Polar Lipids	cat# 850725
dioleoyl-phosphatidylserine (DOPS)	Avanti Polar Lipids	cat# 840035
N-(7-nitrobenz-2-oxa-1,3-Diazol-4-yl)-1,2-dihexadecanoyl-sn-glycero-3-phosphoethanolamine (NBP-PE	Invitrogen	cat# N360
Egg Liss Rhodamine-PE	Avanti Polar Lipids	cat# 810146
PtdIns3P	Echelon Biosciences	cat# P-3016
4-Nitrophenyl phosphate di(tris) Salt	Sigma	N3254
CMAC	Thermo Fisher Scientific	C2110
JFX650 HaloTag Ligand	Levis Lab	
GFP-Trap_A agarose	ChromoTek, gta	
**Software**		
GraphPad Prism 10	https://www.graphpad.com	
AcquireMP software	Refeyn Ltd	
DiscoverMP v2023	Refeyn Ltd	
cryoSPARC v3 and v4	Punjani et al, 2017	
UCSF ChimeraX	Meng et al, 2023	
Fiji	Schindelin et al, 2012	
Huygens Essential	https://svi.nl/Huygens-Essential	
SWISS-MODEL	Waterhouse et al, 2018	
AlphaFold3	Abramson et al, 2024	
GROMACS package (v 2023.3)	https://www.gromacs.org/	
Matplotlib	10.1109/MCSE.2007.55	
MDAnalysis	Michaud-Agrawal et al, 2011	
**Other**		
Freezer Mill 6875D	SPEX samplePrep	
Superose 6 increase 5/150 column	Cytiva	
SpectraMax M3 Microplate reader	Molecular Devices LLC	
Soda Lime Glass Beads	Thermo Scientific	cat# 5663R5O

### Yeast strains

Yeast strains used in this study are listed in the Reagents and Tools Table. Deletion of *ATG2* in was performed by replacement of the open reading frame (ORF) with the hygromycin resistance gene (hphNT1) or the S.p. *HIS5* gene, both generated by polymerase chain reaction (PCR) with primers (see Table EV1) containing 68 bases identical to the flanking regions of the ORF (Janke et al, 2004). The gene knockout was confirmed by PCR.

Strains expressing GFP-tagged and TAP-tagged Atg2, Atg2^∆922-934^ or Atg2^∆1149-1157^ were generated by transformation *atg2Δ* knockouts with integrative plasmids carrying the endogenous *ATG2* promoter followed by the ORF and GFP or TAP, respectively. The strain overexpressing TAP-tagged Atg2^∆922-934^ was generated by transforming an *atg2∆* strain with the integrative plasmid pRS406-*GAL1*p-*ATG2(Δ922-934)-TAP* (see Reagents and Tools Table).

Strains expressing mCherry-Atg8 or Atg18-Halo were generated by PCR-based integration (Janke et al, 2004). The PCR products containing the sequences of the fluorescent proteins, HaloTag or mCherry, were generated using the corresponding plasmids as templates (see Reagents and Tools Table) and primers containing 60 bases identical to the flanking N-terminus or C-terminus of the tagged genes (see Table EV1).

### Plasmids

The plasmids used in this study are listed in Reagents and Tools Table. Plasmids carrying the GFP-tagged Atg2^∆922-934^ or Atg2^∆1149-1157^ variants were generated by Quick Change mutagenesis using primers annealing with the flanking regions of the deletion sequences, and the plasmids pRS404-*ATG2pr-ATG2-GFP* or pRS405-*ATG2pr-ATG2-GFP* as template.

### Protein purification from yeast

Strains overexpressing TAP (Tandem Affinity Protein: protein A-TEV cleavage site- calmodulin binding protein; CBP) tagged versions of Atg2, Atg18 or the Atg2-Atg18 complex under the control of the *GAL1* promoter were grown in YPG medium (1% yeast extract, 2% peptone, 2% galactose) at 30 °C until OD_600_ 2.5-3. Yeast was harvested by centrifugation and the cell pellets resuspended in lysis buffer (50 mM Tris/HCl, pH 8, 500 mM NaCl, 1.5 mM MgCl_2_) containing 1 mM phenylmethylsulfonyl fluoride (PMSF) and 1× protease inhibitor Mix FY (SERVA, cat# 39104.01) in a ratio of 1 g pellet/1 ml buffer. Resuspended cells were frozen in liquid nitrogen as drops. The lysis was performed using a Freezer Mill 6875D (SPEX samplePrep) and a program of 10 cycles of 2 min pulse at 12 counts per second. Lysates were clarified by centrifugation at 3200× g for 10 min at 4 °C, and subsequently centrifuged at 100,000 *g* for 45 min at 4 °C. Supernatants were incubated with IgG Sepharose 6 Fast Flow (Cytiva, cat# 17096901) according to the manufacturer's specifications. Proteins were then eluted by incubation with a self-purified TEV (Tobacco Etch Virus)-6xHis protease for 60 min at 4 °C. The TEV-protease was removed by incubation of the eluate with Ni-NTA agarose (QIAGEN, cat# 30210) for 30 min at 4 °C. For the Atg18-Atg2 complex purification, eluates were concentrated with a 100 kDa cutoff Amicon ultra centrifugal filter (Millipore, cat# UFC5100), and the complex was separated by size exclusion chromatography in a Superose 6 increase 5/150 column (Cytiva).

Atg9 was purified from a strain overexpressing the protein fused to 3xFLAG under the control of the *GAL1* promoter, as described previously (Chumpen Ramirez et al, 2023). Briefly, cells were lysed in the TBM250 buffer (50 mM Tris/HCl, pH 7.5, 500 mM NaCl, 1.5 mM MgCl_2_) plus 1X protease inhibitor Mix FY and 1 mM PMSF. Lysates were clarified by centrifugation at 3200× *g* for 5 min at 4 °C, followed by ultracentrifugation at 100,000× *g* for 90 min at 4 °C. The pellet fraction (membranes) was resuspended in the TBM250 buffer containing 2% n-dodecyl β-D-maltoside (DDM; Carl Roth GmbH, CN26.4) and incubated for 1 h at 4 °C for solubilization. Solubilized membranes were ultracentrifuged at 100,000× *g* for 45 min at 4 °C, and the supernatant fraction was diluted with the TBM250 buffer up to a final DDM concentration of 0.1%. The sample was incubated with anti-FLAG M2 affinity gel (Sigma-Aldrich, A2220), according to the manufacturer's specifications. Atg9-3xFLAG was eluted with the TBM250 buffer containing 0.1% DDM and excess of FLAG peptide (0.5 mg/ml). All proteins were frozen in liquid nitrogen and stored at −80 °C until use.

### Mass photometry analysis

A Refeyn TwoMP (Refeyn Ltd) was used for mass photometry experiments. Data were recorded using AcquireMP software and analyzed using DiscoverMP v2023 (both Refeyn Ltd). Purified protein samples at a final concentration of 10 nM in a total volume of 20 μl were applied to High Precision Cover Glasses (Marienfeld) using perforated silicone gaskets that were placed on top of the cover glasses, to form wells for measuring the samples. Mass calibration was carried out using β-amylase (Carl Roth).

### In vitro lipid transfer assay

Liposome preparation was performed as described (Chumpen Ramirez et al, 2023). Briefly, lipids (Avanti Polar Lipids) were mixed in a composition of 46 mol% dioleoyl-phosphatidylcholine (DOPC) (cat# 850375), 30 mol% dioleoyl-PE (DOPE) (cat# 850725), 20 mol% dioleoyl-phosphatidylserine (DOPS) (cat# 840035), 2 mol% N-(7-nitrobenz-2-oxa-1,3-Diazol-4-yl)-1,2-dihexadecanoyl-sn-glycero-3-phosphoethanolamine (NBP-PE) (Invitrogen, cat# N360), 2 mol% Egg Liss Rhodamine-PE (cat# 810146), for donor vesicles, and 50% DOPC, 25% DOPE, 20% DOPS, 5% PtdIns3P (Echelon Biosciences, cat# P-3016) for acceptor vesicles. The lipid mixes were dried under vacuum, resuspended in buffer (20 mM HEPES/NaOH, pH 8, 150 mM NaCl) to a final concentration of 4 mM, and freeze-thawed 10 times. Donor and acceptor vesicles were extruded through Nucleopore Track Filter Membranes with 200 nm, 100 nm and 50 nm pore size (Whatman, 800281, 800309, 800308). Donor (D) and acceptor (A) liposomes were mixed in a final concentration 0.2 mM and 0.6 mM, respectively, in a 25 μl total volume. Atg2 was added at the concentrations indicated in the figure legends, immediately before the measurement of the NBD signal. The NBD fluorescence intensity (FI) was recorded in a SpectraMax M3 Microplate reader (Molecular Devices LLC), with an excitation wavelength of 460 nm and an emission wavelength of 535 nm, at 30 °C at 1 min intervals for 60 or 90 min. The NBD FI measured at each time point was normalized to the FI at time = 0 (FI0). All the experiments were performed in technical duplicates and independently repeated three times. The statistical analyses were performed using the GraphPad Prism 10 software, using two-way ANOVA test with Tuckey post-test for correction of multiple comparison and a 95% confidence interval (*P* value < 0.05).

### Cryo-EM sample preparation and data acquisition

In all, 3 µl of purified Atg2-Atg18 complex at a protein concentration of approximately 1.5 mg/ml was applied onto double-glow-discharged C-Flat grids (R1.2/1.3 3Cu-50) (EMS) and immediately plunge-frozen in liquid ethane using a Vitrobot Mark IV (Thermo Fisher Scientific), with the environmental chamber set at 100% humidity and 4 °C. A Glacios cryogenic transmission electron microscope (Thermo Fisher Scientific) operating at 200 kV with a Selectris imaging energy filter. A Falcon 4i direct electron detector (both Thermo Fisher Scientific) was used for automatic data acquisition (7220 movies) (Fig. EV1G). Data collection was performed in Electron Event Representation mode at a nominal magnification of 130,000× (0.92 Å per pixel) in the defocus range of −0.8 to −1.8 μm, with an exposure time of approximately 5.2 s, resulting in a total electron dose of 50 e- Å^−2^.

### Cryo-EM data processing

Cryo-EM data processing was carried out using the cryoSPARC v3 and v4 software (Punjani et al, 2017). Motion correction and contrast transfer function (CTF) estimation was followed by extensive particle picking using blob, template and neural network-based Topaz (Bepler et al, 2019) picking procedures. The picked particles were classified and cleaned using multiple rounds of 2D classification (using Fourier-cropped box), with intermediate duplicate particle removal. Afterwards, ~260,000 particles were selected and used in a round of ab-initio 3D reconstruction with three classes followed by heterogeneous refinement with three classes. The particles from class 0 and 1 (~169,600), as well as the volume from class 1 were further used in a round of NU refinement (Punjani et al, 2020) reaching 4.05 Å resolution. Particles from this NU refinement job was further applied for training of additional rounds of Topaz picker, which finally resulted in ~868,500 newly picked particles (after duplicate removal). These particles were additionally cleaned by 2D classification and subjected to an ab-initio reconstruction with one class followed by an NU refinement achieving 3.99 Å resolution. The particles from this refinement were extracted using the full box size of 512 pixels (0.924 Å/pix) and further cleaned by a round of 2D classification, followed by a round of ab-initio reconstruction and heterogeneous refinement (both with three classes). The best class of the last heterogeneous refinement was finally refined in an NU refinement round providing a map at 4.01 Å resolution (Fig. EV1G).

### Model building and refinement

AlphaFold3 (Abramson et al, 2024) was used to generate initial molecular models of the Atg2-Atg18 complex (Uniprot IDs: P53855 and P43601). Initial models of Atg2 and Atg18 were manually fitted into the final NU refinement map. N-terminal region of Atg2 (1–514), as well as its fragments 557–560, 593, 604–643, 660, 665–666, 683–684, 700–704, 717, 730, 753–780, 816–818, 876, 910–913, 936–1023, 1057–1120, 1146, 1177–1182, 1190, 1279–1304, 1335–1592 were removed from the final model due to the poor resolution achieved in these areas of the map. Similarly, fragments 154–223, 234, 245–246, 289–290, 295, 312, 315, 321–411, 421–423, 438–439, 448–461, 500 were deleted from the model of Atg18. Furthermore, the Atg2 fragment 542–730 and the Atg18 fragment 296–463 were substituted with alanine residues in the model. The model was manually refined in Coot (Emsley and Cowtan, 2004) and further adjusted by iterative rounds of Real Space Refinement in Phenix (Liebschner et al, 2019), followed by manual modifications in Coot. Validation of the models was performed using MolProbity (Williams et al, 2018) in Phenix (see Table EV2). Models and cryo-EM maps were visualized in UCSF ChimeraX (Meng et al, 2023) and structural figures were prepared in Affinity Designer 2.

### Pho8Δ60 assay

Cells were grown in YPD medium (1% yeast extract, 2% peptone, 2% glucose) at 30 °C until OD_600_ 0.6–0.8 and then shifted to synthetic starvation medium (SD-N) (0.17% Yeast nitrogen base without ammonium sulfate and amino acids, 2% glucose, pH 5.5) to induce autophagy or YPD medium as a control for nutrient-rich condition, for 3 h at 30 °C. Three OD_600_ cell equivalents were harvested and resuspended in 400 μl of ice-cold lysis buffer (20 mM PIPES/KOH, pH 6.8, 0.5% Triton X-100, 50 mM KCl, 100 mM KAc, 10 mM MgCl_2_, 10 μM ZnSO_4_) supplemented with 1 mM PMSF. Cells were lysed with glass beads (0.25–0.5 mm diameter, Roth cat# A553.1) using a Disruptor Genie (Scientific Industries) and four cycles of 2 min of pulse and 5 min breaks on ice. Lysates were centrifuged at 13,000× *g* for 5 min at 4 °C, and cleared supernatants were collected and diluted with lysis buffer to a protein concentration of 0.4 μg/μl. Phosphatase activity was assessed by incubation of 100 μl of lysate with 400 μl of ALP buffer (250 mM Tris/HCl, pH 8.5, 0.4% Triton X-100, 50 mM KCl, 10 mM MgCl_2_, 10 μM ZnSO_4_, 1.25 mM p-nitrophenyl phosphate) at 37 °C for 20 min, followed by addition of 500 μl of 1 M glycine, pH 11, to stop the reaction. The absorbance of the p-nitrophenol dephosphorylation product was measured at 400 nm, and the concentration of p-nitrophenol was calculated using the Beer-Lamber law and the extinction coefficient (400 nm, pH 11) = 18,500 M^−1^ cm^−1^. The phosphatase activity was defined in arbitrary units (A.U.) as pmol of p-nitrophenol produced per min per μg of protein. The experiment was performed in technical duplicates and in three biological replicates. The statistical analysis was performed using GraphPad Prims 10, and a two-way ANOVA test with a Tuckey post-test and 95% confidence interval. *P* value significance levels: 0.1234 (ns), 0.0332 (*), 0.0021 (**), 0.0002 (***),  < 0.0001 (****).

### Fluorescence microscopy analysis

Fluorescence microscopy was performed with cells grown in YPD medium until early log phase (OD_600_ 0.2) or upon incubation in SD-N medium for 3 h to induce autophagy. Where indicated, vacuoles were stained with 1 μM CellTracker™ Blue 7-amino-4-chloromethylcoumarin dye (CMAC; Thermo Fisher Scientific, C2110) for 10 min at 30 °C (Stefan and Blumer, 1999) prior to imaging. To visualize Atg18-Halo, cells were incubated with 2.5 μM JFX650 HaloTag ligand (Levis Lab) for 30 min at 30 °C before imaging. Images were collected as Z-stack of six pictures with focal planes of 0.25 μm. For the giant Ape1 assay, Ape1 oligomers formation was induced in cells carrying the *pRS315-[CUP1pr-APE1]-[APEIpr-BFP-APEI]* plasmid by addition of 250 μM of CuSO_4_ to the synthetic complete medium (SC, 0.67% yeast nitrogen base, 2% glucose, amino acids and vitamins as needed). Cells were grown until OD_600_ 0.2 and then shifted to SD-N medium for 2 h to induce autophagy. The images were collected as Z-stack of 6 pictures with focal planes 0.25 μm. Image acquisition was done with a DeltaVision Elite microscope system (GE Healthcare, Applied Precision), equipped with a UAPON 100X TIRF oil/1.49 NA objective, a pco.edge 4.2 sCMOS camera (PCO) and a seven-color InsightSSI solid-state illumination system (GE Healthcare, Applied Precision). The analysis and quantification of the images was performed using the software Fiji (Schindelin et al, 2012) and when indicated, deconvolution of the images was performed using the Huygens Essential software. Statistical analyses were performed using the GraphPad Prism 10 software, and are indicated in the corresponding figure legends. *P* value significance levels: 0.1234 (ns), 0.0332 (*), 0.0021 (**), 0.0002 (***),  < 0.0001 (****).

For the analysis of the Ypt1 and Trs85 recruitment to the PAS, PAS-ER and Pas-ERES association, cells grown in YPD medium until log phase (OD600 ~ 0.7) and then transferred to SD-N medium for 1 h to induce autophagy. Vacuoles were stained with 1 μM CellTracker™ Blue 7-amino-4-chloromethylcoumarin dye (CMAC; Thermo Fisher Scientific, C2110) for 10 min at 30 °C before imaging (Stefan and Blumer, 1999). Images were collected as Z-stack of five pictures with focal planes of 0.25 μm using a Oxford Nanoimager microscope system (ONI, Oxford, United Kingdom) equipped with standard Olympus UPLXAPO100 lens with 1.518 inmersion oil (Hamamatsu Phtonics, Shizuoka, Japan) and Hamamatsu ORCA-Flash4.0 V3 Digital CMOS camera. The analysis, quantification and preparation of the images was carried out using the Fiji software. Statistical analyses were performed using the GraphPad Prism 10 software, and are indicated in the corresponding figure legends. *P* value significance levels: 0.1234 (ns), 0.0332 (*), 0.0021 (**), 0.0002 (***),  < 0.0001 (****).

### In vitro pull-downs

Yeast cells overexpressing Atg18–3×FLAG under the control of the *GAL1* promoter were grown in YPG medium to an optical density OD₆₀₀ 5. Cells were harvested, resuspended in lysis buffer (50 mM Tris-HCl, pH 7.4, 500 mM NaCl, 1.5 mM MgCl₂, 1 mM PMSF, FY protease inhibitor mix, and 1 mM DTT), and lysed using a Freezer-Mill. As a negative control, beads were incubated with lysates from cells not carrying Atg18–3×FLAG, supplemented with 1 μg/ml 3xFLAG peptide to reduce unspecific binding to the anti-FLAG paratope (LaCava et al, 2016). The lysates were clarified as described for protein purification and incubated with anti-FLAG M2 affinity gel (Sigma-Aldrich, Cat. #A2220) for 3 h at 4 °C with gentle rotation. Beads loaded with Atg18 or control beads were washed with 10 bed volumes of the same buffer and divided into 50 µl aliquots. Each aliquot was incubated with 3.5 µg of purified Atg2–CBP, Atg2^∆922-934^–CBP or buffer alone, at 4 °C for 1 h with mild agitation. After incubation, the flow-through was removed, and the beads were washed with 10 bed volumes of lysis buffer. Proteins were eluted with 0.1 M glycine-HCl (pH 3.5), prepared for SDS–PAGE, and resolved on 7.5% polyacrylamide gels. Western blotting was performed using anti-FLAG M2 (Sigma-Aldrich, Cat. #F1804) and anti-TAP (Invitrogen, Cat. #CAB1001) primary antibodies, followed by anti-mouse DyLight800- or anti-rabbit DyLight680-conjugated secondary antibodies (Invitrogen, Cat. #SA5-35521 and #35568, respectively). For quantification of Atg2/Atg2^∆922-934^ binding to Atg18, signal intensities were measured using ImageJ. The Atg18 signal was standardized across samples and used to normalize the co-eluted Atg2 or Atg2^∆922-934^ signal. The total amount of co-eluted protein was calculated based on the input signal intensity and the loading percentages in the gel. Statistical analysis was performed using the GraphPad Prism 10 software.

### In vivo co-immunoprecipitations

Yeast was grown at 30 °C to log phase in YPD. Cell equivalents of 300 OD_600_ were transferred to SD-N for 1 h, collected by centrifugation and resuspended in 1.6 ml of lysis buffer (20 mM Tris-HCl pH 8.0, 150 mM KCl, 5 mM MgCl_2_, containing 1% Triton X-100 (Sigma-Aldrich, cat# 9036-19-5), supplemented with 1 mM PMSF and the cOmplete Mini Protease Inhibitor Cocktail (Roche, cat# 11836153001). Cells were lysed by vortexing with glass beads (Soda Lime Glass Beads, 0.4–0.6 mm; Thermo Scientific, cat# 5663R5O) for 5 min at 4 °C, and lysates were cleared by centrifugation at 20,000×*g* for 10 min at 4 °C. Supernatants were incubated with 20 µl of pre-washed GFP-Trap_A agarose (ChromoTek, gta) with mild rotation for 2 h at 4 °C. Beads were washed five times with 1 ml lysis buffer. Protein complexes were eluted by resuspension in 60 µl of Laemmli sample buffer and boiling for 5 min at 95 °C. Proteins were finally separated by SDS–PAGE and examined by western blotting using anti-GFP (Clontech, cat# 632381), anti-Myc (Santa Cruz Biotechnology, cat# SC-40) and anti-Pgk1 (Sánchez-Wandelmer et al, 2017). The signal intensities of Atg2 and Atg18 were quantified in ImageJ and normalized to the loading control Pgk1. The signal of co-immunoprecipitated Atg18 was divided by the signal of the corresponding Atg2 variant in the elution fraction (IP).

For Atg9-GFP pull down experiments, the cells were grown as before and samples processed the same as for the Atg2 pull-down, except that the protein complexes were eluted by resuspension in 50 μl of Laemmli sample buffer and boiling for 5 min at 95 °C. Proteins were finally separated by SDS–PAGE and examined by western blotting using anti-GFP (Clontech, cat# 632381), anti-TAP (Thermo Scientific, CAB1001) and anti-Vac8 (Veit et al, 2001). Signal intensities of Atg2 and Atg9 were quantified using the ImageJ software. Interaction of Atg2 with Atg9 in the co-immunoprecipitation experiments was quantified by dividing the intensity of Atg2 bands by that of Atg9 bands, and expressing the obtained values relative to WT control. Statistical analyses were performed using the GraphPad Prism 10 software, and are indicated in the corresponding figure legends. *P* value significance levels: 0.1234 (ns), 0.0332 (*), 0.0021 (**), 0.0002 (***),  < 0.0001 (****).

### Molecular dynamics simulations

#### System setup

To reconstitute the full-length Atg2-Atg18 complex, the cryo-EM-resolved C-terminal portion of the structure was used as a scaffold. Missing regions within this portion were initially modeled using the SWISS-MODEL software (Waterhouse et al, 2018). The unresolved N-terminal portion of Atg2 was incorporated from the full-length AlphaFold3 (Abramson et al, 2024) prediction of Atg2. The full complex was then minimized in vacuum using the CHARMM36m force field (Huang et al, 2017). The minimized complex was then converted to CG using the Martinize script (Kroon et al, 2025).

The insane script was used to generate the bilayers (Wassenaar et al, 2015). To model the interaction of the Atg2-Atg18 complex with a lipid bilayer, OPM (Lomize et al, 2012) was initially used to determine the orientation of the complex relative to the membrane. The membrane was composed of DOPC:DOPS:PI3P in a ratio of 65:20:15. The box size was of 40 × 40 × 50 nm in the *x*, *y*, and *z* direction, respectively. Finally, the system was solvated and ionized with 0.15 M NaCl. To setup the Atg18-membrane system, Atg18 was taken from the reconstructed model and placed 2 nm away from the bilayer. The bilayer was composed DOPC:DOPS:PI3P in a ratio of 75:20:5. The box size was 22 × 22 × 28 nm in the *x*, *y*, and *z* direction, respectively. The system was solvated and ionized with 0.15 M of NaCl. To create the lipid vesicle, Bumpy (Boyd and May, 2018) was used to convert the CG membrane into a spherical vesicle with a diameter of 20 nm. The vesicle was symmetrically composed of DOPC:DOPS:PI3P in a ratio of 75:20:5. A total of six pores, 1 nm in diameter, were introduced into the vesicle, with two opposing pores along each dimension, to allow uninterrupted water and ion exchange and ensure sufficient equilibration. All pores were eventually sealed after sequential equilibrations. The vesicle was then solvated and ionized with 0.15 M NaCl. After sufficient equilibration of the vesicle alone, the lipid-filled Atg2-Atg18 complex was then positioned on the vesicle through its C-terminal following the OPM orientation. The new box was re-solvated and ionized. Next, to model the ER-phagophore contact site, the vesicle-bound, lipid-filled complex was placed in a box with a pre-equilibrated DOPC bilayer bound to the N-terminal of Atg2.

#### Simulation details

All simulations were performed with the GROMACS package (v 2023.3) (Spoel et al, 2005) using the Martini 3 force field (Souza et al, 2021). To maintain the secondary structure of the protein, an elastic network with a force constant of 700 kJ mol^−1^ nm^−2^ was applied, excluding the disordered regions. The upper and lower elastic bond cutoffs were 0.9 and 0.5 nm, respectively. Two CG models of the complex were created; one where the interface between both proteins in the complex was conserved by the elastic network and another where the elastic network of the Atg2 loop (residues 922–934) was released. The Atg2 tunnel was then filled with DOPC using a recently published protocol to fill cavities of lipid-transfer proteins (Srinivasan et al, 2024). To oversaturate the cavity, we adapted a recently-proposed in-silico lipid synthesis protocol (Nieto et al, 2023), which consists of the following iterative steps: 1. duplicating one of the lipids inside the cavity, 2. slow-growth simulation of the duplicated lipid to avoid clashes, and 3. Standard equilibration + production for 50 ns. The protocol was followed iteratively until 42 lipids were inside the cavity.

All systems were initially minimized using a steepest descent algorithm followed by a series of 6 equilibrations in NPT ensemble. Each equilibration had reduced restraints on the protein backbone and lipid heads until all restraints were removed in the last equilibration step. To equilibrate the vesicle, a flat-bottom restraint potential (Biriukov and Javanainen, 2023) was used to keep the lipid tails away from the pores during equilibration. For the production runs, all systems were kept at 310 K using a velocity-rescale thermostat (PMID: 17212484) that separately coupled the protein, membrane/vesicle, and solvent. A semi-isotropic Parrinello–Rahman barostat (Parrinello and Rahman, 1981) was used to maintain the pressure constant at 1 bar. A Verlet scheme with a buffer tolerance of 0.005 was used to calculate the nonbonded interactions. Reaction-field electrostatics was used to compute Coulombic interactions. A cutoff scheme was also applied to the van der Waals interactions. Both interactions employed a 1.1 nm cutoff and followed the Verlet cutoff scheme with a potential shift. A time step of 20 fs was used with the MD integrator. Two independent replicas of 6 µs each were simulated for all systems, except the ER-phagophore contact site systems which were simulated for 3 µs.

#### Simulation analysis

The frequency of interaction between Atg18 and the membrane was generated using gmx select to compute the percentage of the simulation time during which the protein was within 0.7 nm of the membrane or PI3P only. To measure the minimum distance between Atg2 residues 922–934 and its Atg18 interface, gmx mindist was used. The mean curvature of the membrane throughout the simulations was calculated using the MembraneCurvature modules from MDAnalysis (Michaud-Agrawal et al, 2011). All graphical representations were plotted using Matplotlib (Hunter, 2007). All visual representations were created using VMD (Humphrey et al, 1996) or ChimeraX (Pettersen et al, 2020).

## Supplementary information

Appendix

Table EV1

Table EV2

Peer Review File

Source data Fig. 1

Source data Fig. 2

Source data Fig. 3

Source data Fig. 4

Source data Fig. 5

Source data Fig. 6

Source data Fig. 7

Expanded View Figures

## Data Availability

Atomic coordinates have been deposited in the PDB: Atg2-Atg18 complex from yeast (PDB ID 9T0C). The associated cryo-EM density map has been deposited in the Electron Microscopy Data Bank (EMDB): Atg2-Atg18 complex from yeast (EMD ID EMD-55395). MD simulation data are deposited in Zenodo: 10.5281/zenodo.18824109 (10.5281/zenodo.18824109). Fluorescence microscopy data corresponding to Fig. 3A is deposited BioImage Archive, BioImages accession number S-BIAD2987. The source data of this paper are collected in the following database record: biostudies:S-SCDT-10_1038-S44318-026-00802-3.

## References

[CR1] Abramson J, Adler J, Dunger J, Evans R, Green T, Pritzel A, Ronneberger O, Willmore L, Ballard AJ, Bambrick J et al (2024) Accurate structure prediction of biomolecular interactions with AlphaFold 3. Nature 630:493–500 38718835 10.1038/s41586-024-07487-w PMC11168924

[CR2] Baskaran S, Ragusa MJ, Boura E, Hurley JH (2012) Two-site recognition of phosphatidylinositol 3-phosphate by PROPPINs in autophagy. Mol Cell 47:339–348 22704557 10.1016/j.molcel.2012.05.027 PMC3595537

[CR3] Bepler T, Morin A, Rapp M, Brasch J, Shapiro L, Noble AJ, Berger B (2019) Positive-unlabeled convolutional neural networks for particle picking in cryo-electron micrographs. Nat Methods 16:1153–1160 31591578 10.1038/s41592-019-0575-8 PMC6858545

[CR4] Bieber A, Capitanio C, Erdmann PS, Fiedler F, Beck F, Lee C-W, Li D, Hummer G, Schulman BA, Baumeister W et al (2022) In situ structural analysis reveals membrane shape transitions during autophagosome formation. Proc Natl Acad Sci USA 119:e2209823119 36122245 10.1073/pnas.2209823119 PMC9522377

[CR5] Biriukov D, Javanainen M (2023) Efficient simulations of solvent asymmetry across lipid membranes using flat-bottom restraints. J Chem Theory Comput 19:6332–6341 37651714 10.1021/acs.jctc.3c00614 PMC10537000

[CR6] Boyd KJ, May ER (2018) BUMPy: a model-independent tool for constructing lipid bilayers of varying curvature and composition. J Chem Theory Comput 14:6642–6652 30431272 10.1021/acs.jctc.8b00765 PMC6545900

[CR7] Chowdhury S, Otomo C, Leitner A, Ohashi K, Aebersold R, Lander GC, Otomo T (2018) Insights into autophagosome biogenesis from structural and biochemical analyses of the ATG2A-WIPI4 complex. Proc Natl Acad Sci USA 115:E9792–E9801 30185561 10.1073/pnas.1811874115 PMC6196511

[CR8] Chumpen Ramirez S, Gómez-Sánchez R, Verlhac P, Hardenberg R, Margheritis E, Cosentino K, Reggiori F, Ungermann C (2023) Atg9 interactions via its transmembrane domains are required for phagophore elongation during autophagy. Autophagy 19:1459–1478 36354155 10.1080/15548627.2022.2136340 PMC10241002

[CR9] Emsley P, Cowtan K (2004) Coot: model-building tools for molecular graphics. Acta Crystallogr Sect D 60:2126–2132 15572765 10.1107/S0907444904019158

[CR10] Gómez-Sánchez R, Ramirez SC, Duarte PV, Hu Y, Mari M, Olschewski K, Hardenberg R, Fromme JC, Ungermann C, Reggiori F (2025) Establishment of the phagophore–ERES membrane contact site initiates phagophore elongation. Nat Struct Mol Biol 32:2319–2334. 1–16 40775526 10.1038/s41594-025-01621-6 PMC12618252

[CR11] Gómez-Sánchez R, Rose J, Guimarães R, Mari M, Papinski D, Rieter E, Geerts WJ, Hardenberg R, Kraft C, Ungermann C et al (2018) Atg9 establishes Atg2-dependent contact sites between the endoplasmic reticulum and phagophores. J Cell Biol 217:2743–2763 29848619 10.1083/jcb.201710116 PMC6080931

[CR12] Guimaraes RS, Delorme-Axford E, Klionsky DJ, Reggiori F (2015) Assays for the biochemical and ultrastructural measurement of selective and nonselective types of autophagy in the yeast *Saccharomyces cerevisiae*. Methods 75:141–150 25484341 10.1016/j.ymeth.2014.11.023

[CR13] Hao, L, Midorikawa, T, Ogasawara, Y, Hama, Y, Lang, H, Noda, NN, and Suzuki, K (2025) Reversible one-way lipid transfer at ER–autophagosome membrane contact sites via bridge-like lipid transfer protein Atg2. J Cell Biol 225:e202506039 10.1083/jcb.202506039 41805856

[CR14] Huang J, Rauscher S, Nawrocki G, Ran T, Feig M, Groot BL, de, Grubmüller H, MacKerell AD (2017) CHARMM36m: an improved force field for folded and intrinsically disordered proteins. Nat Methods 14:71–73 27819658 10.1038/nmeth.4067 PMC5199616

[CR15] Humphrey W, Dalke A, Schulten K (1996) VMD: visual molecular dynamics. J Mol Graph 14:33–38 8744570 10.1016/0263-7855(96)00018-5

[CR16] Hunter JD (2007) Matplotlib: a 2D graphics environment. Comput Sci Eng 9:90–95

[CR17] Janke C, Magiera MM, Rathfelder N, Taxis C, Reber S, Maekawa H, Moreno-Borchart A, Doenges G, Schwob E, Schiebel E et al (2004) A versatile toolbox for PCR-based tagging of yeast genes: new fluorescent proteins, more markers and promoter substitution cassettes. Yeast 21:947–962 15334558 10.1002/yea.1142

[CR18] Kang Y, Lehmann KS, Long H, Jefferson A, Purice M, Freeman M, Clark S (2025) Structural basis of lipid transfer by a bridge-like lipid-transfer protein. Nature 642:242–249 40269155 10.1038/s41586-025-08918-y PMC13533485

[CR19] Kotani T, Kirisako H, Koizumi M, Ohsumi Y, Nakatogawa H (2018) The Atg2-Atg18 complex tethers pre-autophagosomal membranes to the endoplasmic reticulum for autophagosome formation. Proc Natl Acad Sci 115:201806727 10.1073/pnas.1806727115 PMC6187169 30254161

[CR20] Krick R, Busse RA, Scacioc A, Stephan M, Janshoff A, Thumm M, Kühnel K (2012) Structural and functional characterization of the two phosphoinositide binding sites of PROPPINs, a β-propeller protein family. Proc Natl Acad Sci USA 109:E2042–E2049 22753491 10.1073/pnas.1205128109 PMC3409749

[CR21] Kroon PC, Grünewald F, Barnoud J, Tilburg M, van, Brasnett C, Souza PC, Wassenaar TA, Marrink SJ (2025) Martinize2 and Vermouth provide a unified framework for molecular topology generation. eLife 12:RP90627 41263305 10.7554/eLife.90627 PMC12634043

[CR22] LaCava J, Jiang H, Rout MP (2016) Protein complex affinity capture from cryomilled mammalian cells. J Vis Exp: JoVE 9:54518 10.3791/54518 PMC5226390 28060343

[CR23] Li P, Lees JA, Lusk CP, Reinisch KM (2020) Cryo-EM reconstruction of a VPS13 fragment reveals a long groove to channel lipids between membranes. J Cell Biol 219:e202001161 32182622 10.1083/jcb.202001161 PMC7199853

[CR24] Liebschner D, Afonine PV, Baker ML, Bunkóczi G, Chen VB, Croll TI, Hintze B, Hung L-W, Jain S, McCoy AJ et al (2019) Macromolecular structure determination using X-rays, neutrons and electrons: recent developments in Phenix. Acta Crystallogr Sect D 75:861–877 10.1107/S2059798319011471 PMC6778852 31588918

[CR25] Lomize MA, Pogozheva ID, Joo H, Mosberg HI, Lomize AL (2012) OPM database and PPM web server: resources for positioning of proteins in membranes. Nucleic Acids Res 40:D370–D376 21890895 10.1093/nar/gkr703 PMC3245162

[CR26] Maeda S, Otomo C, Otomo T (2019) The autophagic membrane tether ATG2A transfers lipids between membranes. eLife 8:e45777 31271352 10.7554/eLife.45777 PMC6625793

[CR27] Mann D, Fromm SA, Martinez-Sanchez A, Gopaldass N, Choy R, Mayer A, Sachse C (2023) Atg18 oligomer organization in assembled tubes and on lipid membrane scaffolds. Nat Commun 14:8086 38057304 10.1038/s41467-023-43460-3 PMC10700546

[CR28] Mari M, Griffith J, Rieter E, Krishnappa L, Klionsky DJ, Reggiori F (2010) An Atg9-containing compartment that functions in the early steps of autophagosome biogenesis. J Cell Biol 190:1005–1022 20855505 10.1083/jcb.200912089 PMC3101592

[CR29] Meng EC, Goddard TD, Pettersen EF, Couch GS, Pearson ZJ, Morris JH, Ferrin TE (2023) UCSF ChimeraX: Tools for structure building and analysis. Protein Sci 32:e4792 37774136 10.1002/pro.4792 PMC10588335

[CR30] Michaud-Agrawal N, Denning EJ, Woolf TB, Beckstein O (2011) MDAnalysis: A toolkit for the analysis of molecular dynamics simulations. J Comput Chem 32:2319–2327 21500218 10.1002/jcc.21787 PMC3144279

[CR31] Nakatogawa H (2020) Mechanisms governing autophagosome biogenesis. Nat Rev Mol Cell Biol 21:439–458 32372019 10.1038/s41580-020-0241-0

[CR32] Nguyen A, Lugarini F, David C, Hosnani P, Alagöz Ç, Friedrich A, Schlütermann D, Knotkova B, Patel A, Parfentev I et al (2023) Metamorphic proteins at the basis of human autophagy initiation and lipid transfer. Mol Cell 83:2077–2090.e12 37209685 10.1016/j.molcel.2023.04.026

[CR33] Nieto, V, Crowley, J, Santos, D, and Monticelli, L (2023) Birth of an organelle: molecular mechanism of lipid droplet biogenesis. Preprint at https://www.biorxiv.org/content/10.1101/2023.07.28.550987v1

[CR34] Obara K, Sekito T, Niimi K, Ohsumi Y (2008) The Atg18-Atg2 complex is recruited to autophagic membranes via phosphatidylinositol 3-phosphate and exerts an essential function*. J Biol Chem 283:23972–23980 18586673 10.1074/jbc.M803180200 PMC3259791

[CR35] Olivas TJ, Wu Y, Yu S, Luan L, Choi P, Guinn ED, Nag S, Camilli PVD, Gupta K, Melia TJ (2023) ATG9 vesicles comprise the seed membrane of mammalian autophagosomes. J Cell Biol 222:e202208088 37115958 10.1083/jcb.202208088 PMC10148236

[CR36] Osawa T, Ishii Y, Noda NN (2020) Human ATG2B possesses a lipid transfer activity which is accelerated by negatively charged lipids and WIPI4. Genes Cells 25:65–70 31721365 10.1111/gtc.12733

[CR37] Osawa T, Kotani T, Kawaoka T, Hirata E, Suzuki K, Nakatogawa H, Ohsumi Y, Noda NN (2019) Atg2 mediates direct lipid transfer between membranes for autophagosome formation. Nat Struct Mol Biol 26:281–288 30911189 10.1038/s41594-019-0203-4

[CR38] Parrinello M, Rahman A (1981) Polymorphic transitions in single crystals: a new molecular dynamics method. J Appl Phys 52:7182–7190

[CR39] Pettersen EF, Goddard TD, Huang CC, Meng EC, Couch GS, Croll TI, Morris JH, Ferrin TE (2020) UCSF ChimeraX: structure visualization for researchers, educators, and developers. Protein Sci 30:70–82 32881101 10.1002/pro.3943 PMC7737788

[CR66] Pfaffenwimmer T, Reiter W, Brach T, Nogellova V, Papinski D, Schuschnig M, Abert C, Ammerer G, Martens S, Kraft C (2014) Hrr25 kinase promotes selective autophagy by phosphorylating the cargo receptor Atg19. EMBO Rep 15:862–870 10.15252/embr.201438932 PMC4197043 24968893

[CR40] Punjani A, Fleet DJ (2021) 3D variability analysis: resolving continuous flexibility and discrete heterogeneity from single particle cryo-EM. J Struct Biol 213:107702 33582281 10.1016/j.jsb.2021.107702

[CR41] Punjani A, Rubinstein JL, Fleet DJ, Brubaker MA (2017) cryoSPARC: algorithms for rapid unsupervised cryo-EM structure determination. Nat Methods 14:290–296 28165473 10.1038/nmeth.4169

[CR42] Punjani A, Zhang H, Fleet DJ (2020) Non-uniform refinement: adaptive regularization improves single-particle cryo-EM reconstruction. Nat Methods 17:1214–1221 33257830 10.1038/s41592-020-00990-8

[CR43] Reinisch KM, Camilli PD, Melia TJ (2025) Lipid dynamics at membrane contact sites. Annu Rev Biochem 94:479–502 40067957 10.1146/annurev-biochem-083024-122821

[CR44] Ren J, Liang R, Wang W, Zhang D, Yu L, Feng W (2020) Multi-site-mediated entwining of the linear WIR-motif around WIPI β-propellers for autophagy. Nat Commun 11:2702 32483132 10.1038/s41467-020-16523-y PMC7264293

[CR45] Rieter E, Vinke F, Bakula D, Cebollero E, Ungermann C, Proikas-Cezanne T, Reggiori F (2013) Atg18 function in autophagy is regulated by specific sites within its β-propeller. J Cell Sci 126:593–604 23230146 10.1242/jcs.115725

[CR46] Sakai, Y, Matoba, K, Kotani, T, Hao, L, Suzuki, K, Kakuta, C, Sugita, Y, Osawa, T, Nakatogawa, H, and Noda, NN (2025) Mechanism of bridge-type phospholipid transfer by Atg2 for autophagosome biogenesis. Preprint at https://www.biorxiv.org/content/10.1101/2025.05.24.655882v1

[CR47] Sánchez-Wandelmer J, Kriegenburg F, Rohringer S, Schuschnig M, Gómez-Sánchez R, Zens B, Abreu S, Hardenberg R, Hollenstein D, Gao J et al (2017) Atg4 proteolytic activity can be inhibited by Atg1 phosphorylation. Nat Commun 8:295 28821724 10.1038/s41467-017-00302-3 PMC5562703

[CR48] Sawa-Makarska J, Baumann V, Coudevylle N, Bülow S, von, Nogellova V, Abert C, Schuschnig M, Graef M, Hummer G, Martens S (2020) Reconstitution of autophagosome nucleation defines Atg9 vesicles as seeds for membrane formation. Science 369:eaaz7714 32883836 10.1126/science.aaz7714 PMC7610778

[CR49] Schindelin J, Arganda-Carreras I, Frise E, Kaynig V, Longair M, Pietzsch T, Preibisch S, Rueden C, Saalfeld S, Schmid B et al (2012) Fiji: an open-source platform for biological-image analysis. Nat Methods 9:676–682 22743772 10.1038/nmeth.2019 PMC3855844

[CR50] Schütter M, Giavalisco P, Brodesser S, Graef M (2020) Local fatty acid channeling into phospholipid synthesis drives phagophore expansion during autophagy. Cell 180:135–149.e14 31883797 10.1016/j.cell.2019.12.005

[CR51] Souza PCT, Alessandri R, Barnoud J, Thallmair S, Faustino I, Grünewald F, Patmanidis I, Abdizadeh H, Bruininks BMH, Wassenaar TA et al (2021) Martini 3: a general purpose force field for coarse-grained molecular dynamics. Nat Methods 18:382–388 33782607 10.1038/s41592-021-01098-3 PMC12554258

[CR52] Spoel DVD, Lindahl E, Hess B, Groenhof G, Mark AE, Berendsen HJC (2005) GROMACS: fast, flexible, and free. J Comput Chem 26:1701–1718 16211538 10.1002/jcc.20291

[CR53] Srinivasan S, Álvarez D, Peter ATJ, Vanni S (2024) Unbiased MD simulations identify lipid binding sites in lipid transfer proteins. J Cell Biol 223:e202312055 39105757 10.1083/jcb.202312055 PMC11303870

[CR54] Stefan CJ, Blumer KJ (1999) A syntaxin homolog encoded by VAM3 mediates down-regulation of a yeast G protein-coupled receptor*. J Biol Chem 274:1835–1841 9880567 10.1074/jbc.274.3.1835

[CR55] Suzuki K, Akioka M, Kondo-Kakuta C, Yamamoto H, Ohsumi Y (2013) Fine mapping of autophagy-related proteins during autophagosome formation in *Saccharomyces cerevisiae*. J Cell Sci 126:2534–2544 23549786 10.1242/jcs.122960

[CR56] Valverde DP, Yu S, Boggavarapu V, Kumar N, Lees JA, Walz T, Reinisch KM, Melia TJ (2019) ATG2 transports lipids to promote autophagosome biogenesis. J Cell Biol 218:1787–1798 30952800 10.1083/jcb.201811139 PMC6548141

[CR57] Vargas JNS, Hamasaki M, Kawabata T, Youle RJ, Yoshimori T (2023) The mechanisms and roles of selective autophagy in mammals. Nat Rev Mol Cell Biol 24:167–185 36302887 10.1038/s41580-022-00542-2

[CR58] Vliet, van AR, Chiduza GN, Maslen SL, Pye VE, Joshi D, Tito SD, Jefferies HBJ, Christodoulou E, Roustan C, Punch E et al (2022) ATG9A and ATG2A form a heteromeric complex essential for autophagosome formation. Mol Cell 82:4324–4339.e8 36347259 10.1016/j.molcel.2022.10.017

[CR59] Wang Y, Dahmane S, Ti R, Mai X, Zhu L, Carlson L-A, Stjepanovic G (2025) Structural basis for lipid transfer by the ATG2A–ATG9A complex. Nat Struct Mol Biol 32:35–47 39174844 10.1038/s41594-024-01376-6

[CR60] Wassenaar TA, Ingólfsson HI, Böckmann RA, Tieleman DP, Marrink SJ (2015) Computational lipidomics with insane: a versatile tool for generating custom membranes for molecular simulations. J Chem Theory Comput 11:2144–2155 26574417 10.1021/acs.jctc.5b00209

[CR61] Watanabe Y, Kobayashi T, Yamamoto H, Hoshida H, Akada R, Inagaki F, Ohsumi Y, Noda NN (2012) Structure-based analyses reveal distinct binding sites for Atg2 and phosphoinositides in Atg18*. J Biol Chem 287:31681–31690 22851171 10.1074/jbc.M112.397570 PMC3442503

[CR62] Waterhouse, Bertoni A, Bienert M, Studer S, Tauriello G, Gumienny G, Heer R, Beer FT, de TAP, Rempfer C, Bordoli L et al (2018) SWISS-MODEL: homology modelling of protein structures and complexes. Nucleic Acids Res 46:W296–W303 29788355 10.1093/nar/gky427 PMC6030848

[CR63] Williams CJ, Headd JJ, Moriarty NW, Prisant MG, Videau LL, Deis LN, Verma V, Keedy DA, Hintze BJ, Chen VB et al (2018) MolProbity: more and better reference data for improved all-atom structure validation. Protein Sci 27:293–315 29067766 10.1002/pro.3330 PMC5734394

[CR67] Veit M, Laage R, Dietrich L, Wang L & Ungermann C (2001) Vac8p release from the SNARE complex and its palmitoylation are coupled and essential for vacuole fusion. EMBO J. 20:3145–3155 10.1093/emboj/20.12.3145 PMC150195 11406591

[CR64] Yamamoto H, Kakuta S, Watanabe TM, Kitamura A, Sekito T, Kondo-Kakuta C, Ichikawa R, Kinjo M, Ohsumi Y (2012) Atg9 vesicles are an important membrane source during early steps of autophagosome formation. J Cell Biol 198:219–233 22826123 10.1083/jcb.201202061 PMC3410421

[CR65] Zheng J-X, Li Y, Ding Y-H, Liu J-J, Zhang M-J, Dong M-Q, Wang H-W, Yu L (2017) Architecture of the ATG2B-WDR45 complex and an aromatic Y/HF motif crucial for complex formation. Autophagy 13:1870–1883 28820312 10.1080/15548627.2017.1359381 PMC5788475

