# Supplementary material for: Atg18 interaction positions Atg2 for efficient lipid transfer into phagophore elongation
Source: EMBO J. 2026 May 20;45(12):4034–60. doi: 10.1038/s44318-026-00802-3 (PMC13269710; doi:10.1038/s44318-026-00802-3)
Supplement: Supplementary file 1 — Appendix [file 44318_2026_802_MOESM1_ESM.pdf]

Appendix for

Atg18 interaction positions Atg2 for efficient lipid transfer into phagophore elongation

Sabrina Chumpen Ramirez, Dmitry Shvarev, Prado Vargas Duarte, Yara Ahmed, Jana Milach, Emma Lang, Stefan Kuchenbuch, Stefano Vanni, Fulvio Reggiori, Arne Moeller and Christian Ungermann

Table of Contents **page**

**Appendix Fig S1**	Atg2 N-terminal instability	2
**Appendix Fig S2**	Comparison of the Atg18-Atg2 structure, elucidated by cryo-EM, with an AlphaFold3 (AF3) model	3
**Appendix Fig S3**	Atg18 WIR-binding pockets	4
**Appendix Fig S4**	Pho8Δ60 assays of tagged -versions of the ATG proteins used in this study	6
**Appendix Fig S5**	Correlation analysis of giant Ape1 cargo size and phagophore elongation	7
**Appendix Fig S6**	Analysis of ER- PAS association in cells expressing the Atg2 922-934 deletion	8

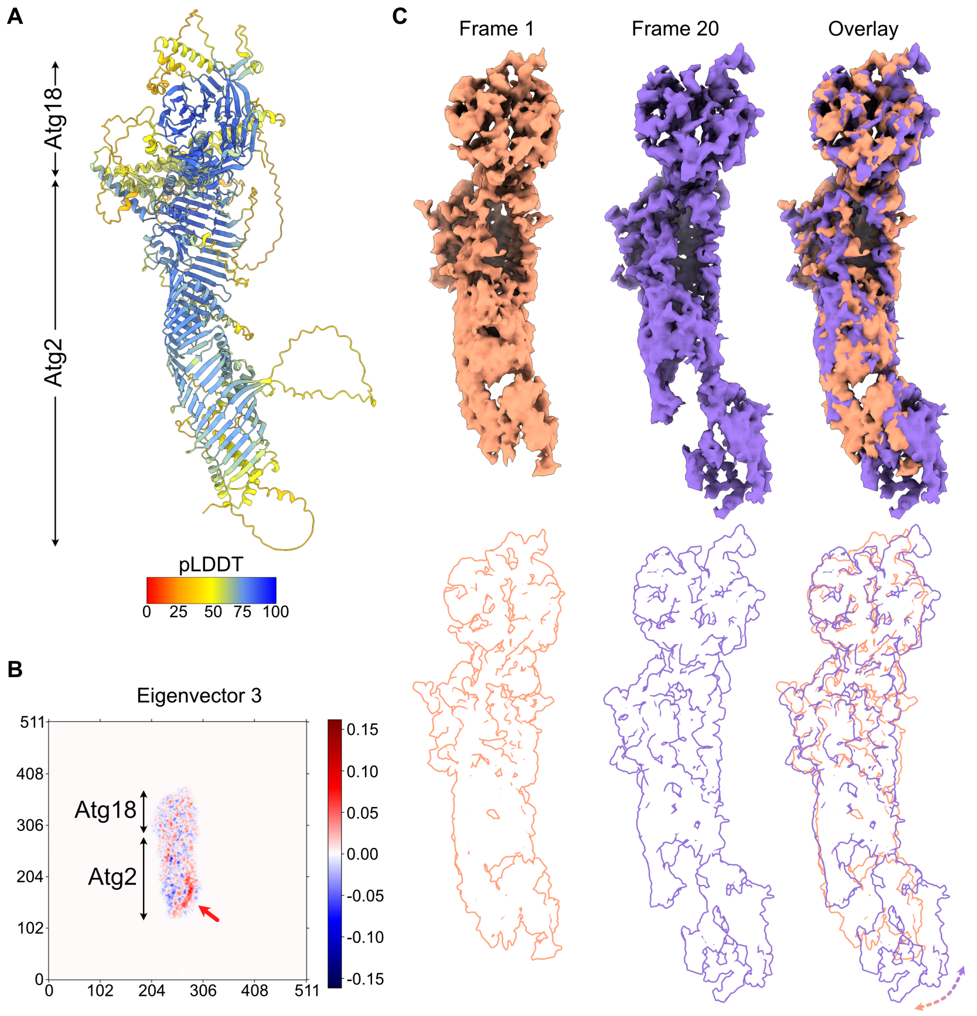

Appendix Figure S1. **Atg2 N-terminal instability. (A)** AlphaFold3 model of the Atg2-Atg18 complex shown as ribbons colored by the per-residue "predicted local distance difference test" pLDDT score. 50% or less confidence of pLDDT indicates low structural stability of the respective region of the protein. **(B)** Orthogonal slice of one of the analyzed variability components using the cryoSPARC 3D variability analysis (3DVA) for the Atg2-Atg18 complex, with red and blue corresponding to positive and negative values. Variability in the N-terminal part of Atg2 is marked by a red arrow. **(C)** Density maps generated by 3DVA for the Atg2-Atg18 complex at negative (salmon pink color, frame 1) and positive (purple color, frame 19) positions along the variability component from **(B)**, as well as their overlay. Top panels show map surfaces and bottom panels their outlines, highlighting the flexibility of the N-terminal region (double-headed arrow).

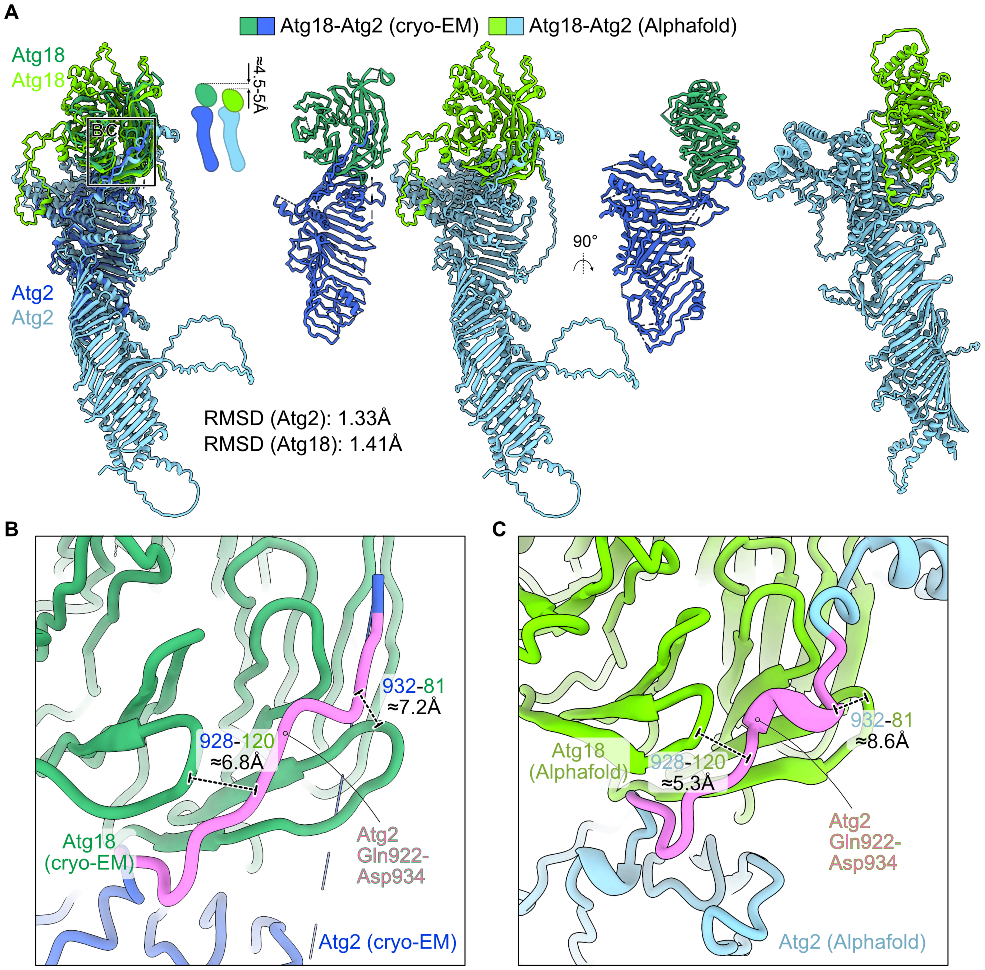

Appendix Figure S2. Comparison of the Atg18-Atg2 structure, elucidated by cryo-EM, with an AlphaFold3 (AF3) model. (**A**) Comparison of the Atg2-Atg18 complex resolved by cryo-EM (colored dark blue and dark green respectively) with the corresponding AF3 model (Atg2, light blue, Atg18, light green). Left, superposition of the structure and model; center and right, individual structures viewed from different angles along the Atg2 tunnel. Root mean square deviation (RMSD), calculated for C-alpha atoms of Atg2 and Atg18 is indicated. The schematic in the left panel shows that, in the cryo-EM structure, Atg18 is positioned ~5 Å further from Atg2 compared with the AF3 model (colors as in the models). (**B**) Close-up view of the Atg2-Atg18 binding interface (see Figure 2) from the cryo-EM structure. Colors are as in (A), while the 922-934 region of Atg2 is colored pink. Distances between selected residues of Atg2 and Atg18 are indicated. (**C**) Close-up view of the Atg2-Atg18 interface shown in (B) from the AF3 model. Colors are as in (A), the 922-934 region of Atg2 is colored pink. Distances between selected residues of Atg2 and Atg18 are indicated.

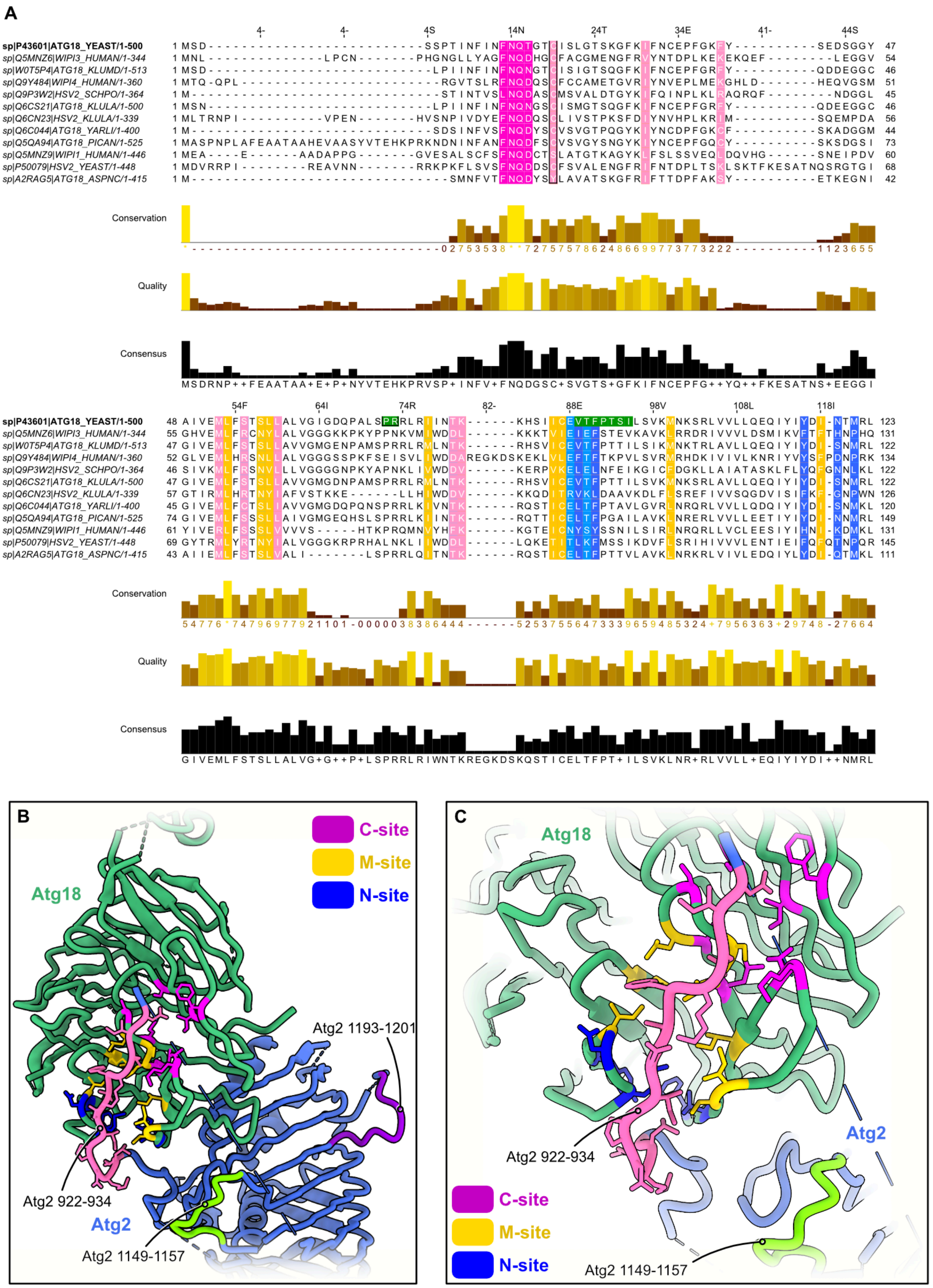

Appendix Figure S3. Atg18 WIR-binding pockets. (**A**) Sequence alignment of the PROPPINs showing the residues involved in the interaction between PROPPINs and the WIR-motif of Atg2. Residues

corresponding to the N-site, M-site and C-site are highlighted in dark blue, yellow and pink, respectively. Residues in Atg18 previously described as essential for interaction with Atg2 are highlighted in green. (B) and (C). Structure of the Atg2-Atg18 complex (Atg18 colored forest green, Atg2 colored dodger blue) showing the WIR-motif binding sites in Atg18 (N-site: dark blue, M-site: yellow, C-site: pink) and the Atg18-interaction region 922-934 of Atg2, highlighted in salmon pink. Atg2 regions 1149-1157 (this study) and 1193-1201 (reported in Ren *et al.*, 2020) are highlighted in chartreuse and purple, respectively.

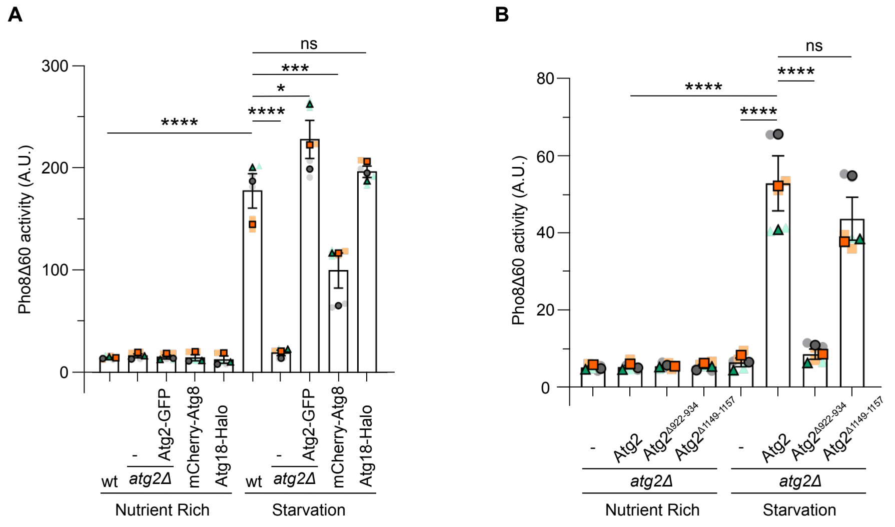

Appendix Figure S4. Pho8Δ60 assays of tagged -versions of the ATG proteins used in this study. (**A**). Pho8Δ60 activity assay strains endogenously expressing Atg18-Halo (CU16317) or mCherry-Atg8 (CU16316) (mCh-Atg8), or an *atg2Δ* knockout back-transformed with an integrative plasmid carrying Atg2-GFP under the control of the endogenous promoter (CU15429). (**B**) Pho8Δ60 activity assay from Pho8Δ60 *atg2Δ* strain (*atg2Δ*) (CU15300) transformed with integrative plasmids carrying the indicated Atg2 variant with no tags and under the control of the endogenous promoter (CU15429, CU15430, CU15432). The Wild-type (wt) (CU10489) and *atg2Δ* (CU15309) Pho8Δ60 strains were used as the positive and negative control, respectively. In both (A) and (B), cells were grown in YPD and analyzed under nutrient rich conditions or nitrogen starved by incubation in SD-N for 3 h. The plot shows the enzymatic activity calculated as arbitrary units (A.U.). The data correspond to the mean value and the standard error of measurements from three independent experiments. The statistical analysis was performed by two-way ANOVA using Dunnett´s correction for multiple comparisons, and a 95% confidence interval. In data (A) the adjusted P values are as follow: wt Starvation vs wt Nutrient rich, $P < 0.0001$; wt Starvation vs *atg2Δ* Starvation, $P < 0.0001$; wt Starvation vs *atg2Δ* Atg2-GFP Starvation, $P = 0.0108$; wt Starvation vs mCherry-Atg8 Starvation, $P = 0.0002$; wt Starvation vs Atg18-Halo Starvation, $P = 0.6723$. In data (B) the adjusted P values are as follow: *atg2Δ* Atg2_Starvation vs *atg2Δ*_Starvation, $P < 0.0001$; *atg2Δ* Atg2_Starvation vs *atg2Δ*_Nutrient rich, $P < 0.0001$; *atg2Δ* Atg2 Starvation vs *atg2Δ* Atg2$^{Δ922\text{-}934}$ Starvation), $P < 0.0001$; *atg2Δ* Atg2 Starvation vs *atg2Δ* Atg2$^{Δ1149–1157}$ Starvation), $P = 0.3795$.

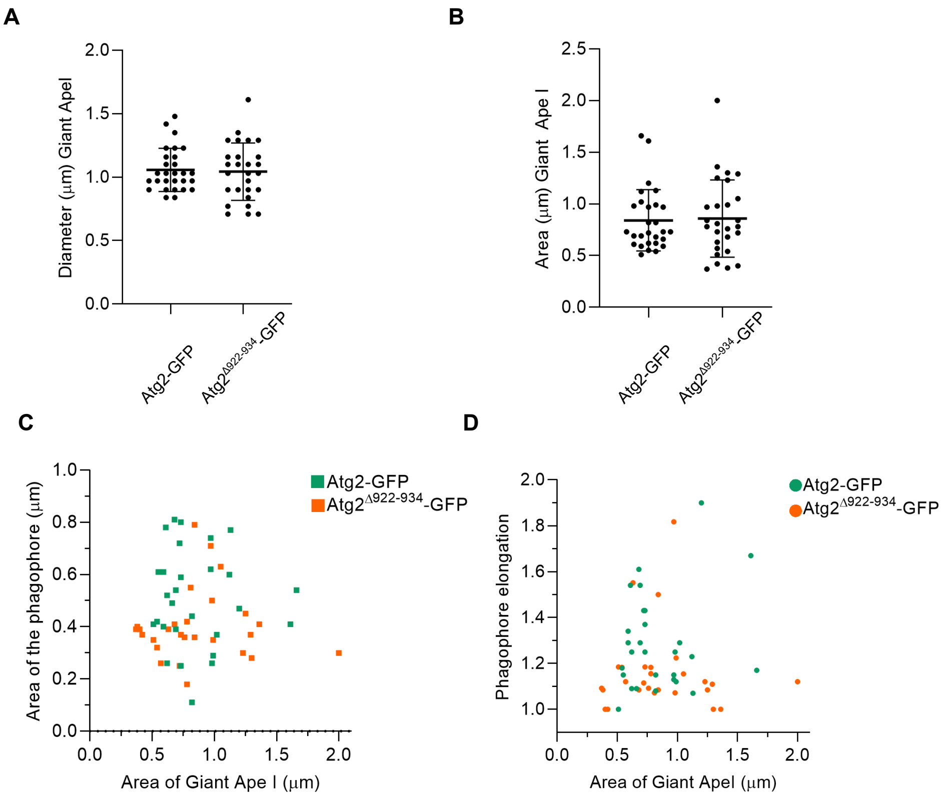

Appendix Figure S5. Correlation analysis of giant Ape 1cargo size and phagophore elongation. (**A**) and (**B**). Measurement of the diameter (A) or area (B) of the Ape1 cargo surrounded by the phagophores analyzed in Figure 4C. The data shows the mean value and the SD of at least 27 measurements. (**C**) and (**D**). Correlation plot between the area of the Ape1 cargo and the phagophore area (C) or the phagophore elongation (D), defined as described in Figure 4B. Data from cells expressing Atg2-GFP and Atg2$^{\Delta 922\text{-}934}$-GFP are labeled in green and orange, respectively.

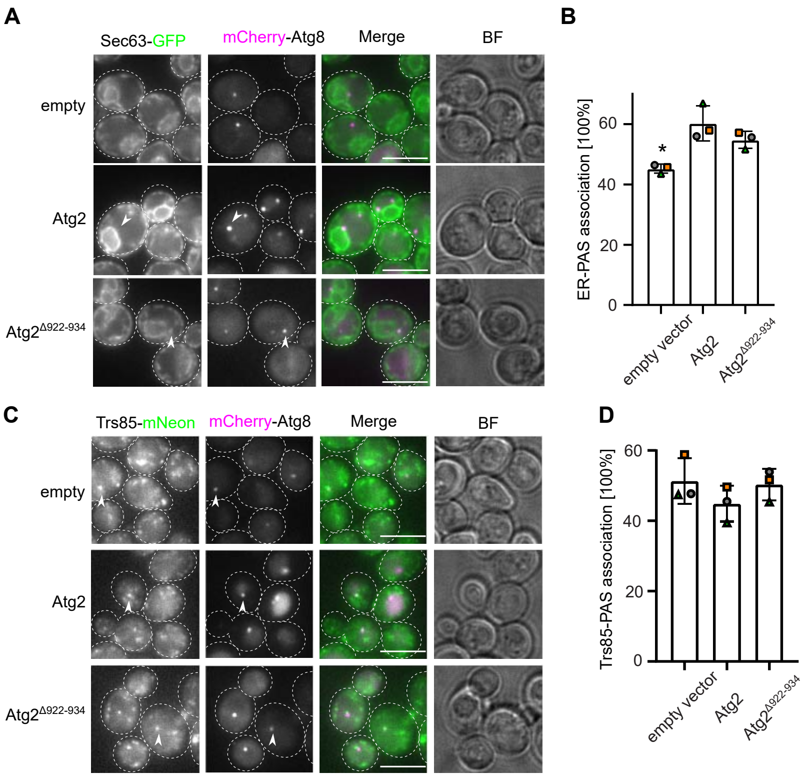

Appendix Figure S6. Analysis of ER- PAS association in cells expressing the Atg2 922-934 deletion. (**A**) The *atg2Δtrs85Δ* strains carrying endogenous Sec63-GFP, mCherry-Atg8 and transformed with an integrative empty vector (PVY118), or a pslamid carrying Atg2-TAP (PVY123) or Atg2Δ922-934-TAP (PVY124), were grown in YPD and then transferred in SD-N medium for 1 h to induce autophagy. Images were collected with a Oxford Nanoimager microscope system. Associations between ER and PAS are indicated with white arrowheads. Cells boundaries are marked (white dotted lines). BF: Bright field. Scale bar: 5 µm. (**B**) Quantification of (A). The percentage of mCherry-Atg8-positive structures associated with the ER was determined by analyzing ≥100 cells from three independent experiments. The plot shows the mean value and the standard error of the three independent measurements. Statistical analysis was conducted by two-tailed unpaired *t*-test with Welch's correction with 95% confidence intervals. *P* values are as follows: *atg2Δtrs85Δ* empty vector (PVY118) vs Atg2 (PVY123), $P = 0.0396$. (**C**) The atg2Δ strains expressing endogenous Trs85-mNeon, mCherry-Atg8 and transformed with an integrative empty vector (RGY1024), or a plasmid carrying Atg2-TAP (PVY127) or Atg2$^{\Delta 922\text{-}934}$-TAP (PVY127), were analyzed as in (A). Trs85-mNeon-positive Atg8 puncta are indicated by white arrowheads. BF: Bright field. Scale bar: 5 µm. (**D**) Quantification of (C). The percentage of Trs85-positive Atg8 puncta was determined by analyzing ≥100 cells from three independent experiments. Statistical analysis was conducted by two-tailed unpaired *t*-test with Welch's correction with 95% confidence intervals.
