# Supplementary material for: Atg18 interaction positions Atg2 for efficient lipid transfer into phagophore elongation
Source: EMBO J. 2026 May 20;45(12):4034–60. doi: 10.1038/s44318-026-00802-3 (PMC13269710; doi:10.1038/s44318-026-00802-3)
Supplement: Supplementary file 3 — Table EV2 [file 44318_2026_802_MOESM3_ESM.docx]

Table EV 2. Cryo-EM data collection, refinement and validation statistics.

|  | **Atg2-Atg18 complex (EMDB-55395, PDB-9T0C)** |
| --- | --- |
| **Data collection and processing** | |
| Magnification | 130000 |
| Voltage (kV) | 200 |
| Electron exposure (e^–^/Å^2^) | 50 |
| Defocus range (μm) | -0.8 to -1.8 |
| Pixel size (Å) | 0.92 |
| Symmetry imposed | C1 |
| Initial particle images after 2D classifications and duplicate removal (no.) | 260026 |
| Final particle images (no.) | 97431 |
| Map resolution (Å)  FSC threshold | 4.01  0.143 |
| **Refinement** | |
| Initial model used | AlphaFold; Uniprot IDs: P53855 and P43601 |
| Map resolution  min, 25th percentile, median, 75th percentile, max (Å)  FSC threshold | 3.430, 6.379, 8.194, 9.780, 63.524  0.143 |
| Map sharpening B factor (Å^2^) | -101.2 |
| Model composition  Atoms (Hydrogens)  Protein residues | 11604 (5565)  821 |
| Ligands | - |
| R.m.s. deviations  Bond lengths (Å)  Bond angles (°) | 0.002  0.581 |
| Validation  MolProbity score  Clashscore  Poor rotamers (%) | 1.88  5.34  0.34 |
| Ramachandran plot  Favored (%)  Outliers (%) | 88.38  0.13 |
