# Supplementary material for: Atg18 interaction positions Atg2 for efficient lipid transfer into phagophore elongation
Source: EMBO J. 2026 May 20;45(12):4034–60. doi: 10.1038/s44318-026-00802-3 (PMC13269710; doi:10.1038/s44318-026-00802-3)
Supplement: Supplementary file 6 — Source data Fig. 2 [file 44318_2026_802_MOESM6_ESM.zip › Figure 2/2B/README.docx]

M: Molecular weight. PageRuler Plus Prestained Protein Ladder (Thermo Scientific)

E: Protein Eluate

EC: Eluate concentrated

EG: Elution with glycine pH 3

SEC: Size exclusion Chromatography

A, B or C: SEC fractions

BSA: Bovine Serum Albumin
