# Supplementary figures and images for: Atg18 interaction positions Atg2 for efficient lipid transfer into phagophore elongation (part 1 of 3)
Source: EMBO J. 2026 May 20;45(12):4034–60. doi: 10.1038/s44318-026-00802-3 (PMC13269710; doi:10.1038/s44318-026-00802-3)

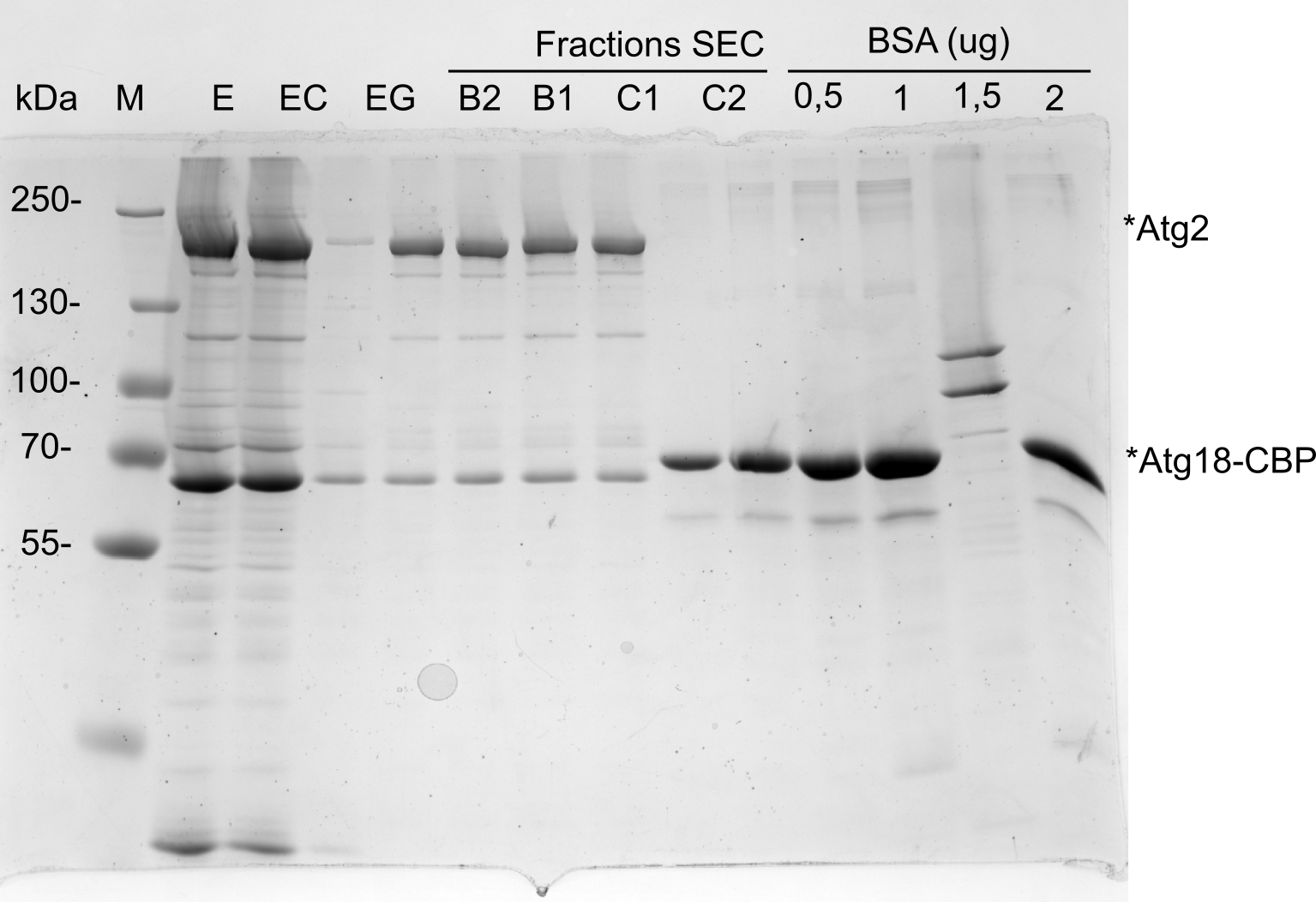

Supplement: Supplementary file 6 — Source data Fig. 2 [file 44318_2026_802_MOESM6_ESM.zip › Figure 2/2B/Replicates/SDS-PAGE_Coomassie_Atg2-Atg18 purification_Replicate_3.tiff]

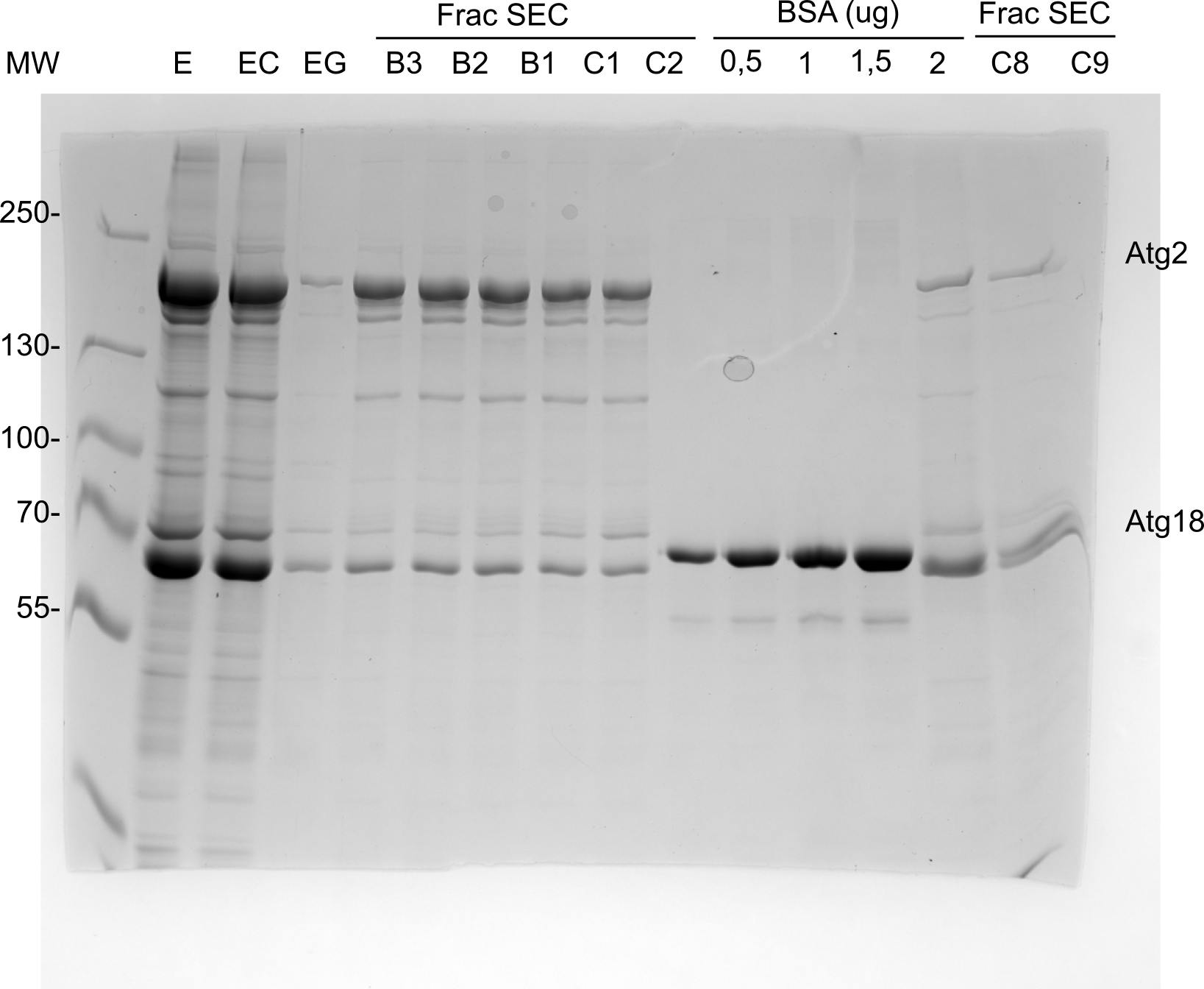

Supplement: Supplementary file 6 — Source data Fig. 2 [file 44318_2026_802_MOESM6_ESM.zip › Figure 2/2B/Replicates/SDS-PAGE_Coomassie_Atg2-Atg18 purification_Replicate_2.tiff]

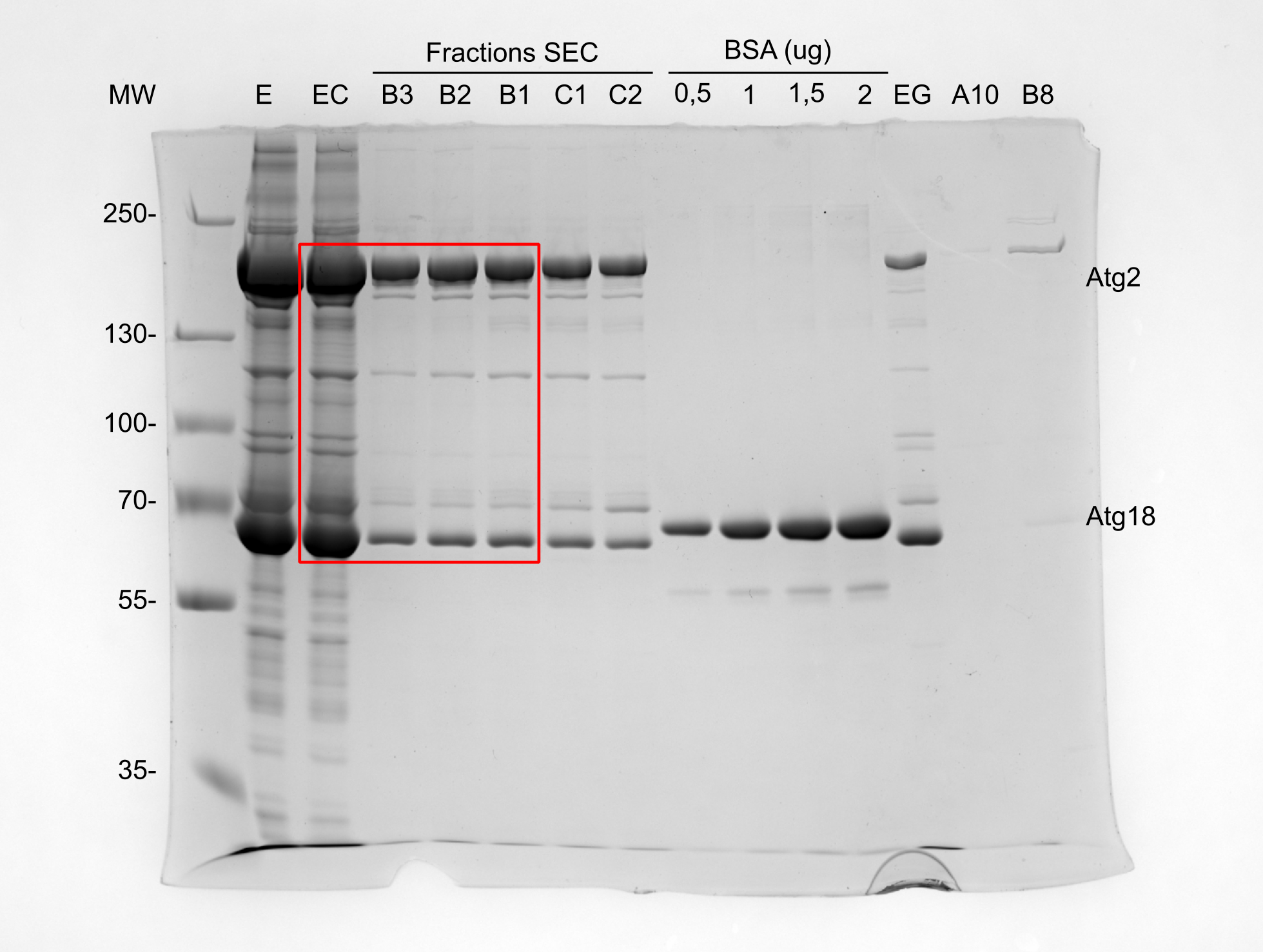

Supplement: Supplementary file 6 — Source data Fig. 2 [file 44318_2026_802_MOESM6_ESM.zip › Figure 2/2B/SDS-PAGE_Coomassie_Atg2-Atg18 purification.tiff]

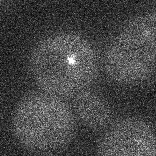

Supplement: Supplementary file 7 — Source data Fig. 3 [file 44318_2026_802_MOESM7_ESM.zip › Figure 3/Figure 3/3A/Strain # 15120_ST_4_MMStack_Pos0.ome-1.tif]

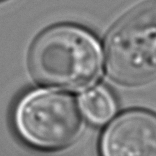

Supplement: Supplementary file 7 — Source data Fig. 3 [file 44318_2026_802_MOESM7_ESM.zip › Figure 3/Figure 3/3A/Strain # 15120_ST_REF_4_MMStack_Pos0.ome-1.tif]

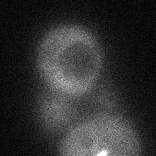

Supplement: Supplementary file 7 — Source data Fig. 3 [file 44318_2026_802_MOESM7_ESM.zip › Figure 3/Figure 3/3A/Strain # 15121_ST_6_MMStack_Pos0.ome-1.tif]

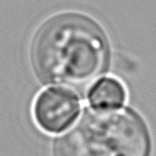

Supplement: Supplementary file 7 — Source data Fig. 3 [file 44318_2026_802_MOESM7_ESM.zip › Figure 3/Figure 3/3A/Strain # 15121_ST_REF_6_MMStack_Pos0.ome-1.tif]

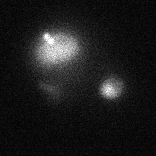

Supplement: Supplementary file 7 — Source data Fig. 3 [file 44318_2026_802_MOESM7_ESM.zip › Figure 3/Figure 3/3A/Strain # 15158_ST_1_MMStack_Pos0.ome-1.tif]

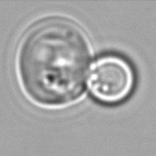

Supplement: Supplementary file 7 — Source data Fig. 3 [file 44318_2026_802_MOESM7_ESM.zip › Figure 3/Figure 3/3A/Strain # 15158_ST_REF_1_MMStack_Pos0.ome-1.tif]

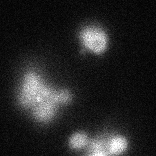

Supplement: Supplementary file 7 — Source data Fig. 3 [file 44318_2026_802_MOESM7_ESM.zip › Figure 3/Figure 3/3A/Strain # 15159_ST_2_MMStack_Pos0.ome-1.tif]

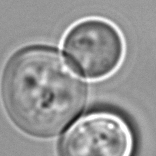

Supplement: Supplementary file 7 — Source data Fig. 3 [file 44318_2026_802_MOESM7_ESM.zip › Figure 3/Figure 3/3A/Strain # 15159_ST_REF_2_MMStack_Pos0.ome-1.tif]

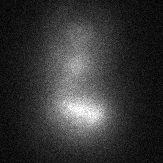

Supplement: Supplementary file 8 — Source data Fig. 4 [file 44318_2026_802_MOESM8_ESM.zip › Figure 4/4A/GiantAPE1_strain # 15159.ome.tif]

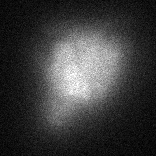

Supplement: Supplementary file 8 — Source data Fig. 4 [file 44318_2026_802_MOESM8_ESM.zip › Figure 4/4A/GiantAPE1_strain # 15121.ome.tif]

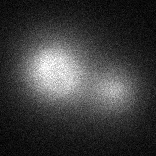

Supplement: Supplementary file 8 — Source data Fig. 4 [file 44318_2026_802_MOESM8_ESM.zip › Figure 4/4B/GiantAPE1_Round phagophore.tif]

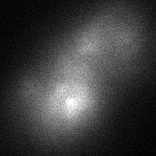

Supplement: Supplementary file 8 — Source data Fig. 4 [file 44318_2026_802_MOESM8_ESM.zip › Figure 4/4B/GiantAPE1_slightly elongated.tif]

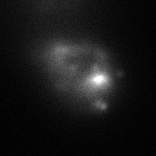

Supplement: Supplementary file 8 — Source data Fig. 4 [file 44318_2026_802_MOESM8_ESM.zip › Figure 4/4B/GiantAPE1_Elongated phagophore.tif]

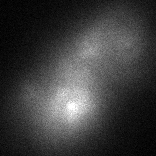

Supplement: Supplementary file 8 — Source data Fig. 4 [file 44318_2026_802_MOESM8_ESM.zip › Figure 4/4C/Strain # 15159_Analyzed cells/GiantAPE1_15159_6_1.ome-1.tif]

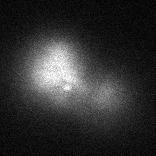

Supplement: Supplementary file 8 — Source data Fig. 4 [file 44318_2026_802_MOESM8_ESM.zip › Figure 4/4C/Strain # 15159_Analyzed cells/GiantAPE1_15159_9_5.ome-1.tif]

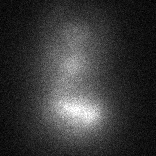

Supplement: Supplementary file 8 — Source data Fig. 4 [file 44318_2026_802_MOESM8_ESM.zip › Figure 4/4C/Strain # 15159_Analyzed cells/GiantAPE1_15159_4_1.ome-1.tif]

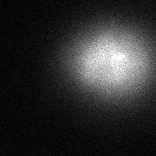

Supplement: Supplementary file 8 — Source data Fig. 4 [file 44318_2026_802_MOESM8_ESM.zip › Figure 4/4C/Strain # 15159_Analyzed cells/GiantAPE1_15159_2_2.ome-1.tif]

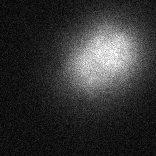

Supplement: Supplementary file 8 — Source data Fig. 4 [file 44318_2026_802_MOESM8_ESM.zip › Figure 4/4C/Strain # 15159_Analyzed cells/GiantAPE1_15159_5_3.ome-1.tif]

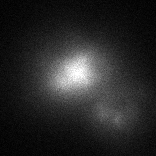

Supplement: Supplementary file 8 — Source data Fig. 4 [file 44318_2026_802_MOESM8_ESM.zip › Figure 4/4C/Strain # 15159_Analyzed cells/GiantAPE1_15159_8_2.ome-1.tif]

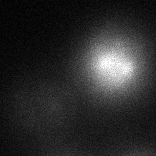

Supplement: Supplementary file 8 — Source data Fig. 4 [file 44318_2026_802_MOESM8_ESM.zip › Figure 4/4C/Strain # 15159_Analyzed cells/GiantAPE1_15159_3_3.ome-1.tif]

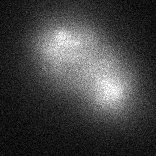

Supplement: Supplementary file 8 — Source data Fig. 4 [file 44318_2026_802_MOESM8_ESM.zip › Figure 4/4C/Strain # 15159_Analyzed cells/GiantAPE1_15159_5_2.ome-1.tif]

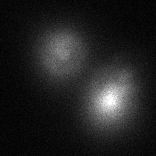

Supplement: Supplementary file 8 — Source data Fig. 4 [file 44318_2026_802_MOESM8_ESM.zip › Figure 4/4C/Strain # 15159_Analyzed cells/GiantAPE1_15159_7_2.ome-1.tif]

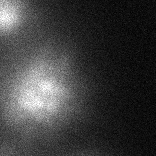

Supplement: Supplementary file 8 — Source data Fig. 4 [file 44318_2026_802_MOESM8_ESM.zip › Figure 4/4C/Strain # 15159_Analyzed cells/GiantAPE1_15159_3_1.ome-1.tif]

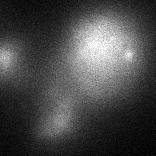

Supplement: Supplementary file 8 — Source data Fig. 4 [file 44318_2026_802_MOESM8_ESM.zip › Figure 4/4C/Strain # 15159_Analyzed cells/GiantAPE1_15159_7_4.ome-1.tif]

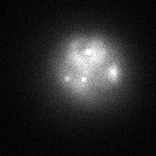

Supplement: Supplementary file 8 — Source data Fig. 4 [file 44318_2026_802_MOESM8_ESM.zip › Figure 4/4C/Strain # 15159_Analyzed cells/GiantAPE1_15159_1_2.ome-1.tif]

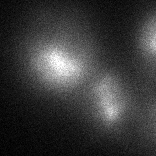

Supplement: Supplementary file 8 — Source data Fig. 4 [file 44318_2026_802_MOESM8_ESM.zip › Figure 4/4C/Strain # 15159_Analyzed cells/GiantAPE1_15159_8_4.ome-1.tif]

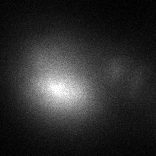

Supplement: Supplementary file 8 — Source data Fig. 4 [file 44318_2026_802_MOESM8_ESM.zip › Figure 4/4C/Strain # 15159_Analyzed cells/GiantAPE1_15159_9_1.ome-1.tif]

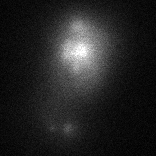

Supplement: Supplementary file 8 — Source data Fig. 4 [file 44318_2026_802_MOESM8_ESM.zip › Figure 4/4C/Strain # 15159_Analyzed cells/GiantAPE1_15159_5_1.ome-1.tif]

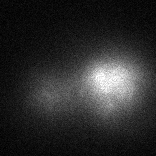

Supplement: Supplementary file 8 — Source data Fig. 4 [file 44318_2026_802_MOESM8_ESM.zip › Figure 4/4C/Strain # 15159_Analyzed cells/GiantAPE1_15159_1_1.ome-1.tif]

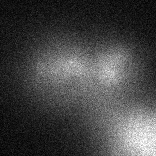

Supplement: Supplementary file 8 — Source data Fig. 4 [file 44318_2026_802_MOESM8_ESM.zip › Figure 4/4C/Strain # 15159_Analyzed cells/GiantAPE1_15159_4_5.ome-1.tif]

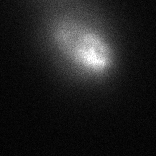

Supplement: Supplementary file 8 — Source data Fig. 4 [file 44318_2026_802_MOESM8_ESM.zip › Figure 4/4C/Strain # 15159_Analyzed cells/GiantAPE1_15159_4_3.ome-1.tif]

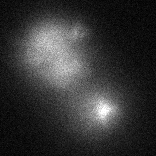

Supplement: Supplementary file 8 — Source data Fig. 4 [file 44318_2026_802_MOESM8_ESM.zip › Figure 4/4C/Strain # 15159_Analyzed cells/GiantAPE1_15159_7_3.ome-1.tif]

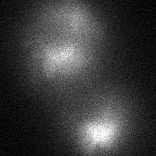

Supplement: Supplementary file 8 — Source data Fig. 4 [file 44318_2026_802_MOESM8_ESM.zip › Figure 4/4C/Strain # 15159_Analyzed cells/GiantAPE1_15159_8_5.ome-1.tif]

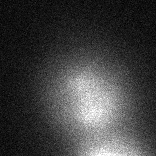

Supplement: Supplementary file 8 — Source data Fig. 4 [file 44318_2026_802_MOESM8_ESM.zip › Figure 4/4C/Strain # 15159_Analyzed cells/GiantAPE1_15159_4_2.ome-1.tif]

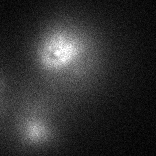

Supplement: Supplementary file 8 — Source data Fig. 4 [file 44318_2026_802_MOESM8_ESM.zip › Figure 4/4C/Strain # 15159_Analyzed cells/GiantAPE1_15159_9_2.ome-1.tif]

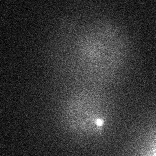

Supplement: Supplementary file 8 — Source data Fig. 4 [file 44318_2026_802_MOESM8_ESM.zip › Figure 4/4C/Strain # 15159_Analyzed cells/GiantAPE1_15159_7_1.ome-1.tif]

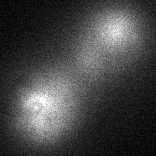

Supplement: Supplementary file 8 — Source data Fig. 4 [file 44318_2026_802_MOESM8_ESM.zip › Figure 4/4C/Strain # 15159_Analyzed cells/GiantAPE1_15159_6_2.ome-1.tif]

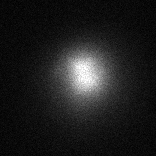

Supplement: Supplementary file 8 — Source data Fig. 4 [file 44318_2026_802_MOESM8_ESM.zip › Figure 4/4C/Strain # 15159_Analyzed cells/GiantAPE1_15159_9_4.ome-1.tif]

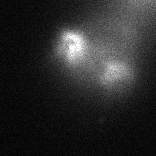

Supplement: Supplementary file 8 — Source data Fig. 4 [file 44318_2026_802_MOESM8_ESM.zip › Figure 4/4C/Strain # 15159_Analyzed cells/GiantAPE1_15159_2_1.ome-1.tif]

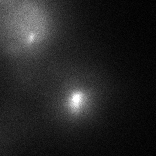

Supplement: Supplementary file 8 — Source data Fig. 4 [file 44318_2026_802_MOESM8_ESM.zip › Figure 4/4C/Strain # 15159_Analyzed cells/GiantAPE1_15159_5_4.ome-1.tif]

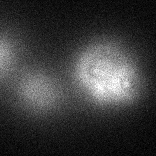

Supplement: Supplementary file 8 — Source data Fig. 4 [file 44318_2026_802_MOESM8_ESM.zip › Figure 4/4C/Strain # 15121_Analyzed cells/GiantAPE1_15121_1.ome-1.tif]

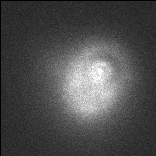

Supplement: Supplementary file 8 — Source data Fig. 4 [file 44318_2026_802_MOESM8_ESM.zip › Figure 4/4C/Strain # 15121_Analyzed cells/GiantAPE1_15121_B_3_2.ome-1.tif]

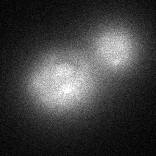

Supplement: Supplementary file 8 — Source data Fig. 4 [file 44318_2026_802_MOESM8_ESM.zip › Figure 4/4C/Strain # 15121_Analyzed cells/GiantAPE1_15121_B_1_1.ome-1.tif]

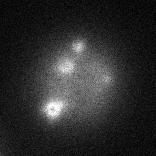

Supplement: Supplementary file 8 — Source data Fig. 4 [file 44318_2026_802_MOESM8_ESM.zip › Figure 4/4C/Strain # 15121_Analyzed cells/GiantAPE1_15121_B_4_2.ome-1.tif]

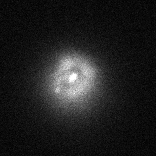

Supplement: Supplementary file 8 — Source data Fig. 4 [file 44318_2026_802_MOESM8_ESM.zip › Figure 4/4C/Strain # 15121_Analyzed cells/GiantAPE1_15121_3_3.ome-1.tif]

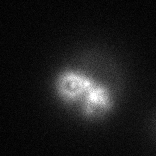

Supplement: Supplementary file 8 — Source data Fig. 4 [file 44318_2026_802_MOESM8_ESM.zip › Figure 4/4C/Strain # 15121_Analyzed cells/GiantAPE1_15121_B_5_5.ome-1.tif]

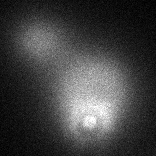

Supplement: Supplementary file 8 — Source data Fig. 4 [file 44318_2026_802_MOESM8_ESM.zip › Figure 4/4C/Strain # 15121_Analyzed cells/GiantAPE1_15121_7_1.ome-1.tif]

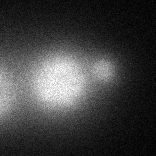

Supplement: Supplementary file 8 — Source data Fig. 4 [file 44318_2026_802_MOESM8_ESM.zip › Figure 4/4C/Strain # 15121_Analyzed cells/GiantAPE1_15121_B_1_2.ome-1.tif]

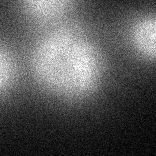

Supplement: Supplementary file 8 — Source data Fig. 4 [file 44318_2026_802_MOESM8_ESM.zip › Figure 4/4C/Strain # 15121_Analyzed cells/GiantAPE1_15121_3.ome-1.tif]

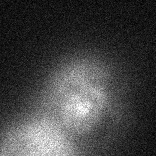

Supplement: Supplementary file 8 — Source data Fig. 4 [file 44318_2026_802_MOESM8_ESM.zip › Figure 4/4C/Strain # 15121_Analyzed cells/GiantAPE1_15121_B_5_2.ome-1.tif]

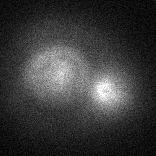

Supplement: Supplementary file 8 — Source data Fig. 4 [file 44318_2026_802_MOESM8_ESM.zip › Figure 4/4C/Strain # 15121_Analyzed cells/GiantAPE1_15121_B_3_1ome-1.tif]

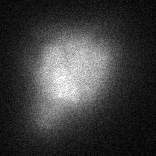

Supplement: Supplementary file 8 — Source data Fig. 4 [file 44318_2026_802_MOESM8_ESM.zip › Figure 4/4C/Strain # 15121_Analyzed cells/GiantAPE1_15121_B_5_1.ome-1.tif]

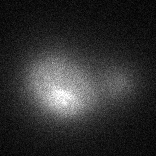

Supplement: Supplementary file 8 — Source data Fig. 4 [file 44318_2026_802_MOESM8_ESM.zip › Figure 4/4C/Strain # 15121_Analyzed cells/GiantAPE1_15121_4_1.ome-1.tif]

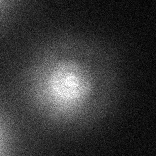

Supplement: Supplementary file 8 — Source data Fig. 4 [file 44318_2026_802_MOESM8_ESM.zip › Figure 4/4C/Strain # 15121_Analyzed cells/GiantAPE1_15121_7_2.ome-1.tif]

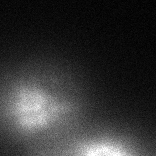

Supplement: Supplementary file 8 — Source data Fig. 4 [file 44318_2026_802_MOESM8_ESM.zip › Figure 4/4C/Strain # 15121_Analyzed cells/GiantAPE1_15121_6_2.ome-1.tif]

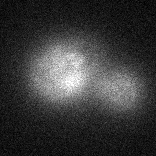

Supplement: Supplementary file 8 — Source data Fig. 4 [file 44318_2026_802_MOESM8_ESM.zip › Figure 4/4C/Strain # 15121_Analyzed cells/GiantAPE1_15121_9_1.ome-1.tif]

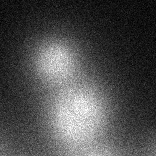

Supplement: Supplementary file 8 — Source data Fig. 4 [file 44318_2026_802_MOESM8_ESM.zip › Figure 4/4C/Strain # 15121_Analyzed cells/GiantAPE1_15121_3_2.ome-1.tif]

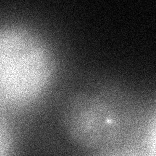

Supplement: Supplementary file 8 — Source data Fig. 4 [file 44318_2026_802_MOESM8_ESM.zip › Figure 4/4C/Strain # 15121_Analyzed cells/GiantAPE1_15121_B_6_2.ome-1.tif]

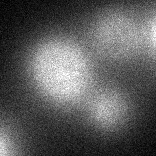

Supplement: Supplementary file 8 — Source data Fig. 4 [file 44318_2026_802_MOESM8_ESM.zip › Figure 4/4C/Strain # 15121_Analyzed cells/GiantAPE1_15121_5_1.ome-1.tif]

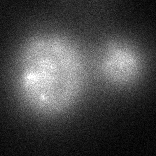

Supplement: Supplementary file 8 — Source data Fig. 4 [file 44318_2026_802_MOESM8_ESM.zip › Figure 4/4C/Strain # 15121_Analyzed cells/GiantAPE1_15121_4_3.ome-1.tif]

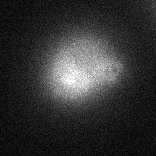

Supplement: Supplementary file 8 — Source data Fig. 4 [file 44318_2026_802_MOESM8_ESM.zip › Figure 4/4C/Strain # 15121_Analyzed cells/GiantAPE1_15121_B_4_1.ome-1.tif]

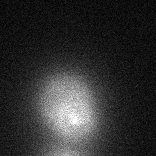

Supplement: Supplementary file 8 — Source data Fig. 4 [file 44318_2026_802_MOESM8_ESM.zip › Figure 4/4C/Strain # 15121_Analyzed cells/GiantAPE1_15121_B_5_4.ome-1.tif]

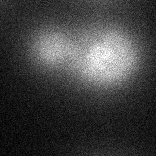

Supplement: Supplementary file 8 — Source data Fig. 4 [file 44318_2026_802_MOESM8_ESM.zip › Figure 4/4C/Strain # 15121_Analyzed cells/GiantAPE1_15121_8_2.ome-1.tif]

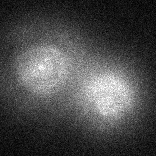

Supplement: Supplementary file 8 — Source data Fig. 4 [file 44318_2026_802_MOESM8_ESM.zip › Figure 4/4C/Strain # 15121_Analyzed cells/GiantAPE1_15121_8_3.ome-1.tif]

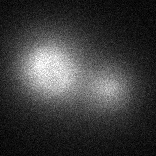

Supplement: Supplementary file 8 — Source data Fig. 4 [file 44318_2026_802_MOESM8_ESM.zip › Figure 4/4C/Strain # 15121_Analyzed cells/GiantAPE1_15121_2.ome-1.tif]

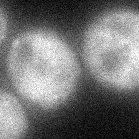

Supplement: Supplementary file 9 — Source data Fig. 5 [file 44318_2026_802_MOESM9_ESM.zip › Figure 5/Figure 5/5C/15251_NR_01_Inset_R3D.tif]

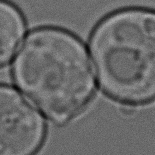

Supplement: Supplementary file 9 — Source data Fig. 5 [file 44318_2026_802_MOESM9_ESM.zip › Figure 5/Figure 5/5C/15251_NR_01_Inset_R3D_REF.tif]

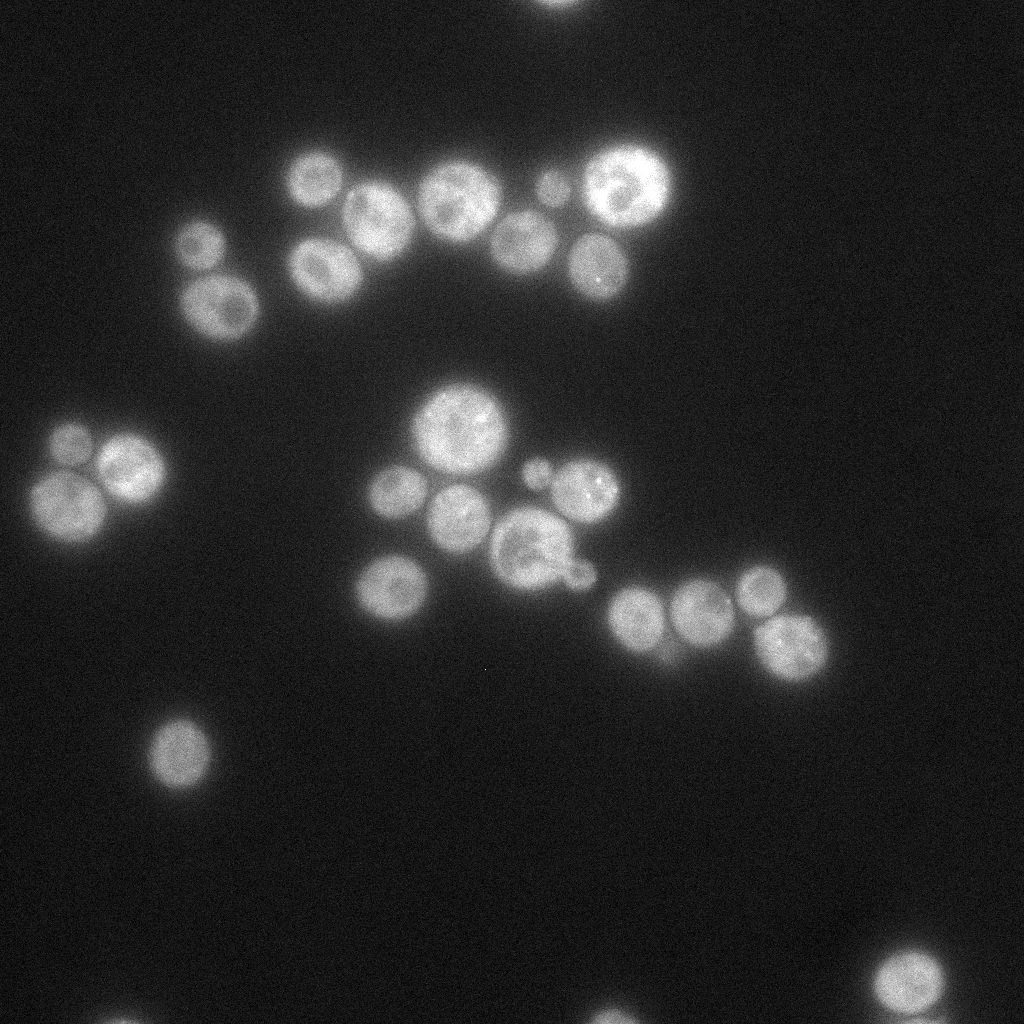

Supplement: Supplementary file 9 — Source data Fig. 5 [file 44318_2026_802_MOESM9_ESM.zip › Figure 5/Figure 5/5C/15251_NR_01_R3D.tif]

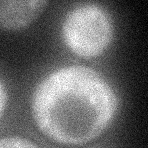

Supplement: Supplementary file 9 — Source data Fig. 5 [file 44318_2026_802_MOESM9_ESM.zip › Figure 5/Figure 5/5C/15251_ST_01_Inset_R3D.tif]

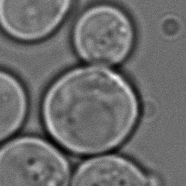

Supplement: Supplementary file 9 — Source data Fig. 5 [file 44318_2026_802_MOESM9_ESM.zip › Figure 5/Figure 5/5C/15251_ST_01_Inset_R3D_REF.tif]

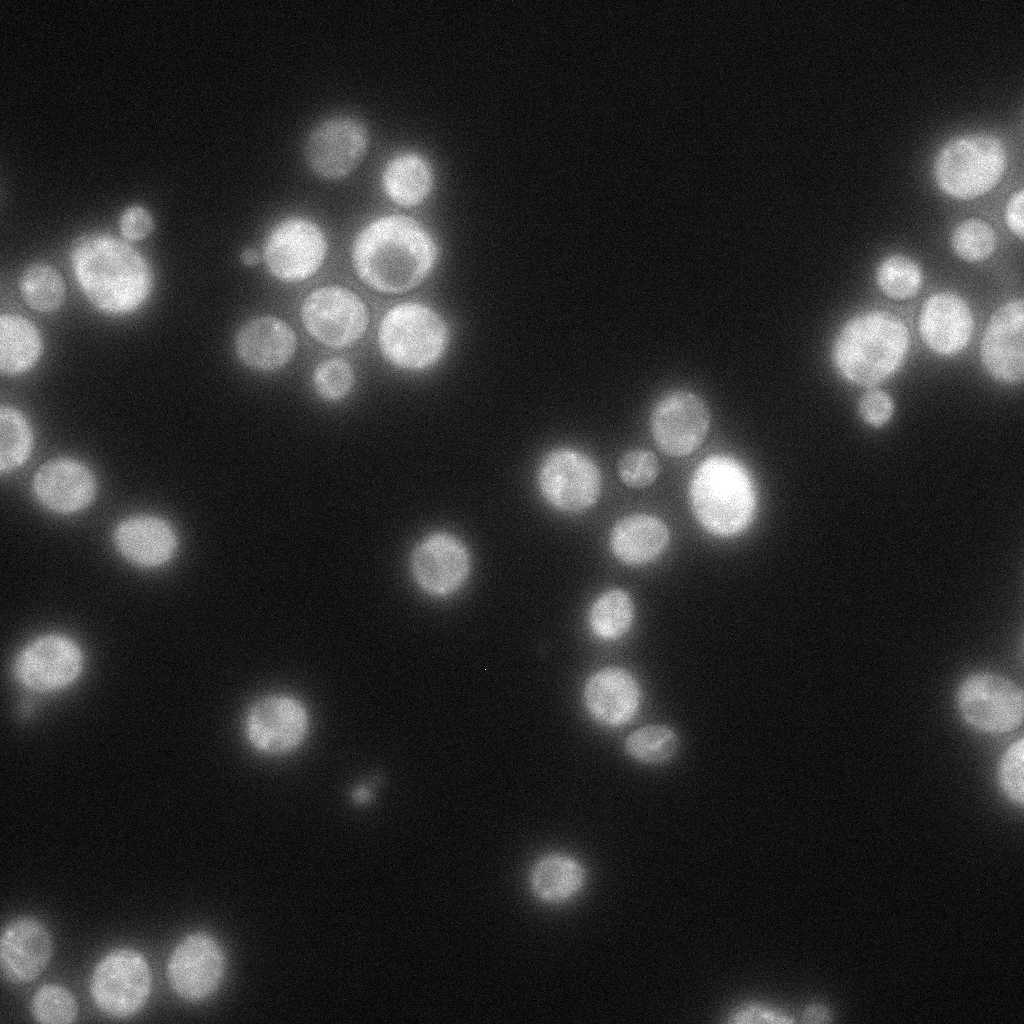

Supplement: Supplementary file 9 — Source data Fig. 5 [file 44318_2026_802_MOESM9_ESM.zip › Figure 5/Figure 5/5C/15251_ST_01_R3D.tif]

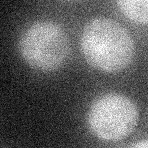

Supplement: Supplementary file 9 — Source data Fig. 5 [file 44318_2026_802_MOESM9_ESM.zip › Figure 5/Figure 5/5C/15253_NR_02_Inset_R3D.tif]

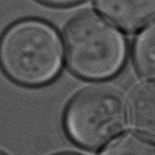

Supplement: Supplementary file 9 — Source data Fig. 5 [file 44318_2026_802_MOESM9_ESM.zip › Figure 5/Figure 5/5C/15253_NR_02_Inset_R3D_REF.tif]

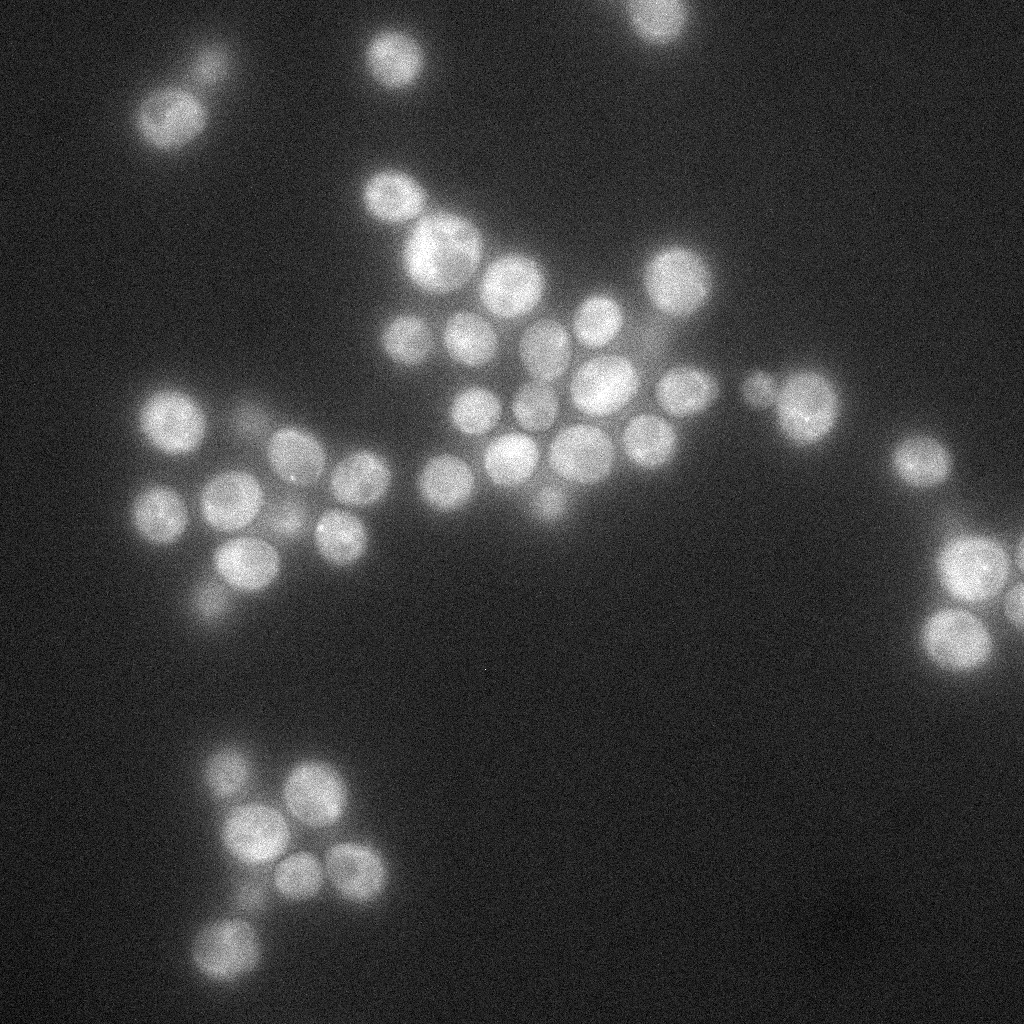

Supplement: Supplementary file 9 — Source data Fig. 5 [file 44318_2026_802_MOESM9_ESM.zip › Figure 5/Figure 5/5C/15253_NR_02_R3D.tif]

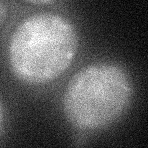

Supplement: Supplementary file 9 — Source data Fig. 5 [file 44318_2026_802_MOESM9_ESM.zip › Figure 5/Figure 5/5C/15253_ST_03_Inset_R3D.tif]

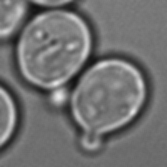

Supplement: Supplementary file 9 — Source data Fig. 5 [file 44318_2026_802_MOESM9_ESM.zip › Figure 5/Figure 5/5C/15253_ST_03_Inset_R3D_REF.tif]

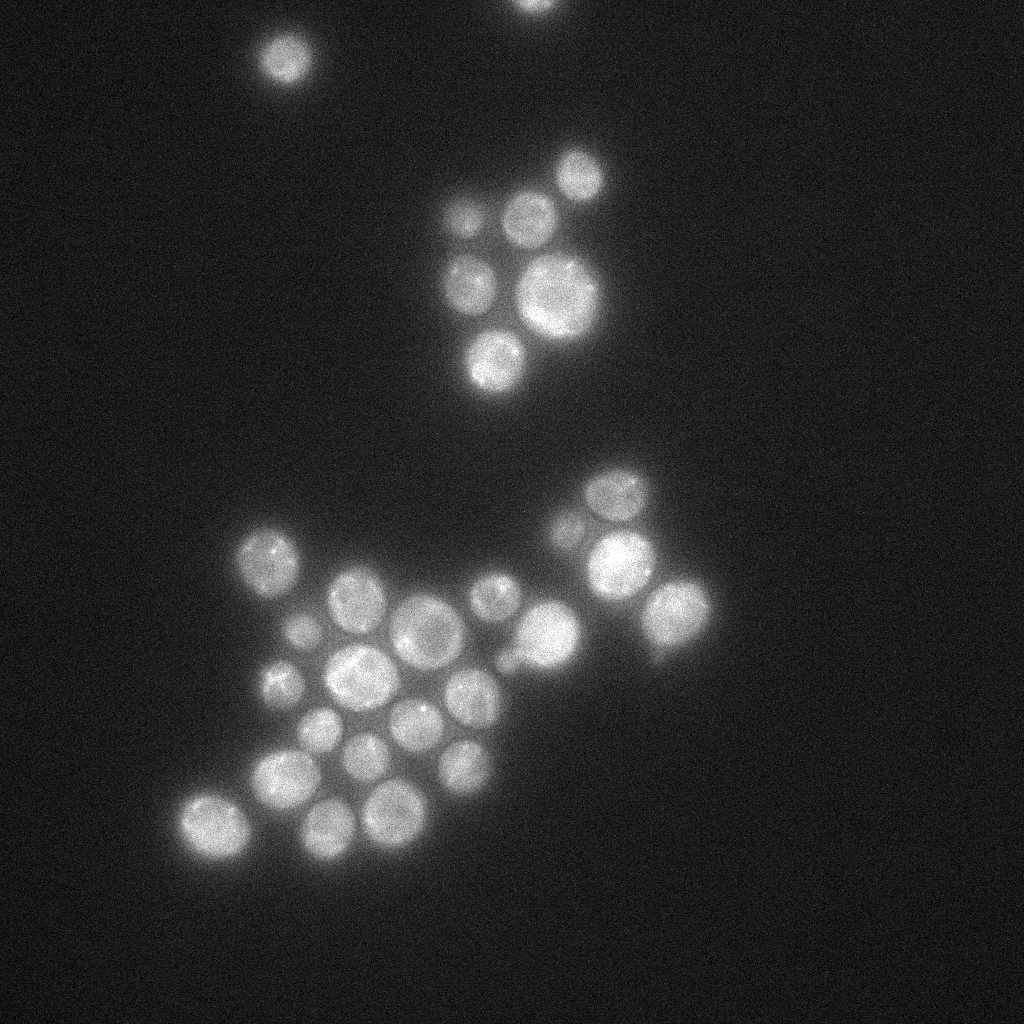

Supplement: Supplementary file 9 — Source data Fig. 5 [file 44318_2026_802_MOESM9_ESM.zip › Figure 5/Figure 5/5C/15253_ST_03_R3D.tif]

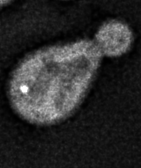

Supplement: Supplementary file 9 — Source data Fig. 5 [file 44318_2026_802_MOESM9_ESM.zip › Figure 5/Figure 5/5D-F/Analyzed cells/Dataset I/15250_01.tif]

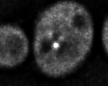

Supplement: Supplementary file 9 — Source data Fig. 5 [file 44318_2026_802_MOESM9_ESM.zip › Figure 5/Figure 5/5D-F/Analyzed cells/Dataset I/15250_02.tif]

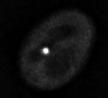

Supplement: Supplementary file 9 — Source data Fig. 5 [file 44318_2026_802_MOESM9_ESM.zip › Figure 5/Figure 5/5D-F/Analyzed cells/Dataset I/15250_03.tif]

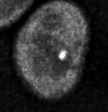

Supplement: Supplementary file 9 — Source data Fig. 5 [file 44318_2026_802_MOESM9_ESM.zip › Figure 5/Figure 5/5D-F/Analyzed cells/Dataset I/15250_04.tif]

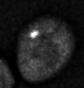

Supplement: Supplementary file 9 — Source data Fig. 5 [file 44318_2026_802_MOESM9_ESM.zip › Figure 5/Figure 5/5D-F/Analyzed cells/Dataset I/15250_05.tif]

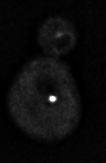

Supplement: Supplementary file 9 — Source data Fig. 5 [file 44318_2026_802_MOESM9_ESM.zip › Figure 5/Figure 5/5D-F/Analyzed cells/Dataset I/15250_06.tif]

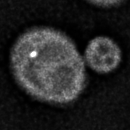

Supplement: Supplementary file 9 — Source data Fig. 5 [file 44318_2026_802_MOESM9_ESM.zip › Figure 5/Figure 5/5D-F/Analyzed cells/Dataset I/15250_07.tif]

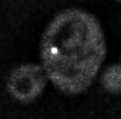

Supplement: Supplementary file 9 — Source data Fig. 5 [file 44318_2026_802_MOESM9_ESM.zip › Figure 5/Figure 5/5D-F/Analyzed cells/Dataset I/15250_08.tif]

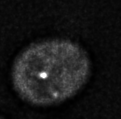

Supplement: Supplementary file 9 — Source data Fig. 5 [file 44318_2026_802_MOESM9_ESM.zip › Figure 5/Figure 5/5D-F/Analyzed cells/Dataset I/15250_09.tif]

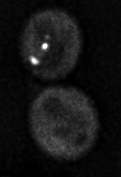

Supplement: Supplementary file 9 — Source data Fig. 5 [file 44318_2026_802_MOESM9_ESM.zip › Figure 5/Figure 5/5D-F/Analyzed cells/Dataset I/15250_10.tif]

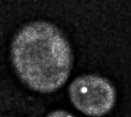

Supplement: Supplementary file 9 — Source data Fig. 5 [file 44318_2026_802_MOESM9_ESM.zip › Figure 5/Figure 5/5D-F/Analyzed cells/Dataset I/15250_11.tif]

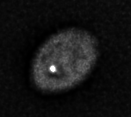

Supplement: Supplementary file 9 — Source data Fig. 5 [file 44318_2026_802_MOESM9_ESM.zip › Figure 5/Figure 5/5D-F/Analyzed cells/Dataset I/15250_12.tif]

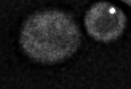

Supplement: Supplementary file 9 — Source data Fig. 5 [file 44318_2026_802_MOESM9_ESM.zip › Figure 5/Figure 5/5D-F/Analyzed cells/Dataset I/15250_13.tif]

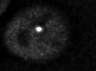

Supplement: Supplementary file 9 — Source data Fig. 5 [file 44318_2026_802_MOESM9_ESM.zip › Figure 5/Figure 5/5D-F/Analyzed cells/Dataset I/15250_14.tif]

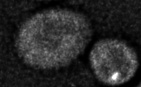

Supplement: Supplementary file 9 — Source data Fig. 5 [file 44318_2026_802_MOESM9_ESM.zip › Figure 5/Figure 5/5D-F/Analyzed cells/Dataset I/15250_15.tif]

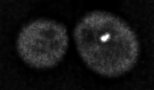

Supplement: Supplementary file 9 — Source data Fig. 5 [file 44318_2026_802_MOESM9_ESM.zip › Figure 5/Figure 5/5D-F/Analyzed cells/Dataset I/15250_16.tif]

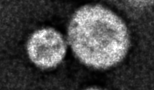

Supplement: Supplementary file 9 — Source data Fig. 5 [file 44318_2026_802_MOESM9_ESM.zip › Figure 5/Figure 5/5D-F/Analyzed cells/Dataset I/15250_17.tif]

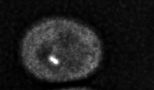

Supplement: Supplementary file 9 — Source data Fig. 5 [file 44318_2026_802_MOESM9_ESM.zip › Figure 5/Figure 5/5D-F/Analyzed cells/Dataset I/15250_18.tif]

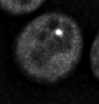

Supplement: Supplementary file 9 — Source data Fig. 5 [file 44318_2026_802_MOESM9_ESM.zip › Figure 5/Figure 5/5D-F/Analyzed cells/Dataset I/15250_19.tif]

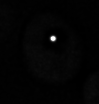

Supplement: Supplementary file 9 — Source data Fig. 5 [file 44318_2026_802_MOESM9_ESM.zip › Figure 5/Figure 5/5D-F/Analyzed cells/Dataset I/15250_20.tif]
