# Supplementary figures and images for: Atg18 interaction positions Atg2 for efficient lipid transfer into phagophore elongation (part 3 of 3)
Source: EMBO J. 2026 May 20;45(12):4034–60. doi: 10.1038/s44318-026-00802-3 (PMC13269710; doi:10.1038/s44318-026-00802-3)

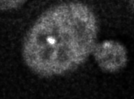

Supplement: Supplementary file 9 — Source data Fig. 5 [file 44318_2026_802_MOESM9_ESM.zip › Figure 5/Figure 5/5D-F/Analyzed cells/Dataset III/15250_02.tif]

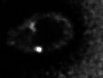

Supplement: Supplementary file 9 — Source data Fig. 5 [file 44318_2026_802_MOESM9_ESM.zip › Figure 5/Figure 5/5D-F/Analyzed cells/Dataset III/15250_03.tif]

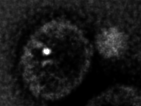

Supplement: Supplementary file 9 — Source data Fig. 5 [file 44318_2026_802_MOESM9_ESM.zip › Figure 5/Figure 5/5D-F/Analyzed cells/Dataset III/15250_04.tif]

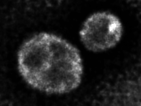

Supplement: Supplementary file 9 — Source data Fig. 5 [file 44318_2026_802_MOESM9_ESM.zip › Figure 5/Figure 5/5D-F/Analyzed cells/Dataset III/15250_05.tif]

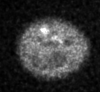

Supplement: Supplementary file 9 — Source data Fig. 5 [file 44318_2026_802_MOESM9_ESM.zip › Figure 5/Figure 5/5D-F/Analyzed cells/Dataset III/15250_06.tif]

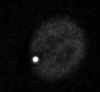

Supplement: Supplementary file 9 — Source data Fig. 5 [file 44318_2026_802_MOESM9_ESM.zip › Figure 5/Figure 5/5D-F/Analyzed cells/Dataset III/15250_07.tif]

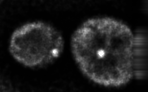

Supplement: Supplementary file 9 — Source data Fig. 5 [file 44318_2026_802_MOESM9_ESM.zip › Figure 5/Figure 5/5D-F/Analyzed cells/Dataset III/15250_08.tif]

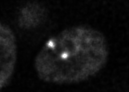

Supplement: Supplementary file 9 — Source data Fig. 5 [file 44318_2026_802_MOESM9_ESM.zip › Figure 5/Figure 5/5D-F/Analyzed cells/Dataset III/15250_09.tif]

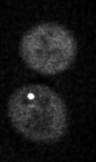

Supplement: Supplementary file 9 — Source data Fig. 5 [file 44318_2026_802_MOESM9_ESM.zip › Figure 5/Figure 5/5D-F/Analyzed cells/Dataset III/15250_10.tif]

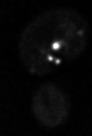

Supplement: Supplementary file 9 — Source data Fig. 5 [file 44318_2026_802_MOESM9_ESM.zip › Figure 5/Figure 5/5D-F/Analyzed cells/Dataset III/15250_11.tif]

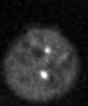

Supplement: Supplementary file 9 — Source data Fig. 5 [file 44318_2026_802_MOESM9_ESM.zip › Figure 5/Figure 5/5D-F/Analyzed cells/Dataset III/15251_01.tif]

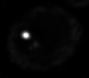

Supplement: Supplementary file 9 — Source data Fig. 5 [file 44318_2026_802_MOESM9_ESM.zip › Figure 5/Figure 5/5D-F/Analyzed cells/Dataset III/15251_02.tif]

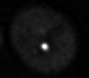

Supplement: Supplementary file 9 — Source data Fig. 5 [file 44318_2026_802_MOESM9_ESM.zip › Figure 5/Figure 5/5D-F/Analyzed cells/Dataset III/15251_03.tif]

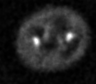

Supplement: Supplementary file 9 — Source data Fig. 5 [file 44318_2026_802_MOESM9_ESM.zip › Figure 5/Figure 5/5D-F/Analyzed cells/Dataset III/15251_04.tif]

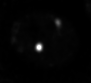

Supplement: Supplementary file 9 — Source data Fig. 5 [file 44318_2026_802_MOESM9_ESM.zip › Figure 5/Figure 5/5D-F/Analyzed cells/Dataset III/15251_05.tif]

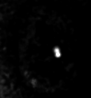

Supplement: Supplementary file 9 — Source data Fig. 5 [file 44318_2026_802_MOESM9_ESM.zip › Figure 5/Figure 5/5D-F/Analyzed cells/Dataset III/15251_06.tif]

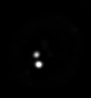

Supplement: Supplementary file 9 — Source data Fig. 5 [file 44318_2026_802_MOESM9_ESM.zip › Figure 5/Figure 5/5D-F/Analyzed cells/Dataset III/15251_07.tif]

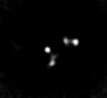

Supplement: Supplementary file 9 — Source data Fig. 5 [file 44318_2026_802_MOESM9_ESM.zip › Figure 5/Figure 5/5D-F/Analyzed cells/Dataset III/15251_08.tif]

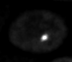

Supplement: Supplementary file 9 — Source data Fig. 5 [file 44318_2026_802_MOESM9_ESM.zip › Figure 5/Figure 5/5D-F/Analyzed cells/Dataset III/15251_09.tif]

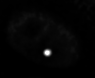

Supplement: Supplementary file 9 — Source data Fig. 5 [file 44318_2026_802_MOESM9_ESM.zip › Figure 5/Figure 5/5D-F/Analyzed cells/Dataset III/15251_10.tif]

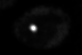

Supplement: Supplementary file 9 — Source data Fig. 5 [file 44318_2026_802_MOESM9_ESM.zip › Figure 5/Figure 5/5D-F/Analyzed cells/Dataset III/15251_11.tif]

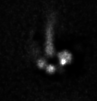

Supplement: Supplementary file 9 — Source data Fig. 5 [file 44318_2026_802_MOESM9_ESM.zip › Figure 5/Figure 5/5D-F/Analyzed cells/Dataset III/15253_01.tif]

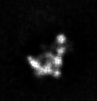

Supplement: Supplementary file 9 — Source data Fig. 5 [file 44318_2026_802_MOESM9_ESM.zip › Figure 5/Figure 5/5D-F/Analyzed cells/Dataset III/15253_02.tif]

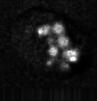

Supplement: Supplementary file 9 — Source data Fig. 5 [file 44318_2026_802_MOESM9_ESM.zip › Figure 5/Figure 5/5D-F/Analyzed cells/Dataset III/15253_03.tif]

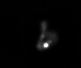

Supplement: Supplementary file 9 — Source data Fig. 5 [file 44318_2026_802_MOESM9_ESM.zip › Figure 5/Figure 5/5D-F/Analyzed cells/Dataset III/15253_04.tif]

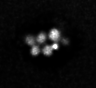

Supplement: Supplementary file 9 — Source data Fig. 5 [file 44318_2026_802_MOESM9_ESM.zip › Figure 5/Figure 5/5D-F/Analyzed cells/Dataset III/15253_05.tif]

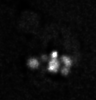

Supplement: Supplementary file 9 — Source data Fig. 5 [file 44318_2026_802_MOESM9_ESM.zip › Figure 5/Figure 5/5D-F/Analyzed cells/Dataset III/15253_06.tif]

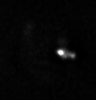

Supplement: Supplementary file 9 — Source data Fig. 5 [file 44318_2026_802_MOESM9_ESM.zip › Figure 5/Figure 5/5D-F/Analyzed cells/Dataset III/15253_07.tif]

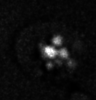

Supplement: Supplementary file 9 — Source data Fig. 5 [file 44318_2026_802_MOESM9_ESM.zip › Figure 5/Figure 5/5D-F/Analyzed cells/Dataset III/15253_08.tif]

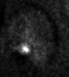

Supplement: Supplementary file 9 — Source data Fig. 5 [file 44318_2026_802_MOESM9_ESM.zip › Figure 5/Figure 5/5D-F/Analyzed cells/Dataset III/15253_09.tif]

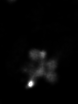

Supplement: Supplementary file 9 — Source data Fig. 5 [file 44318_2026_802_MOESM9_ESM.zip › Figure 5/Figure 5/5D-F/Analyzed cells/Dataset III/15253_10.tif]

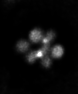

Supplement: Supplementary file 9 — Source data Fig. 5 [file 44318_2026_802_MOESM9_ESM.zip › Figure 5/Figure 5/5D-F/Analyzed cells/Dataset III/15253_11.tif]

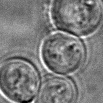

Supplement: Supplementary file 10 — Source data Fig. 6 [file 44318_2026_802_MOESM10_ESM.zip › Figure 6/6A/PVY122-5-inset-REF.tif]

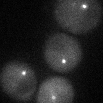

Supplement: Supplementary file 10 — Source data Fig. 6 [file 44318_2026_802_MOESM10_ESM.zip › Figure 6/6A/PVY122-5-inset.tif]

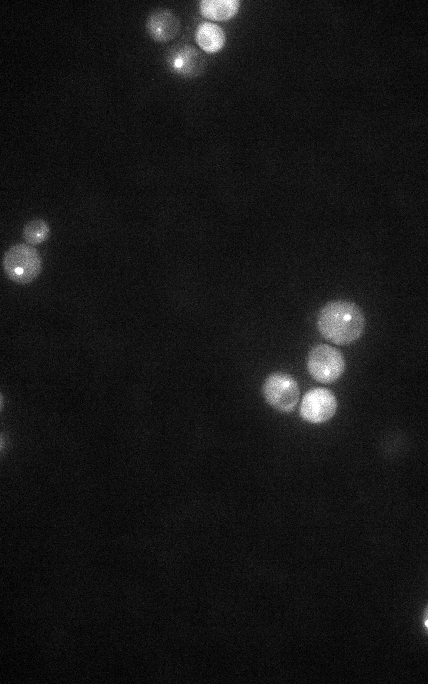

Supplement: Supplementary file 10 — Source data Fig. 6 [file 44318_2026_802_MOESM10_ESM.zip › Figure 6/6A/PVY122-5.tif]

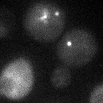

Supplement: Supplementary file 10 — Source data Fig. 6 [file 44318_2026_802_MOESM10_ESM.zip › Figure 6/6A/RGY541-6 inset.tif]

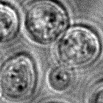

Supplement: Supplementary file 10 — Source data Fig. 6 [file 44318_2026_802_MOESM10_ESM.zip › Figure 6/6A/RGY541-6-inset-REF.tif]

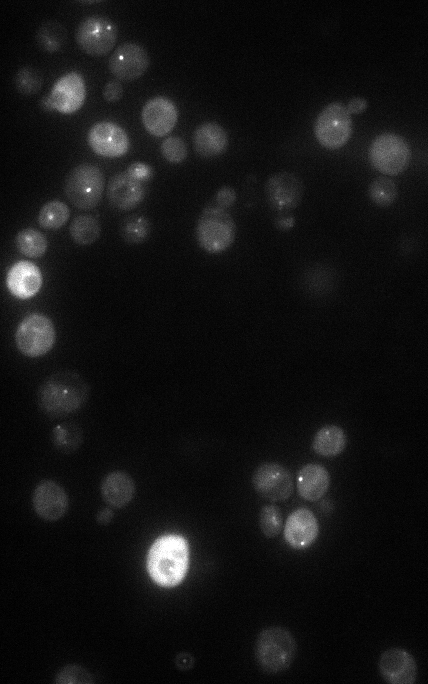

Supplement: Supplementary file 10 — Source data Fig. 6 [file 44318_2026_802_MOESM10_ESM.zip › Figure 6/6A/RGY541-6.tif]

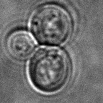

Supplement: Supplementary file 10 — Source data Fig. 6 [file 44318_2026_802_MOESM10_ESM.zip › Figure 6/6A/RGY637-2-inset-REF.tif]

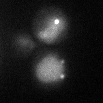

Supplement: Supplementary file 10 — Source data Fig. 6 [file 44318_2026_802_MOESM10_ESM.zip › Figure 6/6A/RGY637-2-inset.tif]

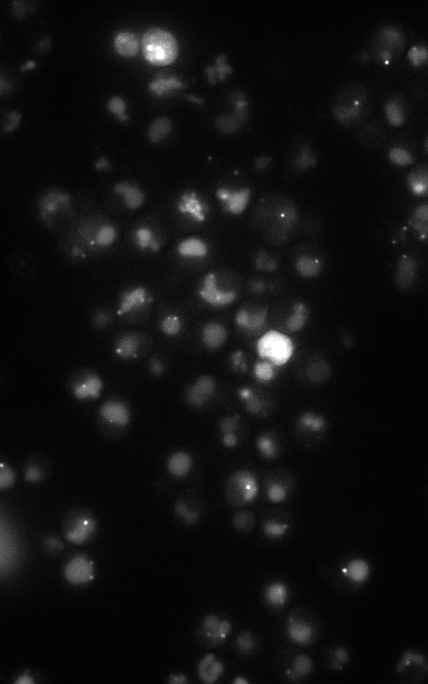

Supplement: Supplementary file 10 — Source data Fig. 6 [file 44318_2026_802_MOESM10_ESM.zip › Figure 6/6A/RGY637-2.tif]

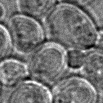

Supplement: Supplementary file 10 — Source data Fig. 6 [file 44318_2026_802_MOESM10_ESM.zip › Figure 6/6C/PVY125-3 inset-REF.tif]

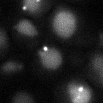

Supplement: Supplementary file 10 — Source data Fig. 6 [file 44318_2026_802_MOESM10_ESM.zip › Figure 6/6C/PVY125-3 inset.tif]

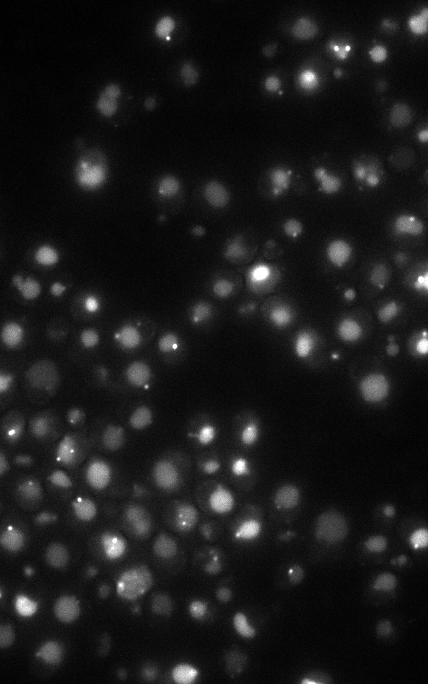

Supplement: Supplementary file 10 — Source data Fig. 6 [file 44318_2026_802_MOESM10_ESM.zip › Figure 6/6C/PVY125-3.tif]

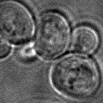

Supplement: Supplementary file 10 — Source data Fig. 6 [file 44318_2026_802_MOESM10_ESM.zip › Figure 6/6C/PVY126 inset-REF.tif]

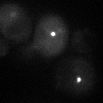

Supplement: Supplementary file 10 — Source data Fig. 6 [file 44318_2026_802_MOESM10_ESM.zip › Figure 6/6C/PVY126 inset.tif]

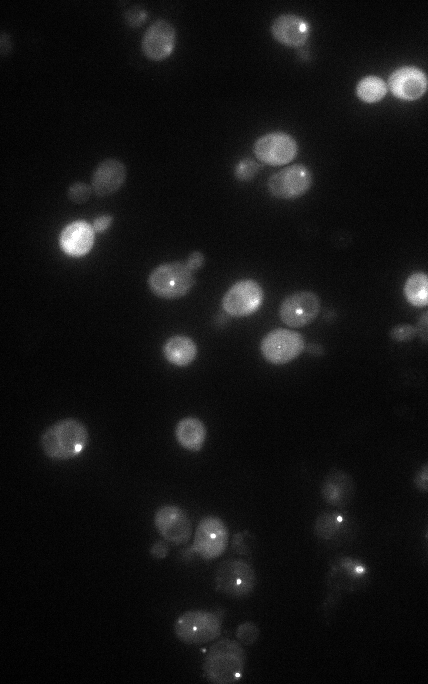

Supplement: Supplementary file 10 — Source data Fig. 6 [file 44318_2026_802_MOESM10_ESM.zip › Figure 6/6C/PVY126.tif]

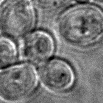

Supplement: Supplementary file 10 — Source data Fig. 6 [file 44318_2026_802_MOESM10_ESM.zip › Figure 6/6C/RGY1015-7 inset-REF.tif]

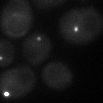

Supplement: Supplementary file 10 — Source data Fig. 6 [file 44318_2026_802_MOESM10_ESM.zip › Figure 6/6C/RGY1015-7 inset.tif]

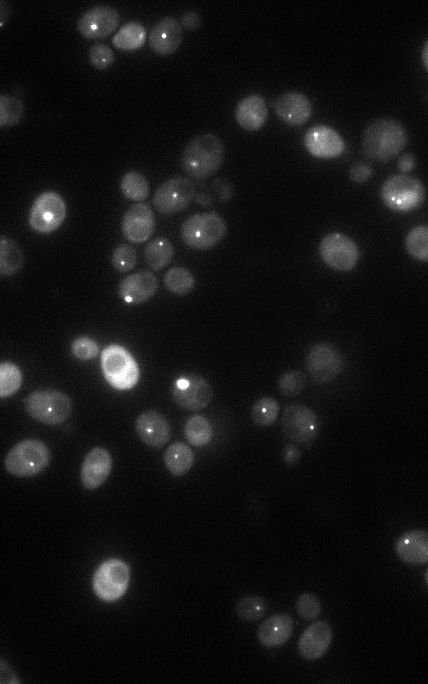

Supplement: Supplementary file 10 — Source data Fig. 6 [file 44318_2026_802_MOESM10_ESM.zip › Figure 6/6C/RGY1015-7.tif]

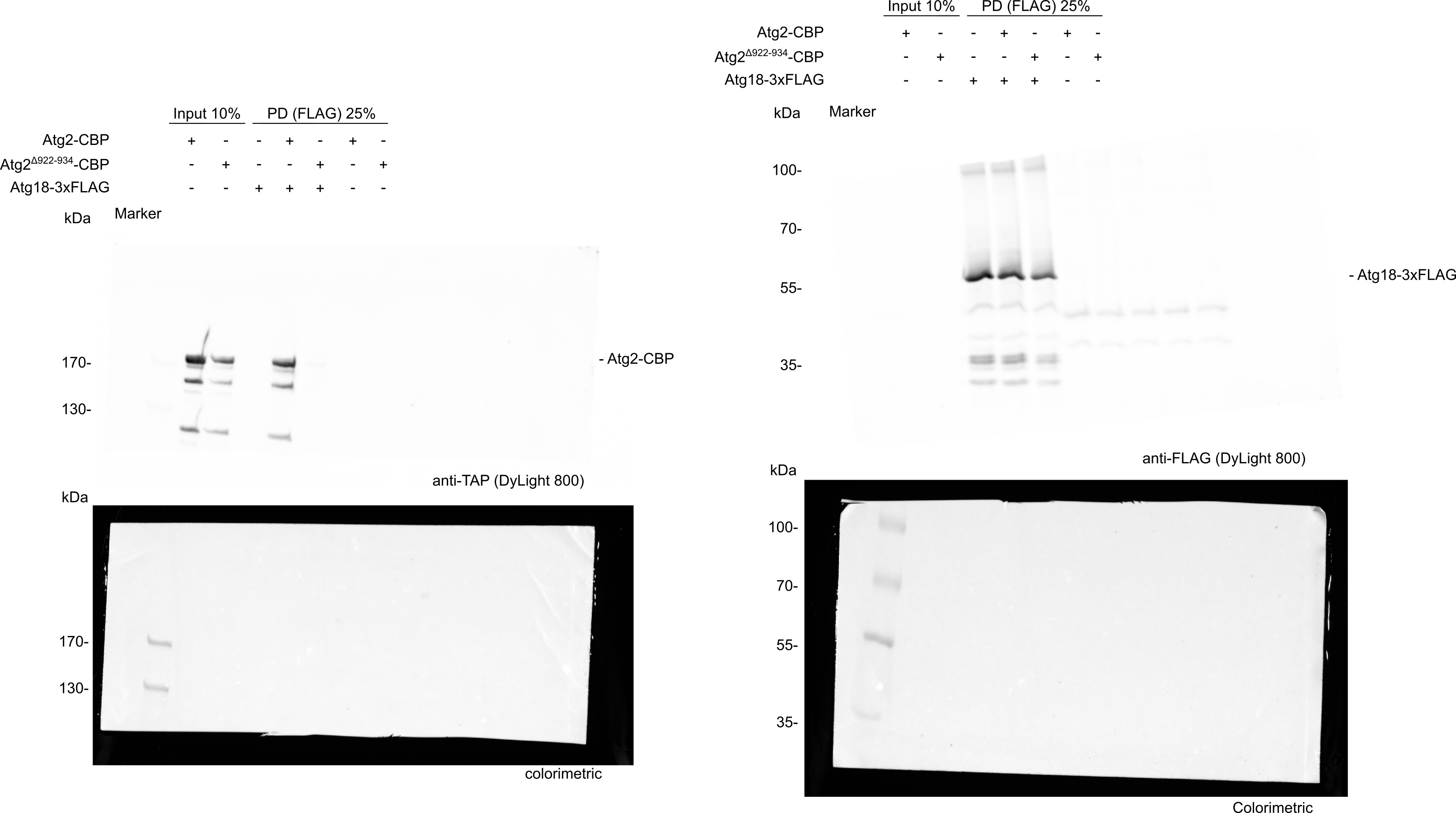

Supplement: Supplementary file 11 — Source data Fig. 7 [file 44318_2026_802_MOESM11_ESM.zip › Figure 7/7D/Pull-down Atg2-Atg18_Replicate 2.tiff]

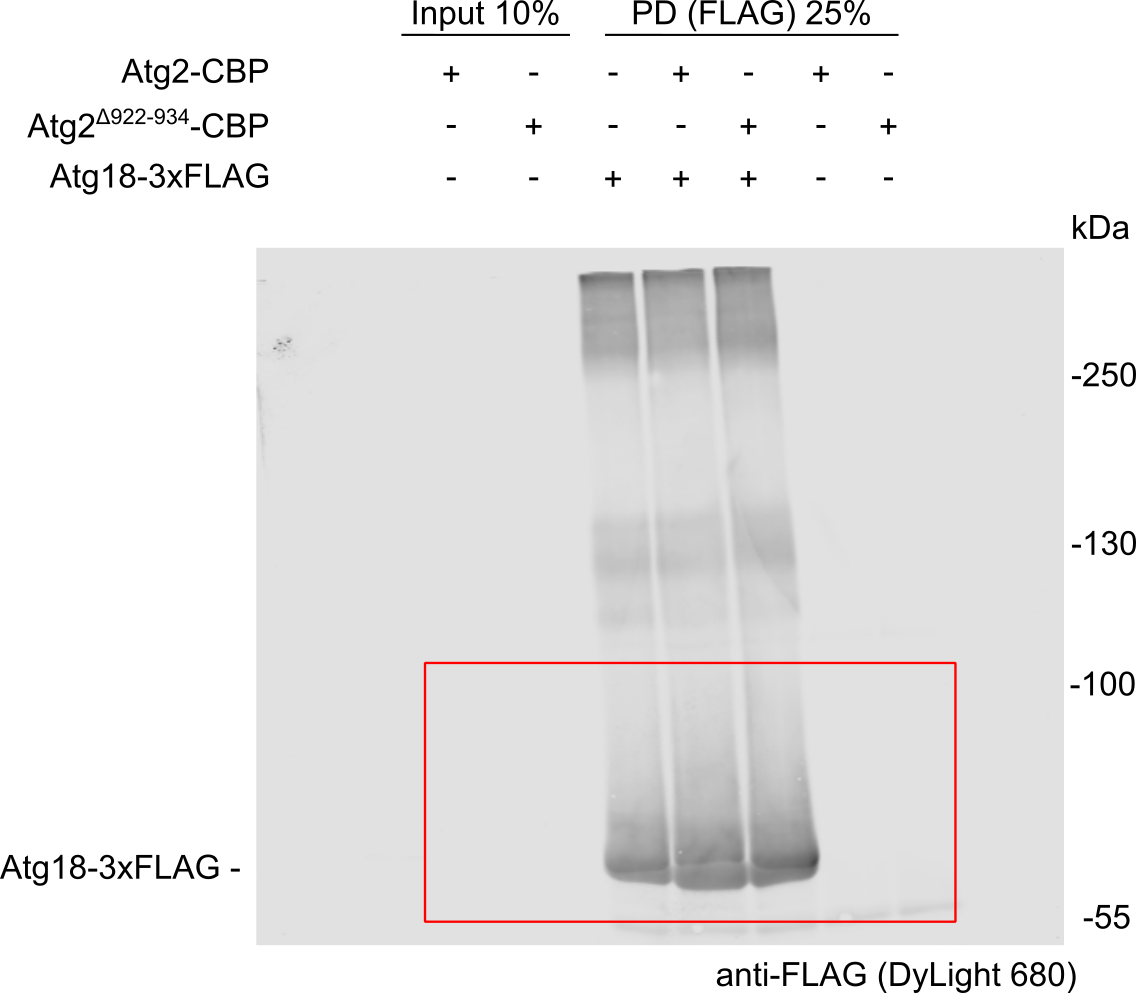

Supplement: Supplementary file 11 — Source data Fig. 7 [file 44318_2026_802_MOESM11_ESM.zip › Figure 7/7D/Pull-down Atg2-Atg18_anti-FLAG.tiff]

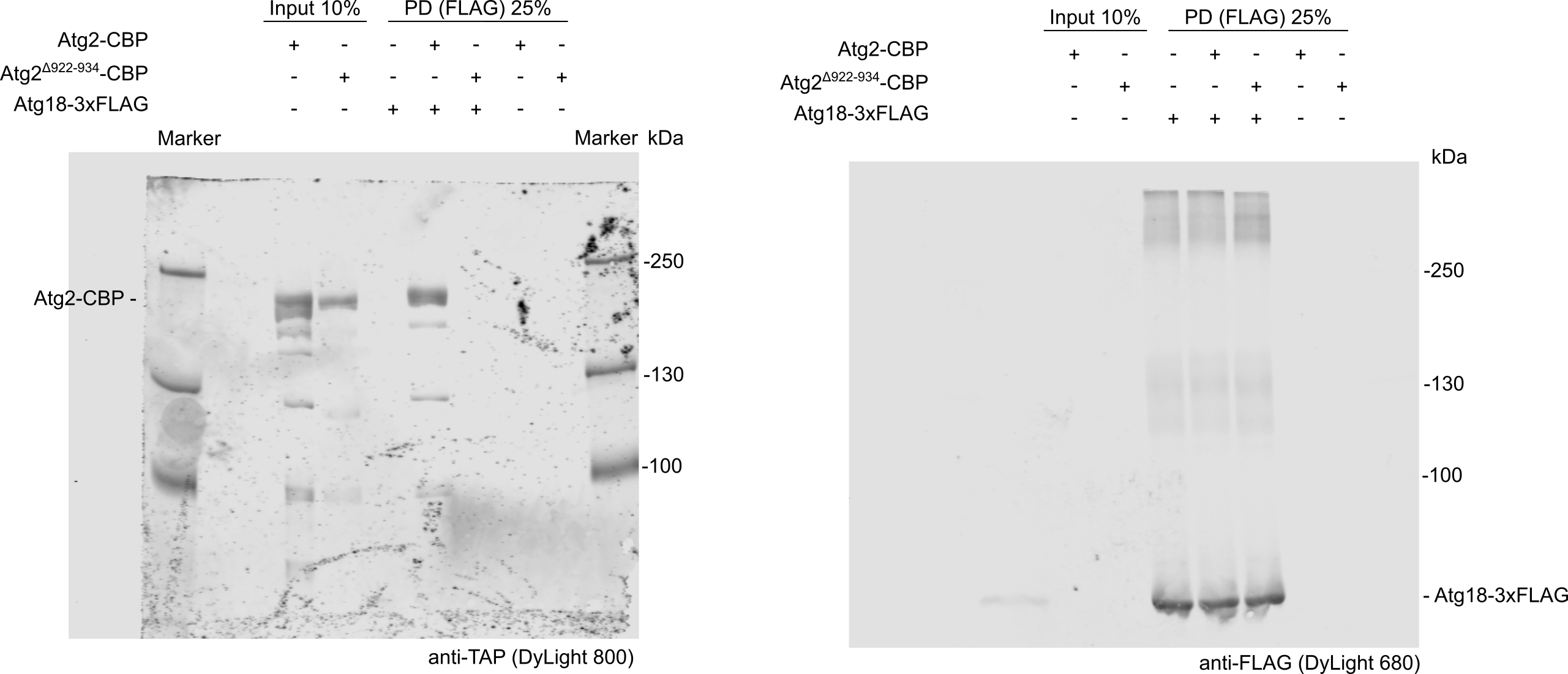

Supplement: Supplementary file 11 — Source data Fig. 7 [file 44318_2026_802_MOESM11_ESM.zip › Figure 7/7D/Pull-down Atg2-Atg18_Replicate 3.tiff]

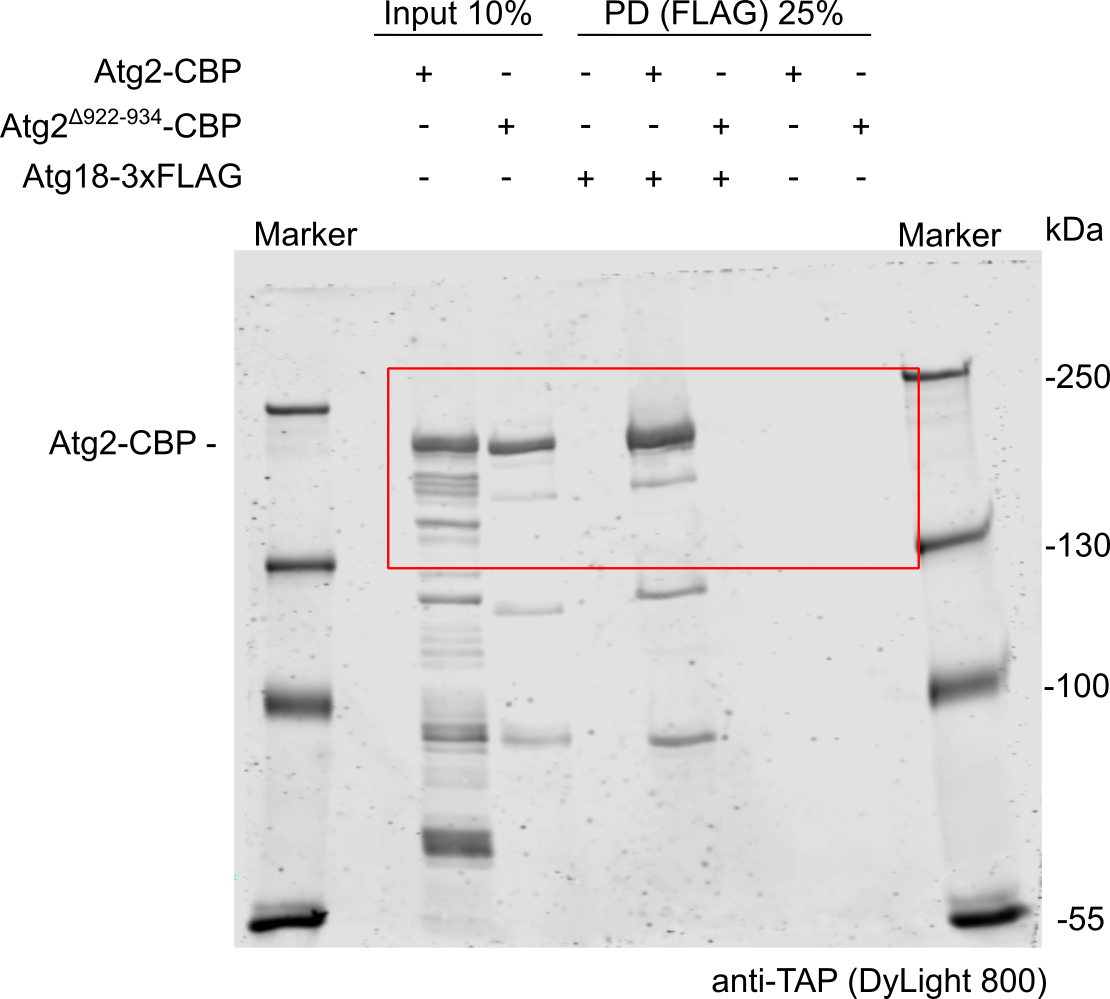

Supplement: Supplementary file 11 — Source data Fig. 7 [file 44318_2026_802_MOESM11_ESM.zip › Figure 7/7D/Pull-down Atg2-Atg18_anti-TAP.tiff]

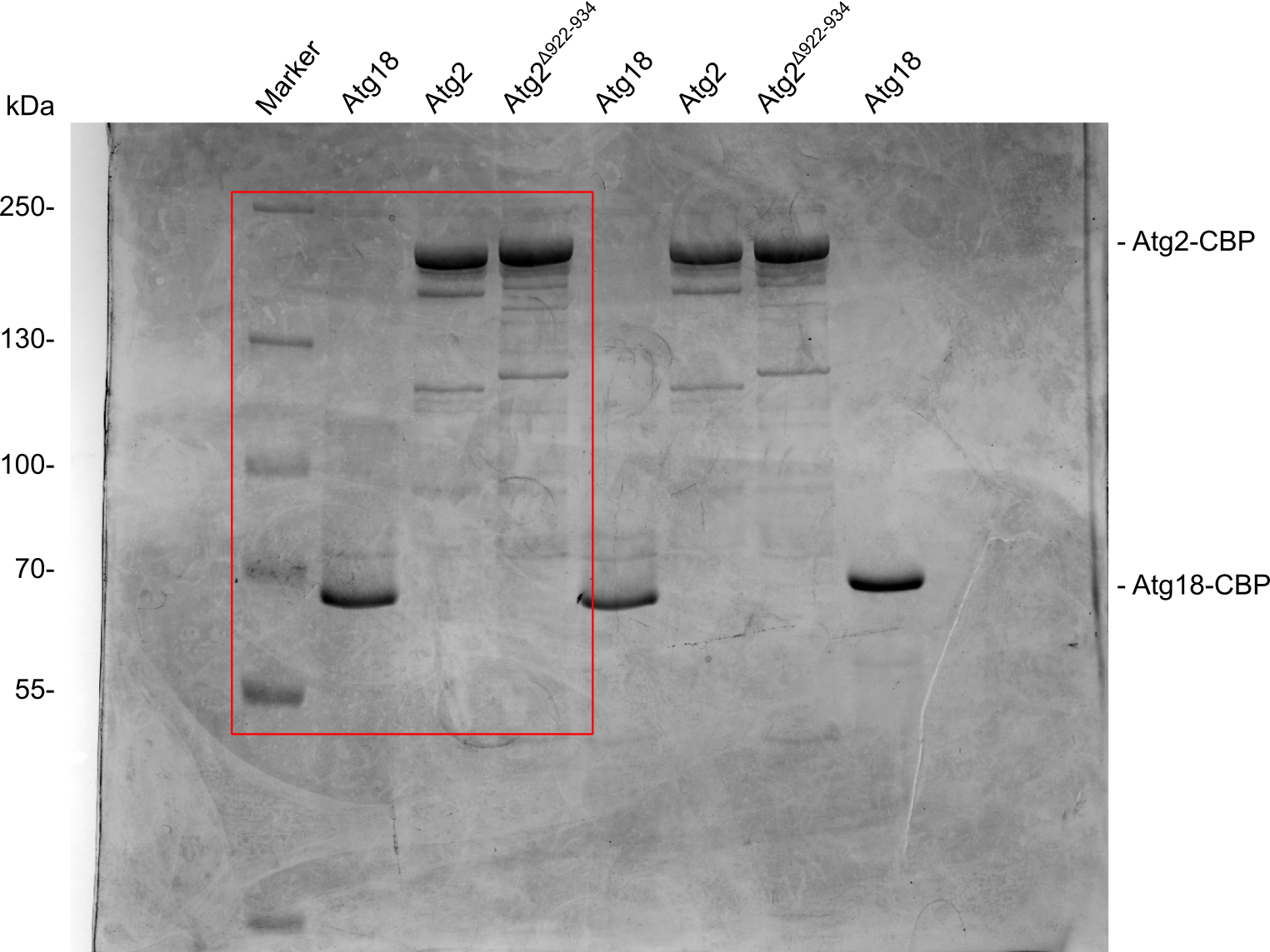

Supplement: Supplementary file 11 — Source data Fig. 7 [file 44318_2026_802_MOESM11_ESM.zip › Figure 7/7A/SDS-PAGE-Coomassie-purified Atg2 and Atg18.tiff]
