# Supplementary figures and images for: Atg18 interaction positions Atg2 for efficient lipid transfer into phagophore elongation (part 2 of 3)
Source: EMBO J. 2026 May 20;45(12):4034–60. doi: 10.1038/s44318-026-00802-3 (PMC13269710; doi:10.1038/s44318-026-00802-3)

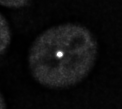

Supplement: Supplementary file 9 — Source data Fig. 5 [file 44318_2026_802_MOESM9_ESM.zip › Figure 5/Figure 5/5D-F/Analyzed cells/Dataset I/15251_01.tif]

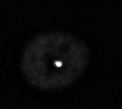

Supplement: Supplementary file 9 — Source data Fig. 5 [file 44318_2026_802_MOESM9_ESM.zip › Figure 5/Figure 5/5D-F/Analyzed cells/Dataset I/15251_02.tif]

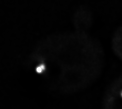

Supplement: Supplementary file 9 — Source data Fig. 5 [file 44318_2026_802_MOESM9_ESM.zip › Figure 5/Figure 5/5D-F/Analyzed cells/Dataset I/15251_03.tif]

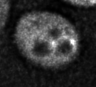

Supplement: Supplementary file 9 — Source data Fig. 5 [file 44318_2026_802_MOESM9_ESM.zip › Figure 5/Figure 5/5D-F/Analyzed cells/Dataset I/15251_04.tif]

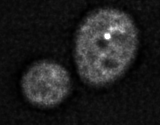

Supplement: Supplementary file 9 — Source data Fig. 5 [file 44318_2026_802_MOESM9_ESM.zip › Figure 5/Figure 5/5D-F/Analyzed cells/Dataset I/15251_05.tif]

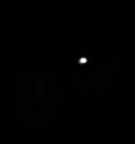

Supplement: Supplementary file 9 — Source data Fig. 5 [file 44318_2026_802_MOESM9_ESM.zip › Figure 5/Figure 5/5D-F/Analyzed cells/Dataset I/15251_06.tif]

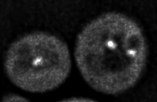

Supplement: Supplementary file 9 — Source data Fig. 5 [file 44318_2026_802_MOESM9_ESM.zip › Figure 5/Figure 5/5D-F/Analyzed cells/Dataset I/15251_07.tif]

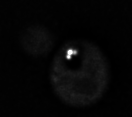

Supplement: Supplementary file 9 — Source data Fig. 5 [file 44318_2026_802_MOESM9_ESM.zip › Figure 5/Figure 5/5D-F/Analyzed cells/Dataset I/15251_08.tif]

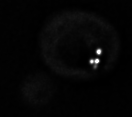

Supplement: Supplementary file 9 — Source data Fig. 5 [file 44318_2026_802_MOESM9_ESM.zip › Figure 5/Figure 5/5D-F/Analyzed cells/Dataset I/15251_09.tif]

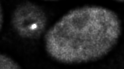

Supplement: Supplementary file 9 — Source data Fig. 5 [file 44318_2026_802_MOESM9_ESM.zip › Figure 5/Figure 5/5D-F/Analyzed cells/Dataset I/15251_10.tif]

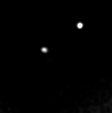

Supplement: Supplementary file 9 — Source data Fig. 5 [file 44318_2026_802_MOESM9_ESM.zip › Figure 5/Figure 5/5D-F/Analyzed cells/Dataset I/15251_11.tif]

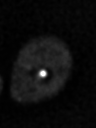

Supplement: Supplementary file 9 — Source data Fig. 5 [file 44318_2026_802_MOESM9_ESM.zip › Figure 5/Figure 5/5D-F/Analyzed cells/Dataset I/15251_12.tif]

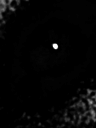

Supplement: Supplementary file 9 — Source data Fig. 5 [file 44318_2026_802_MOESM9_ESM.zip › Figure 5/Figure 5/5D-F/Analyzed cells/Dataset I/15251_13.tif]

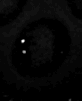

Supplement: Supplementary file 9 — Source data Fig. 5 [file 44318_2026_802_MOESM9_ESM.zip › Figure 5/Figure 5/5D-F/Analyzed cells/Dataset I/15251_14.tif]

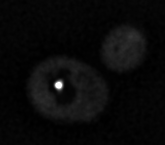

Supplement: Supplementary file 9 — Source data Fig. 5 [file 44318_2026_802_MOESM9_ESM.zip › Figure 5/Figure 5/5D-F/Analyzed cells/Dataset I/15251_15.tif]

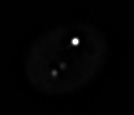

Supplement: Supplementary file 9 — Source data Fig. 5 [file 44318_2026_802_MOESM9_ESM.zip › Figure 5/Figure 5/5D-F/Analyzed cells/Dataset I/15251_16.tif]

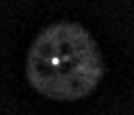

Supplement: Supplementary file 9 — Source data Fig. 5 [file 44318_2026_802_MOESM9_ESM.zip › Figure 5/Figure 5/5D-F/Analyzed cells/Dataset I/15251_17.tif]

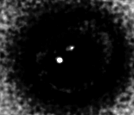

Supplement: Supplementary file 9 — Source data Fig. 5 [file 44318_2026_802_MOESM9_ESM.zip › Figure 5/Figure 5/5D-F/Analyzed cells/Dataset I/15251_18.tif]

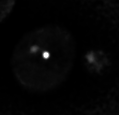

Supplement: Supplementary file 9 — Source data Fig. 5 [file 44318_2026_802_MOESM9_ESM.zip › Figure 5/Figure 5/5D-F/Analyzed cells/Dataset I/15251_19.tif]

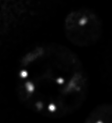

Supplement: Supplementary file 9 — Source data Fig. 5 [file 44318_2026_802_MOESM9_ESM.zip › Figure 5/Figure 5/5D-F/Analyzed cells/Dataset I/15251_20.tif]

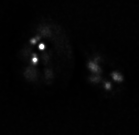

Supplement: Supplementary file 9 — Source data Fig. 5 [file 44318_2026_802_MOESM9_ESM.zip › Figure 5/Figure 5/5D-F/Analyzed cells/Dataset I/15253_01.tif]

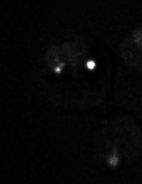

Supplement: Supplementary file 9 — Source data Fig. 5 [file 44318_2026_802_MOESM9_ESM.zip › Figure 5/Figure 5/5D-F/Analyzed cells/Dataset I/15253_02.tif]

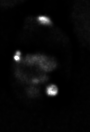

Supplement: Supplementary file 9 — Source data Fig. 5 [file 44318_2026_802_MOESM9_ESM.zip › Figure 5/Figure 5/5D-F/Analyzed cells/Dataset I/15253_03.tif]

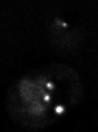

Supplement: Supplementary file 9 — Source data Fig. 5 [file 44318_2026_802_MOESM9_ESM.zip › Figure 5/Figure 5/5D-F/Analyzed cells/Dataset I/15253_04.tif]

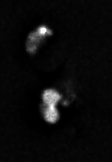

Supplement: Supplementary file 9 — Source data Fig. 5 [file 44318_2026_802_MOESM9_ESM.zip › Figure 5/Figure 5/5D-F/Analyzed cells/Dataset I/15253_05.tif]

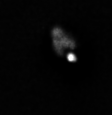

Supplement: Supplementary file 9 — Source data Fig. 5 [file 44318_2026_802_MOESM9_ESM.zip › Figure 5/Figure 5/5D-F/Analyzed cells/Dataset I/15253_06.tif]

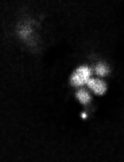

Supplement: Supplementary file 9 — Source data Fig. 5 [file 44318_2026_802_MOESM9_ESM.zip › Figure 5/Figure 5/5D-F/Analyzed cells/Dataset I/15253_07.tif]

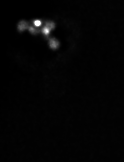

Supplement: Supplementary file 9 — Source data Fig. 5 [file 44318_2026_802_MOESM9_ESM.zip › Figure 5/Figure 5/5D-F/Analyzed cells/Dataset I/15253_08.tif]

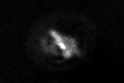

Supplement: Supplementary file 9 — Source data Fig. 5 [file 44318_2026_802_MOESM9_ESM.zip › Figure 5/Figure 5/5D-F/Analyzed cells/Dataset I/15253_09.tif]

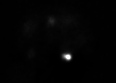

Supplement: Supplementary file 9 — Source data Fig. 5 [file 44318_2026_802_MOESM9_ESM.zip › Figure 5/Figure 5/5D-F/Analyzed cells/Dataset I/15253_10.tif]

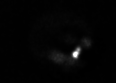

Supplement: Supplementary file 9 — Source data Fig. 5 [file 44318_2026_802_MOESM9_ESM.zip › Figure 5/Figure 5/5D-F/Analyzed cells/Dataset I/15253_11.tif]

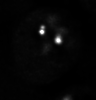

Supplement: Supplementary file 9 — Source data Fig. 5 [file 44318_2026_802_MOESM9_ESM.zip › Figure 5/Figure 5/5D-F/Analyzed cells/Dataset I/15253_12.tif]

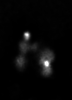

Supplement: Supplementary file 9 — Source data Fig. 5 [file 44318_2026_802_MOESM9_ESM.zip › Figure 5/Figure 5/5D-F/Analyzed cells/Dataset I/15253_13.tif]

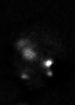

Supplement: Supplementary file 9 — Source data Fig. 5 [file 44318_2026_802_MOESM9_ESM.zip › Figure 5/Figure 5/5D-F/Analyzed cells/Dataset I/15253_14.tif]

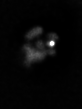

Supplement: Supplementary file 9 — Source data Fig. 5 [file 44318_2026_802_MOESM9_ESM.zip › Figure 5/Figure 5/5D-F/Analyzed cells/Dataset I/15253_16.tif]

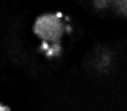

Supplement: Supplementary file 9 — Source data Fig. 5 [file 44318_2026_802_MOESM9_ESM.zip › Figure 5/Figure 5/5D-F/Analyzed cells/Dataset I/15253_17.tif]

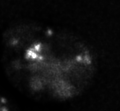

Supplement: Supplementary file 9 — Source data Fig. 5 [file 44318_2026_802_MOESM9_ESM.zip › Figure 5/Figure 5/5D-F/Analyzed cells/Dataset I/15253_18.tif]

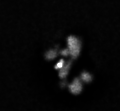

Supplement: Supplementary file 9 — Source data Fig. 5 [file 44318_2026_802_MOESM9_ESM.zip › Figure 5/Figure 5/5D-F/Analyzed cells/Dataset I/15253_19.tif]

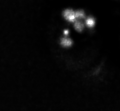

Supplement: Supplementary file 9 — Source data Fig. 5 [file 44318_2026_802_MOESM9_ESM.zip › Figure 5/Figure 5/5D-F/Analyzed cells/Dataset I/15253_20.tif]

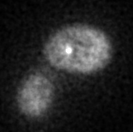

Supplement: Supplementary file 9 — Source data Fig. 5 [file 44318_2026_802_MOESM9_ESM.zip › Figure 5/Figure 5/5D-F/Analyzed cells/Dataset II/15250_01.tif]

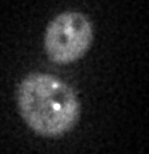

Supplement: Supplementary file 9 — Source data Fig. 5 [file 44318_2026_802_MOESM9_ESM.zip › Figure 5/Figure 5/5D-F/Analyzed cells/Dataset II/15250_02.tif]

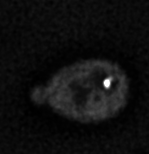

Supplement: Supplementary file 9 — Source data Fig. 5 [file 44318_2026_802_MOESM9_ESM.zip › Figure 5/Figure 5/5D-F/Analyzed cells/Dataset II/15250_03.tif]

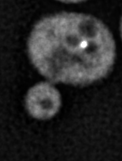

Supplement: Supplementary file 9 — Source data Fig. 5 [file 44318_2026_802_MOESM9_ESM.zip › Figure 5/Figure 5/5D-F/Analyzed cells/Dataset II/15250_04.tif]

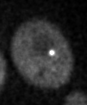

Supplement: Supplementary file 9 — Source data Fig. 5 [file 44318_2026_802_MOESM9_ESM.zip › Figure 5/Figure 5/5D-F/Analyzed cells/Dataset II/15250_05.tif]

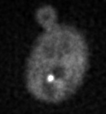

Supplement: Supplementary file 9 — Source data Fig. 5 [file 44318_2026_802_MOESM9_ESM.zip › Figure 5/Figure 5/5D-F/Analyzed cells/Dataset II/15250_06.tif]

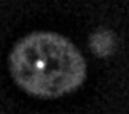

Supplement: Supplementary file 9 — Source data Fig. 5 [file 44318_2026_802_MOESM9_ESM.zip › Figure 5/Figure 5/5D-F/Analyzed cells/Dataset II/15250_07.tif]

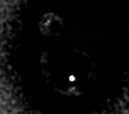

Supplement: Supplementary file 9 — Source data Fig. 5 [file 44318_2026_802_MOESM9_ESM.zip › Figure 5/Figure 5/5D-F/Analyzed cells/Dataset II/15250_08.tif]

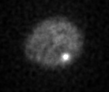

Supplement: Supplementary file 9 — Source data Fig. 5 [file 44318_2026_802_MOESM9_ESM.zip › Figure 5/Figure 5/5D-F/Analyzed cells/Dataset II/15250_09.tif]

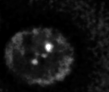

Supplement: Supplementary file 9 — Source data Fig. 5 [file 44318_2026_802_MOESM9_ESM.zip › Figure 5/Figure 5/5D-F/Analyzed cells/Dataset II/15250_10.tif]

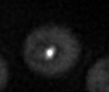

Supplement: Supplementary file 9 — Source data Fig. 5 [file 44318_2026_802_MOESM9_ESM.zip › Figure 5/Figure 5/5D-F/Analyzed cells/Dataset II/15250_11.tif]

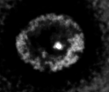

Supplement: Supplementary file 9 — Source data Fig. 5 [file 44318_2026_802_MOESM9_ESM.zip › Figure 5/Figure 5/5D-F/Analyzed cells/Dataset II/15250_12.tif]

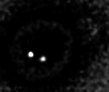

Supplement: Supplementary file 9 — Source data Fig. 5 [file 44318_2026_802_MOESM9_ESM.zip › Figure 5/Figure 5/5D-F/Analyzed cells/Dataset II/15250_13.tif]

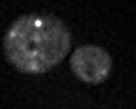

Supplement: Supplementary file 9 — Source data Fig. 5 [file 44318_2026_802_MOESM9_ESM.zip › Figure 5/Figure 5/5D-F/Analyzed cells/Dataset II/15250_14.tif]

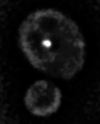

Supplement: Supplementary file 9 — Source data Fig. 5 [file 44318_2026_802_MOESM9_ESM.zip › Figure 5/Figure 5/5D-F/Analyzed cells/Dataset II/15250_15.tif]

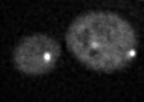

Supplement: Supplementary file 9 — Source data Fig. 5 [file 44318_2026_802_MOESM9_ESM.zip › Figure 5/Figure 5/5D-F/Analyzed cells/Dataset II/15250_16.tif]

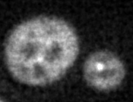

Supplement: Supplementary file 9 — Source data Fig. 5 [file 44318_2026_802_MOESM9_ESM.zip › Figure 5/Figure 5/5D-F/Analyzed cells/Dataset II/15250_17.tif]

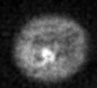

Supplement: Supplementary file 9 — Source data Fig. 5 [file 44318_2026_802_MOESM9_ESM.zip › Figure 5/Figure 5/5D-F/Analyzed cells/Dataset II/15250_18.tif]

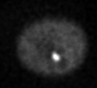

Supplement: Supplementary file 9 — Source data Fig. 5 [file 44318_2026_802_MOESM9_ESM.zip › Figure 5/Figure 5/5D-F/Analyzed cells/Dataset II/15250_19.tif]

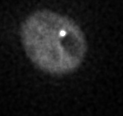

Supplement: Supplementary file 9 — Source data Fig. 5 [file 44318_2026_802_MOESM9_ESM.zip › Figure 5/Figure 5/5D-F/Analyzed cells/Dataset II/15250_20.tif]

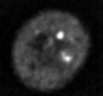

Supplement: Supplementary file 9 — Source data Fig. 5 [file 44318_2026_802_MOESM9_ESM.zip › Figure 5/Figure 5/5D-F/Analyzed cells/Dataset II/15251_01.tif]

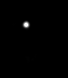

Supplement: Supplementary file 9 — Source data Fig. 5 [file 44318_2026_802_MOESM9_ESM.zip › Figure 5/Figure 5/5D-F/Analyzed cells/Dataset II/15251_02.tif]

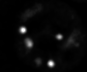

Supplement: Supplementary file 9 — Source data Fig. 5 [file 44318_2026_802_MOESM9_ESM.zip › Figure 5/Figure 5/5D-F/Analyzed cells/Dataset II/15251_03.tif]

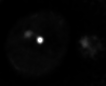

Supplement: Supplementary file 9 — Source data Fig. 5 [file 44318_2026_802_MOESM9_ESM.zip › Figure 5/Figure 5/5D-F/Analyzed cells/Dataset II/15251_04.tif]

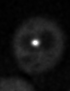

Supplement: Supplementary file 9 — Source data Fig. 5 [file 44318_2026_802_MOESM9_ESM.zip › Figure 5/Figure 5/5D-F/Analyzed cells/Dataset II/15251_05.tif]

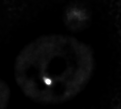

Supplement: Supplementary file 9 — Source data Fig. 5 [file 44318_2026_802_MOESM9_ESM.zip › Figure 5/Figure 5/5D-F/Analyzed cells/Dataset II/15251_06.tif]

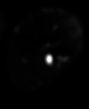

Supplement: Supplementary file 9 — Source data Fig. 5 [file 44318_2026_802_MOESM9_ESM.zip › Figure 5/Figure 5/5D-F/Analyzed cells/Dataset II/15251_07.tif]

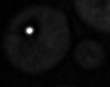

Supplement: Supplementary file 9 — Source data Fig. 5 [file 44318_2026_802_MOESM9_ESM.zip › Figure 5/Figure 5/5D-F/Analyzed cells/Dataset II/15251_08.tif]

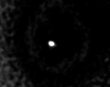

Supplement: Supplementary file 9 — Source data Fig. 5 [file 44318_2026_802_MOESM9_ESM.zip › Figure 5/Figure 5/5D-F/Analyzed cells/Dataset II/15251_09.tif]

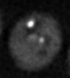

Supplement: Supplementary file 9 — Source data Fig. 5 [file 44318_2026_802_MOESM9_ESM.zip › Figure 5/Figure 5/5D-F/Analyzed cells/Dataset II/15251_10.tif]

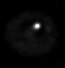

Supplement: Supplementary file 9 — Source data Fig. 5 [file 44318_2026_802_MOESM9_ESM.zip › Figure 5/Figure 5/5D-F/Analyzed cells/Dataset II/15251_11.tif]

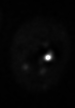

Supplement: Supplementary file 9 — Source data Fig. 5 [file 44318_2026_802_MOESM9_ESM.zip › Figure 5/Figure 5/5D-F/Analyzed cells/Dataset II/15251_12.tif]

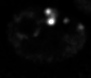

Supplement: Supplementary file 9 — Source data Fig. 5 [file 44318_2026_802_MOESM9_ESM.zip › Figure 5/Figure 5/5D-F/Analyzed cells/Dataset II/15251_13.tif]

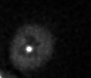

Supplement: Supplementary file 9 — Source data Fig. 5 [file 44318_2026_802_MOESM9_ESM.zip › Figure 5/Figure 5/5D-F/Analyzed cells/Dataset II/15251_14.tif]

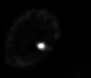

Supplement: Supplementary file 9 — Source data Fig. 5 [file 44318_2026_802_MOESM9_ESM.zip › Figure 5/Figure 5/5D-F/Analyzed cells/Dataset II/15251_15.tif]

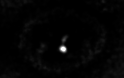

Supplement: Supplementary file 9 — Source data Fig. 5 [file 44318_2026_802_MOESM9_ESM.zip › Figure 5/Figure 5/5D-F/Analyzed cells/Dataset II/15251_16.tif]

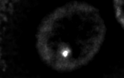

Supplement: Supplementary file 9 — Source data Fig. 5 [file 44318_2026_802_MOESM9_ESM.zip › Figure 5/Figure 5/5D-F/Analyzed cells/Dataset II/15251_17.tif]

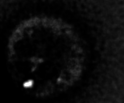

Supplement: Supplementary file 9 — Source data Fig. 5 [file 44318_2026_802_MOESM9_ESM.zip › Figure 5/Figure 5/5D-F/Analyzed cells/Dataset II/15251_18.tif]

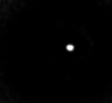

Supplement: Supplementary file 9 — Source data Fig. 5 [file 44318_2026_802_MOESM9_ESM.zip › Figure 5/Figure 5/5D-F/Analyzed cells/Dataset II/15251_19.tif]

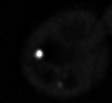

Supplement: Supplementary file 9 — Source data Fig. 5 [file 44318_2026_802_MOESM9_ESM.zip › Figure 5/Figure 5/5D-F/Analyzed cells/Dataset II/15251_20.tif]

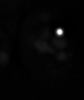

Supplement: Supplementary file 9 — Source data Fig. 5 [file 44318_2026_802_MOESM9_ESM.zip › Figure 5/Figure 5/5D-F/Analyzed cells/Dataset II/15253_01.tif]

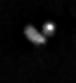

Supplement: Supplementary file 9 — Source data Fig. 5 [file 44318_2026_802_MOESM9_ESM.zip › Figure 5/Figure 5/5D-F/Analyzed cells/Dataset II/15253_02.tif]

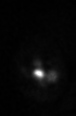

Supplement: Supplementary file 9 — Source data Fig. 5 [file 44318_2026_802_MOESM9_ESM.zip › Figure 5/Figure 5/5D-F/Analyzed cells/Dataset II/15253_03.tif]

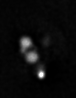

Supplement: Supplementary file 9 — Source data Fig. 5 [file 44318_2026_802_MOESM9_ESM.zip › Figure 5/Figure 5/5D-F/Analyzed cells/Dataset II/15253_04.tif]

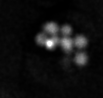

Supplement: Supplementary file 9 — Source data Fig. 5 [file 44318_2026_802_MOESM9_ESM.zip › Figure 5/Figure 5/5D-F/Analyzed cells/Dataset II/15253_05.tif]

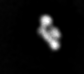

Supplement: Supplementary file 9 — Source data Fig. 5 [file 44318_2026_802_MOESM9_ESM.zip › Figure 5/Figure 5/5D-F/Analyzed cells/Dataset II/15253_06.tif]

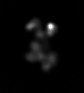

Supplement: Supplementary file 9 — Source data Fig. 5 [file 44318_2026_802_MOESM9_ESM.zip › Figure 5/Figure 5/5D-F/Analyzed cells/Dataset II/15253_07.tif]

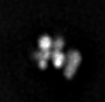

Supplement: Supplementary file 9 — Source data Fig. 5 [file 44318_2026_802_MOESM9_ESM.zip › Figure 5/Figure 5/5D-F/Analyzed cells/Dataset II/15253_08.tif]

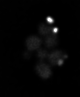

Supplement: Supplementary file 9 — Source data Fig. 5 [file 44318_2026_802_MOESM9_ESM.zip › Figure 5/Figure 5/5D-F/Analyzed cells/Dataset II/15253_09.tif]

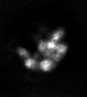

Supplement: Supplementary file 9 — Source data Fig. 5 [file 44318_2026_802_MOESM9_ESM.zip › Figure 5/Figure 5/5D-F/Analyzed cells/Dataset II/15253_10.tif]

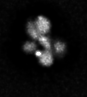

Supplement: Supplementary file 9 — Source data Fig. 5 [file 44318_2026_802_MOESM9_ESM.zip › Figure 5/Figure 5/5D-F/Analyzed cells/Dataset II/15253_11.tif]

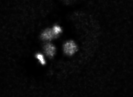

Supplement: Supplementary file 9 — Source data Fig. 5 [file 44318_2026_802_MOESM9_ESM.zip › Figure 5/Figure 5/5D-F/Analyzed cells/Dataset II/15253_12.tif]

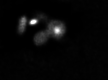

Supplement: Supplementary file 9 — Source data Fig. 5 [file 44318_2026_802_MOESM9_ESM.zip › Figure 5/Figure 5/5D-F/Analyzed cells/Dataset II/15253_13.tif]

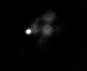

Supplement: Supplementary file 9 — Source data Fig. 5 [file 44318_2026_802_MOESM9_ESM.zip › Figure 5/Figure 5/5D-F/Analyzed cells/Dataset II/15253_14.tif]

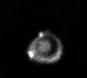

Supplement: Supplementary file 9 — Source data Fig. 5 [file 44318_2026_802_MOESM9_ESM.zip › Figure 5/Figure 5/5D-F/Analyzed cells/Dataset II/15253_15.tif]

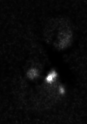

Supplement: Supplementary file 9 — Source data Fig. 5 [file 44318_2026_802_MOESM9_ESM.zip › Figure 5/Figure 5/5D-F/Analyzed cells/Dataset II/15253_16.tif]

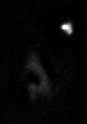

Supplement: Supplementary file 9 — Source data Fig. 5 [file 44318_2026_802_MOESM9_ESM.zip › Figure 5/Figure 5/5D-F/Analyzed cells/Dataset II/15253_17.tif]

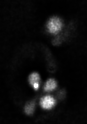

Supplement: Supplementary file 9 — Source data Fig. 5 [file 44318_2026_802_MOESM9_ESM.zip › Figure 5/Figure 5/5D-F/Analyzed cells/Dataset II/15253_18.tif]

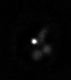

Supplement: Supplementary file 9 — Source data Fig. 5 [file 44318_2026_802_MOESM9_ESM.zip › Figure 5/Figure 5/5D-F/Analyzed cells/Dataset II/15253_19.tif]

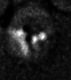

Supplement: Supplementary file 9 — Source data Fig. 5 [file 44318_2026_802_MOESM9_ESM.zip › Figure 5/Figure 5/5D-F/Analyzed cells/Dataset II/15253_20.tif]

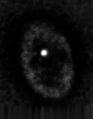

Supplement: Supplementary file 9 — Source data Fig. 5 [file 44318_2026_802_MOESM9_ESM.zip › Figure 5/Figure 5/5D-F/Analyzed cells/Dataset III/15250_01.tif]
