# Supplementary material for: Atg18 interaction positions Atg2 for efficient lipid transfer into phagophore elongation
Source: EMBO J. 2026 May 20;45(12):4034–60. doi: 10.1038/s44318-026-00802-3 (PMC13269710; doi:10.1038/s44318-026-00802-3)
Supplement: Supplementary file 12 — Expanded View Figures [file 44318_2026_802_MOESM12_ESM.pdf]

Expanded View Figures

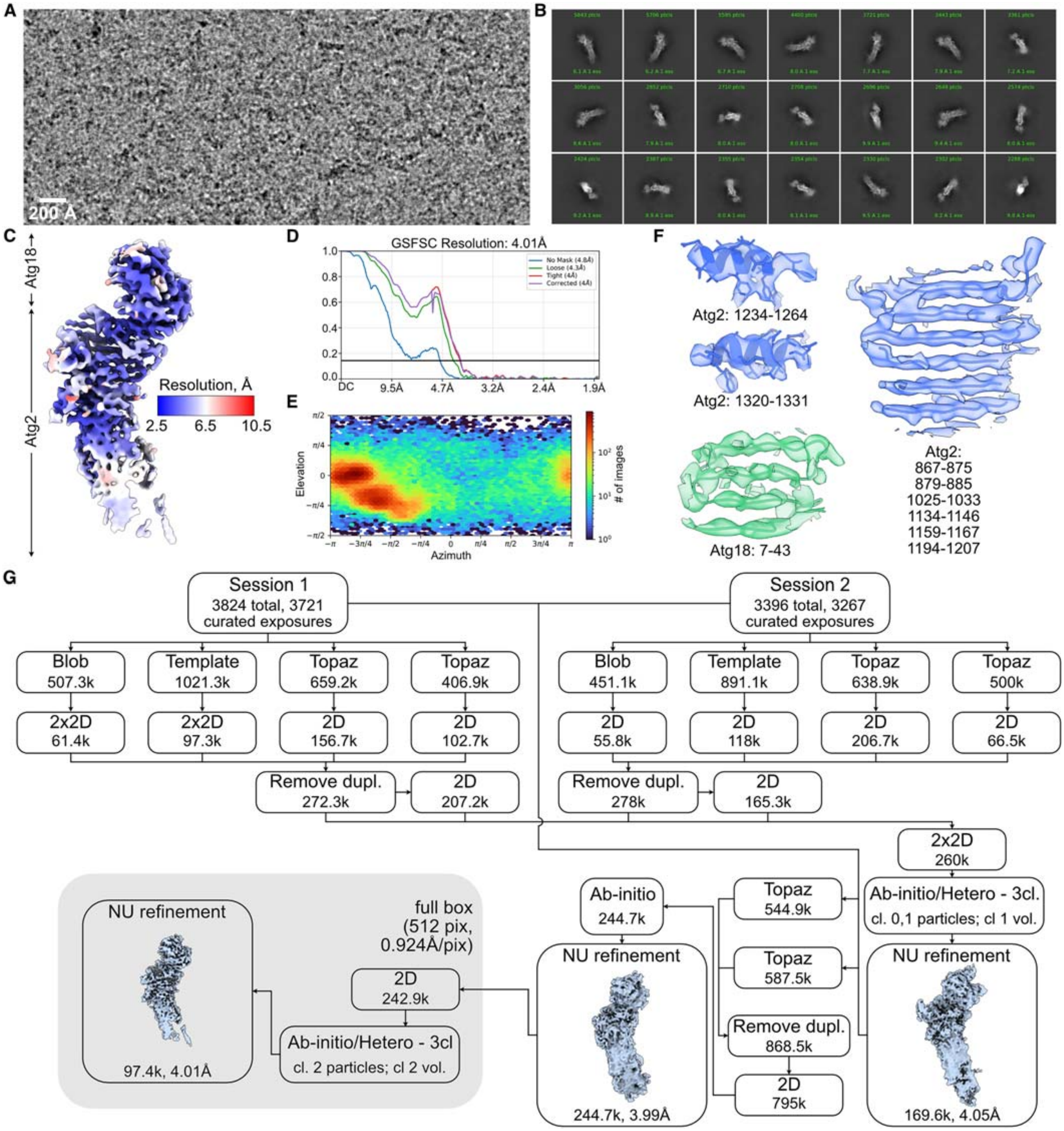

Figure EV1. Atg2-18 complex cryo-EM single particle data analysis and validation.

(**A**) Representative cryo-EM micrograph of the Atg2-Atg18 sample. (**B**) Representative 2D class averages, box size is 473 Å. (**C**) Local resolution estimation map generated using the cryoSPARC software, colored by local resolution. (**D**) cryoSPARC-generated Gold standard Fourier shell correlation (GSFSC) curve. (**E**) cryoSPARC-generated angular distribution heatmap plot. (**F**) Model/map fitting of selected segments (residues are indicated) of Atg2 (blue) and Atg18 (green). (**G**) Cryo-EM data processing pipeline. Numbers of particles used and resolutions achieved in the refinements are indicated. Cl. class, vol. volume, pix pixel.

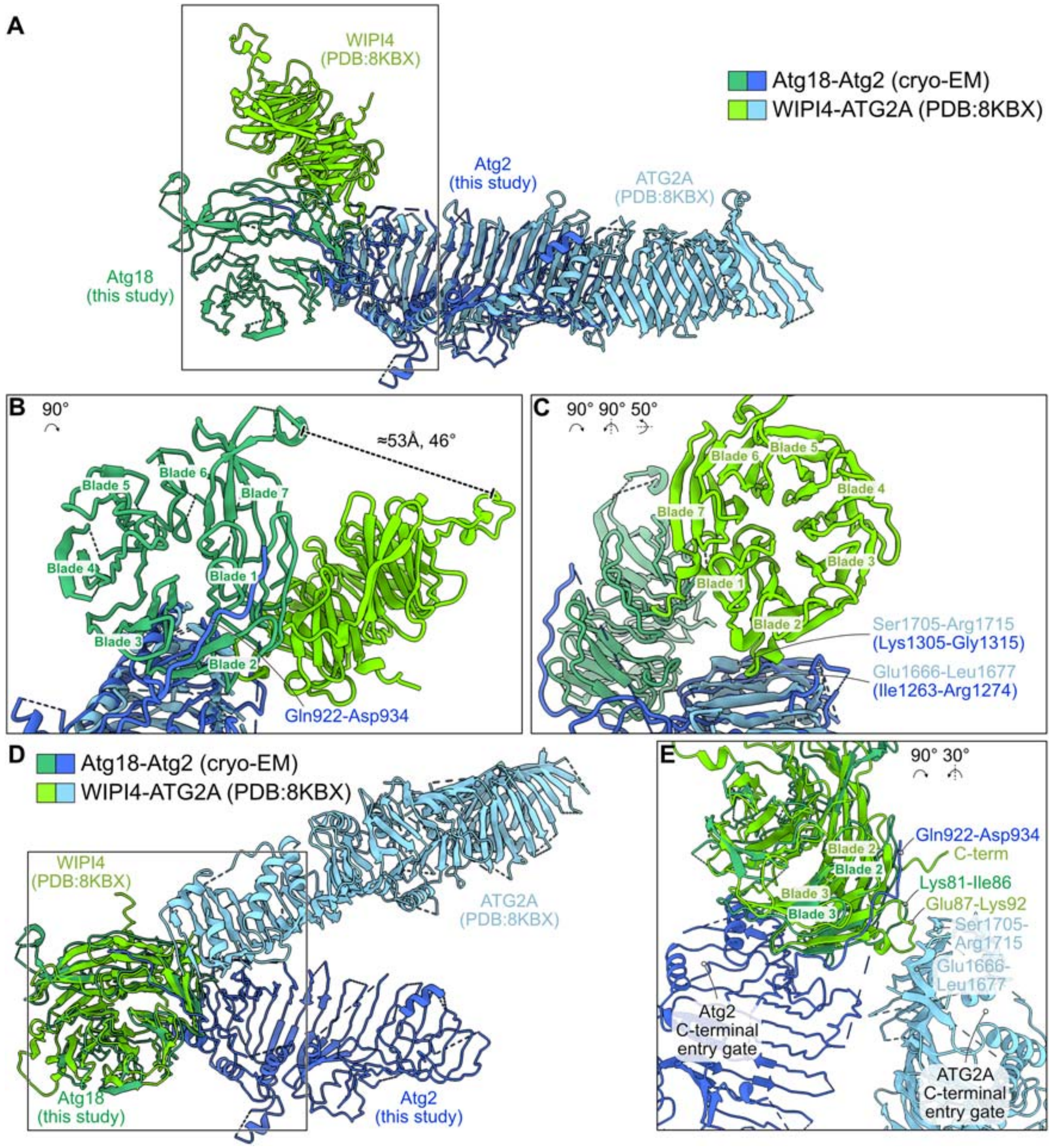

Figure EV2. Comparison of the yeast Atg18-Atg2 structure (this study) with the human ATG2-WIPI4 structure (PDB:8KBX).

(**A**) Superposition of the yeast (Atg2, dark blue, Atg18, dark green; this study) and human structure (ATG2A, light blue, WIPI4, light green; PDB:8KBX), aligned based on Atg2 using the *matchmaker* command in ChimeraX. (**B**) Close-up view of Atg18/WIPI4 from the superposition shown in (**A**). Blades of the Atg18 β-propeller are indicated. The distance between the most distal points of the β-propellers of Atg18 and WIPI4 relative to Atg2 and ATG2A, respectively, as well as the rotation angle of WIPI4 relative to Atg18, are indicated. The 922–934 region of Atg2 that is involved in the interaction with Atg18 is indicated. (**C**) Close-up view of the WIPI4 face of the superposition shown in (**A**). Blades of the WIPI4 β-propeller are indicated. The ATG2A regions that are involved in the interface with WIPI4, along with the corresponding regions of Atg2, are also highlighted. (**D**) Superposition of the yeast (Atg2, dark blue, Atg18, dark green; this study) and human structure (ATG2A, light blue, WIPI4, light green; PDB:8KBX) aligned based on Atg18 using the *matchmaker* command in ChimeraX. (**E**) Close-up view of Atg18/WIPI4 from the superposition shown in (**D**). Blades of the Atg18 and WIPI4 β-propellers are indicated. The 922–934 region of Atg2 (as in (**B**)), as well as the ATG2A regions that are involved in the interaction interface with WIPI4 are indicated (as in (**C**)).

 © The Author(s)

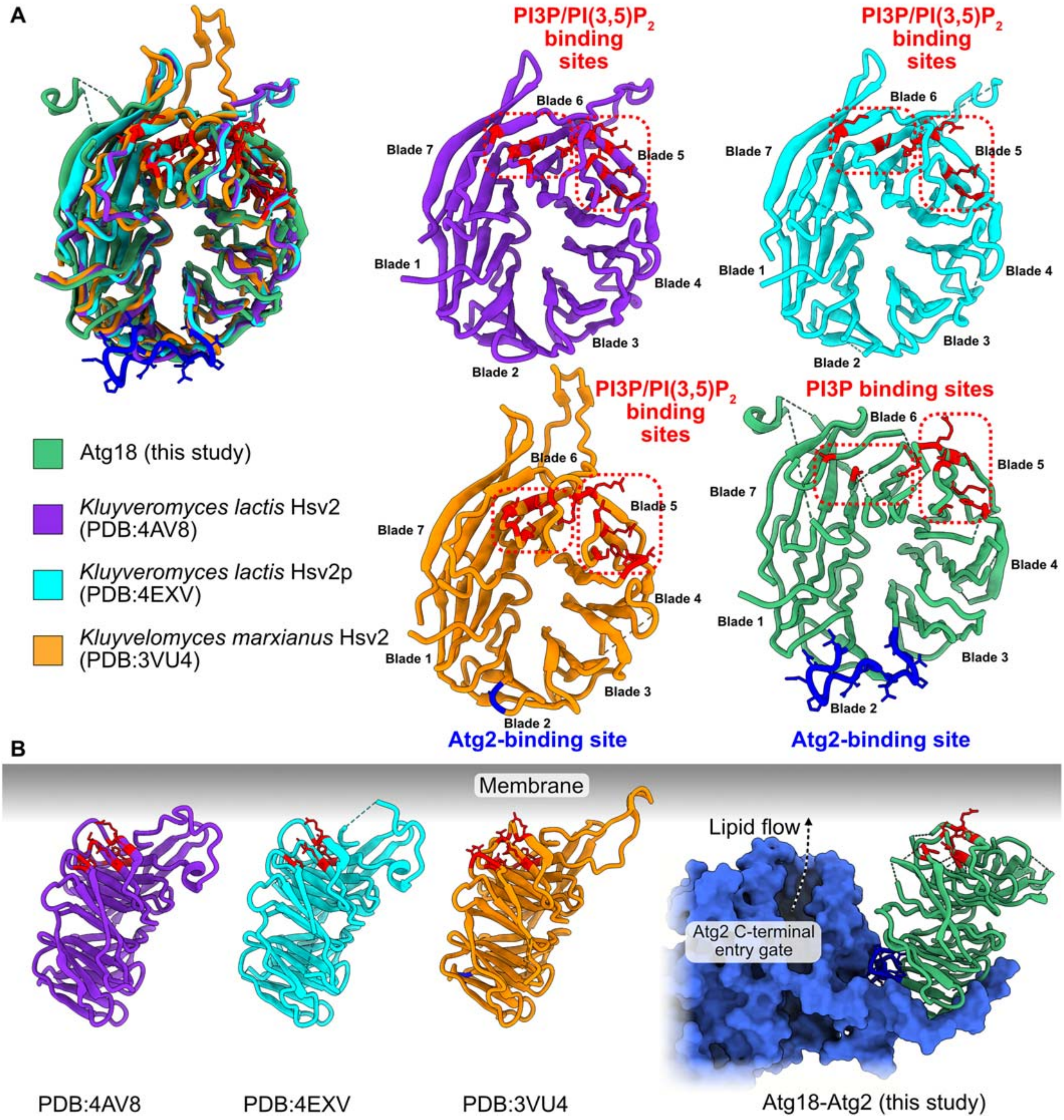

Figure EV3. Comparison of the Atg18 and Hsv2 PROPPINs, highlighting the phosphoinositide binding sites.

(**A**) Structural comparison of Atg18 (this study) and Hsv2 structures (PDB:4AV8, 4EXV, 3VU4): superposition (left top panel) and individual structures. Residues responsible for phosphoinositide binding are colored red and the phosphoinositide-binding pockets are highlighted by red dotted rounded rectangles. Amino acid residues involved in the interactions with Atg2 are colored blue. (**B**) Schematic model of Hsv2 and Atg18 interaction with membranes (gray). Left, Hsv2 proteins from (**A**) rotated by 90° with the phosphoinositide-binding sites (red) facing the membrane. Right, the Atg2-Atg18 structure (cryo-EM) with Atg18 positioned analogously to Hsv2 structures on the left. A hypothetical orientation of the Atg2-Atg18 complex with Atg18's phosphoinositide-binding sites interacting with the membrane would permit lipid flow through the C-terminal gate of Atg2.

© The Author(s)

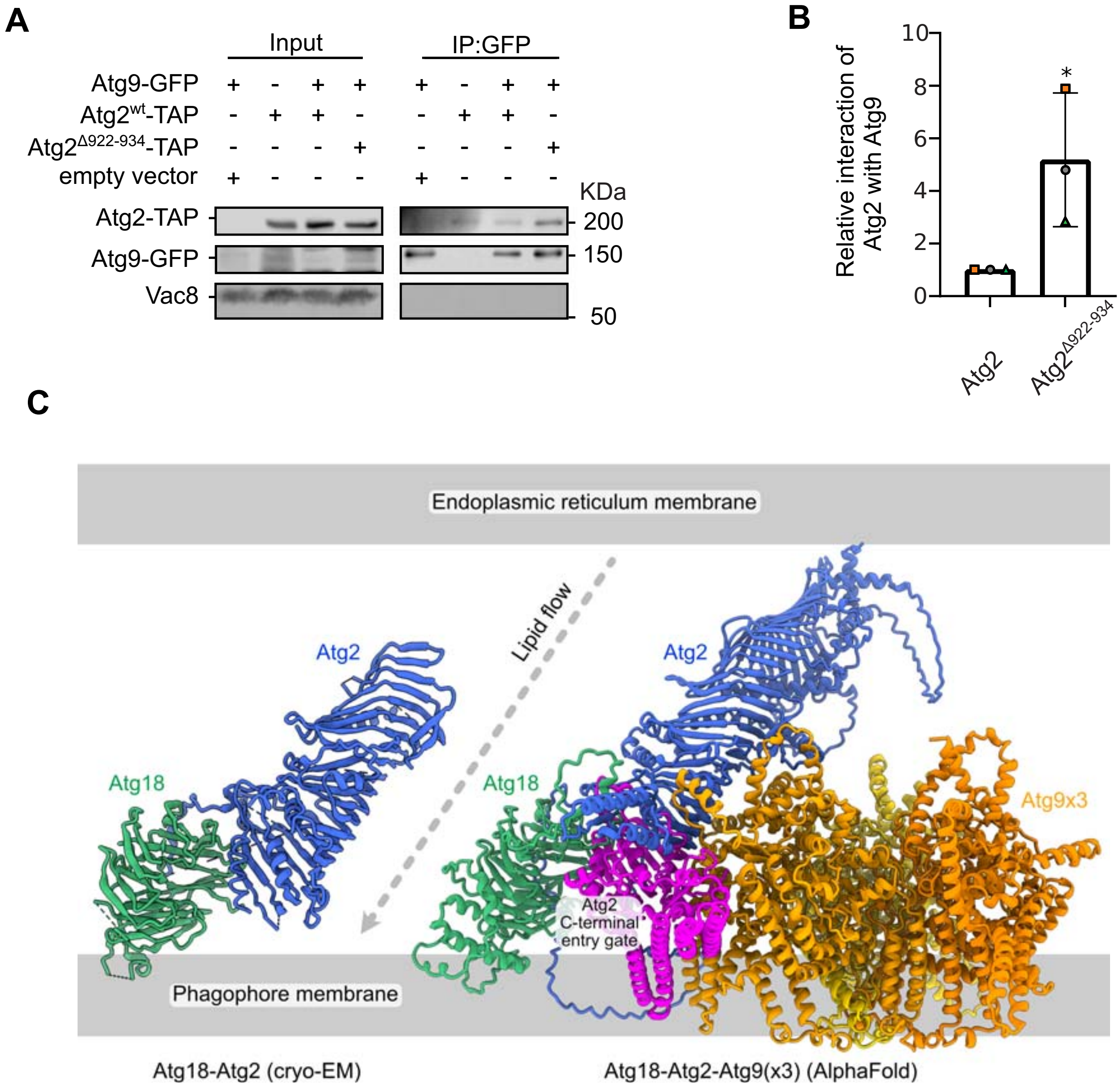

Figure EV4. Possible interaction of Atg2-Atg18 with Atg9.

(**A**) The *atg2Δ* and *atg2Δ* Atg18-13xmyc strains expressing endogenous Atg9-GFP and transformed with integrative plasmids carrying Atg2-TAP or Atg2$^{Δ922-934}$-TAP, or an empty vector were grown in YPD to a log phase and starved for 1 h in SD-N medium to induce autophagy. Atg9-GFP and associated proteins were co-immunoprecipitated from total cell lysates using GFP-Trap beads (IP:GFP). Proteins eluted from the beads were analyzed by western blot using anti-GFP, anti-TAP or anti-Vac8 antibodies. Vac8 served as the loading control for the total lysates. (**B**) Quantification of (**A**). The graph shows the ratio of co-immunoprecipitation of Atg2$^{Δ922-934}$-TAP with Atg9-GFP relative to that of Atg2-TAP. The graph shows the mean value and the standard deviation of three independent experiments. Statistical analysis was conducted by two-tailed unpaired *t* test with 95% confidence intervals. *P* value is as follows: Atg2$^{Δ922-934}$ (PVY129) vs Atg2 (RGY590), $P = 0.0465$. (**C**) Hypothetical positioning of Atg2-Atg18 complex at the membrane (left, cryo-EM structure) and the AlphaFold3 model of the Atg2-Atg18 complex in association with the Atg9 trimer, positioned similarly in the membrane. The fragment of Atg2 corresponding to the ATG9A-interacting ATG2A fragment 4 (van Vliet et al, 2022) is colored in magenta. The orientation of the proteins in both the cryo-EM structure and the AlphaFold3 model allows the simultaneous interaction of Atg2 with Atg9 and Atg18, membrane binding by Atg18, and positioning of the Atg2 C-terminal channel gate toward the membrane for lipid transfer (gray dotted arrow).

 © The Author(s)

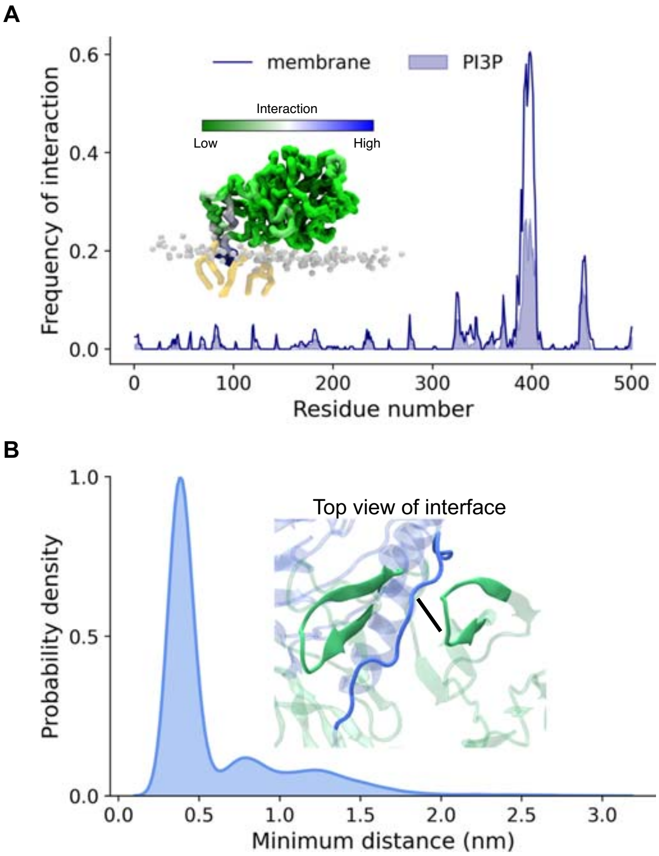

Figure EV5. CG-MD analysis of Atg18 interaction with the membrane and its association with the Atg2 loop.

(**A**) Atg18 spontaneously binds to a membrane primarily through interactions with PtdIns3P. Dark traces show the binding to membranes, while the lighter traces show interactions with PtdIns3P only. The inset shows the protein colored by its membrane interaction frequency. (**B**) Probability density of the interactions between Atg18 and Atg2 (residues 922–934) at their binding interfaces.
