# Supplementary material for: Atg18 interaction positions Atg2 for efficient lipid transfer into phagophore elongation
Source: EMBO J. 2026 May 20;45(12):4034–60. doi: 10.1038/s44318-026-00802-3 (PMC13269710; doi:10.1038/s44318-026-00802-3)
Supplement: Supplementary file 4 — Peer Review File [file 44318_2026_802_MOESM4_ESM.pdf]

THE EMBO JOURNAL

Atg18 interaction positions Atg2 for efficient lipid transfer into phagophore elongation

Sabrina Chumpen Ramirez, Dmitry Shvarev, Prado Vargas Duarte, Yara Ahmed, Jana Milach, Emma Lang, Stefan Kuchenbuch, Stefano Vanni, Fulvio Reggiori, Arne Moeller, and Christian Ungermann

Corresponding author(s): Christian Ungermann (cu@uos.de) , Fulvio Reggiori (f.m.reggiori@aias.au.dk), Stefano Vanni (stefano.vanni@unifr.ch), Arne Moeller (arne.moeller@uni-osnabrueck.de)

Review Timeline:

Submission Date:	17th Oct 25
Editorial Decision:	4th Dec 25
Revision Received:	6th Mar 26
Editorial Decision:	1st Apr 26
Revision Received:	15th Apr 26
Accepted:	20th Apr 26

Editor: William Teale

Transaction Report:

(Note: With the exception of the correction of typographical or spelling errors that could be a source of ambiguity, letters and reports are not edited. Depending on transfer agreements, referee reports obtained elsewhere may or may not be included in this compilation. Referee reports are anonymous unless the Referee chooses to sign their reports.)

Dear Christian,

Thank you again for the submission of your manuscript entitled "Atg18 gates Atg2 for efficient lipid transfer into phagophore elongation" and for your patience during the review process. We have now received reports from three referees, which I copy below.

As you can see from their comments, all thought that your manuscript could represent a useful and timely development to our understanding of Atg2-mediated lipid transfer. That said, all of them point out potential contradictions that may need additional experimentation to resolve. This will require your attention before your manuscript can be published in The EMBO Journal.

Based on the overall interest expressed in the reports, however, I would like to invite you to address the comments of all referees in a revised version of the manuscript. I should add that it is The EMBO Journal policy to allow only a single major round of revision and that it is therefore important to resolve the main concerns at this stage. I believe the concerns of the referees are reasonable and addressable, but please contact me if you have any questions, need further input on the referee comments or if you anticipate any problems in addressing any of their points. Please, follow the instructions below when preparing your manuscript for resubmission.

I would also like to point out that as a matter of policy, competing manuscripts published during this period will not be taken into consideration in our assessment of the novelty presented by your study ("scooping" protection). We have extended this 'scooping protection policy' beyond the usual 3 month revision timeline to cover the period required for a full revision to address the essential experimental issues. Please contact me if you see a paper with related content published elsewhere to discuss the appropriate course of action.

When preparing your letter of response to the referees' comments, please bear in mind that this will form part of the Review Process File, and will therefore be available online to the community. For more details on our Transparent Editorial Process, please visit our website: https://link.springer.com/journal/44318/submission-guidelines#cms-Revised-submissions

Again, please contact me at any time during revision if you need any help or have further questions.

Thank you very much again for the opportunity to consider your work for publication. I look forward to your revision.

Best regards,

William

William Teale, Ph.D.
Editor
The EMBO Journal

When submitting your revised manuscript, please carefully review the instructions below and include the following items:

1) a .docx formatted version of the manuscript text (including legends for main figures, EV figures and tables). Please make sure that the changes are highlighted to be clearly visible.

2) individual production quality figure files as .eps, .tif, .jpg (one file per figure).

3) a .docx formatted letter INCLUDING the reviewers' reports and your detailed point-by-point response to their comments. As part of the EMBO Press transparent editorial process, the point-by-point response is part of the Review Process File (RPF), which will be published alongside your paper.

4) a complete author checklist, which you can download from our author guidelines (https://link.springer.com/journal/44318/submission-guidelines#cms-Revised-submissions). Please insert information in the checklist that is also reflected in the manuscript. The completed author checklist will also be part of the RPF.

5) Please note that all corresponding authors are required to supply an ORCID ID for their name upon submission of a revised manuscript.

6) We require a 'Data Availability' section after the Materials and Methods. Before submitting your revision, primary datasets produced in this study need to be deposited in an appropriate public database, and the accession numbers and database listed under 'Data Availability'. Please remember to provide a reviewer password if the datasets are not yet public (see

https://link.springer.com/partners/embo-press/editorial-policies#Data%20deposition). If no data deposition in external databases is needed for this paper, please then state in this section: This study includes no data deposited in external repositories. Note that the Data Availability Section is restricted to new primary data that are part of this study.

Note - All links should resolve to a page where the data can be accessed.

7) When assembling figures, please refer to our figure preparation guideline in order to ensure proper formatting and readability in print as well as on screen:
https://link.springer.com/journal/44318/submission-guidelines#cms-Figure-and-data-presentation

Please remember: Digital image enhancement is acceptable practice, as long as it accurately represents the original data and conforms to community standards. If a figure has been subjected to significant electronic manipulation, this must be noted in the figure legend or in the 'Materials and Methods' section. The editors reserve the right to request original versions of figures and the original images that were used to assemble the figure.

8) For data quantification: please specify the name of the statistical test used to generate error bars and P values, the number (n) of independent experiments (specify technical or biological replicates) underlying each data point and the test used to calculate p-values in each figure legend. The figure legends should contain a basic description of n, P and the test applied. Graphs must include a description of the bars and the error bars (s.d., s.e.m.).

9) We would also encourage you to include the source data for figure panels that show essential data. Numerical data can be provided as individual .xls or .csv files (including a tab describing the data). For 'blots' or microscopy, uncropped images should be submitted (using a zip archive or a single pdf per main figure if multiple images need to be supplied for one panel). Additional information on source data and instruction on how to label the files are available at
https://link.springer.com/journal/44318/submission-guidelines#cms-Source-data

10) We replaced Supplementary Information with Expanded View (EV) Figures and Tables that are collapsible/expandable online (see examples in https://www.embopress.org/doi/10.15252/embj.201695874). A maximum of 5 EV Figures can be typeset. EV Figures should be cited as 'Figure EV1, Figure EV2" etc. in the text and their respective legends should be included in the main text after the legends of regular figures.

- For the figures that you do NOT wish to display as Expanded View figures, they should be bundled together with their legends in a single PDF file called *Appendix*, which should start with a short Table of Content. Appendix figures should be referred to in the main text as: "Appendix Figure S1, Appendix Figure S2" etc. See detailed instructions regarding expanded view here: < https://link.springer.com/journal/44318/submission-guidelines#cms-Expanded-View-data >.

- Additional Tables/Datasets should be labelled and referred to as Table EV1, Dataset EV1, etc. Legends have to be provided in a separate tab in case of .xls files. Alternatively, the legend can be supplied as a separate text file (README) and zipped together with the Table/Dataset file.

11) At EMBO Press we ask authors to provide source data for the main manuscript figures. Our source data coordinator will contact you to discuss which figure panels we would need source data for and will also provide you with helpful tips on how to upload and organize the files.

12) Our journal encourages inclusion of *data citations in the reference list* to directly cite datasets that were re-used and obtained from public databases. Data citations in the article text are distinct from normal bibliographical citations and should directly link to the database records from which the data can be accessed. In the main text, data citations are formatted as follows: "Data ref: Smith et al, 2001" or "Data ref: NCBI Sequence Read Archive PRJNA342805, 2017". In the Reference list, data citations must be labeled with "[DATASET]". A data reference must provide the database name, accession number/identifiers and a resolvable link to the landing page from which the data can be accessed at the end of the reference. Further instructions are available at < https://link.springer.com/journal/44318/submission-guidelines#cms-Reference-guidelines >.

13) In order to increase the reproducibility and reach of your work, The EMBO Journal includes a table of reagents that were used in the study. Please provide this along with your revisions.

Further instructions for preparing your revised manuscript:

Read our guidance for manuscript revisions and related editorial policies: https://link.springer.com/journal/44318/submission-guidelines#cms-Revised-submissions

Please make sure you upload a letter of response to the referees' comments together with the revised manuscript.

Please also check that the title and abstract of the manuscript are brief, yet explicit, even to non-specialists.

When assembling figures, please refer to our figure preparation guideline in order to ensure proper formatting and readability in print as well as on screen:
https://media.springernature.com/original/springer-cms/rest/v1/content/27825798/data/v1

At EMBO Press we ask authors to provide source data for the main manuscript figures. You will receive a separate email with instructions for providing source data with your revised manuscript, including how to upload and organize the files.

IMPORTANT: When you send the revision we will require
- a point-by-point response to the referees' comments, with a detailed description of the changes made (as a word file).
- a word file of the manuscript text.
- individual production quality figure files (one file per figure)
- a complete author checklist
- Expanded View files (replacing Supplementary Information)
- a Reagents and Tools Table as part of the Methods section

Please remember: Digital image enhancement is acceptable practice, as long as it accurately represents the original data and conforms to community standards. If a figure has been subjected to significant electronic manipulation, this must be noted in the figure legend or in the 'Materials and Methods' section. The editors reserve the right to request original versions of figures and the original images that were used to assemble the figure.

We realize that it is difficult to revise to a specific deadline. In the interest of protecting the conceptual advance provided by the work, we recommend a revision within 3 months (4th Mar 2026). Please discuss the revision progress ahead of this time with the editor if you require more time to complete the revisions. Use the link below to submit your revision:

https://emboj.msubmit.net/cgi-bin/main.plex

Referee #1:

The study investigates the molecular mechanism of lipid transfer during autophagosome formation, focusing on the interaction between Atg2 and Atg18 in yeast. The cryo-EM structure of the complex between Atg2 and Atg18 is reported. Consistent with previous findings, Atg2 was found to adapt a tube-like shape with a hydrophobic cavity filled with lipids densities that correspond to lipids. Atg18 interacts with Atg2 via a critical interface involving Atg2 residues 922-934 and Atg18 blades 2 and 3. This interaction strongly increases lipid transport by Atg2 in the presence of PI3P, but not in the absence of PI3P. This suggests that Atg18 requires PI3P to support Atg2 function, which is consistent with the requirement of PI3P for Atg18 binding and its proper orientation on membranes.
The lipid transfer assay also shows that Atg9 increases lipid transport of Atg2 alone, or in presence of Atg18, in a PI3P independent manner. Consistent with the observed interaction interface between Atg2 and Atg18, deletion of Atg2 residues 922-934 disrupts its interaction with Atg18, impairing Atg18 recruitment to the PAS and abolishing Atg18-mediated stimulation of lipid transfer. This mutation is also deficient in promoting phagophore elongation and autophagy progression. Based on the experimental data, a model is proposed in which Atg2 receives lipids from the ER and interacts with Atg9 trimers at the phagophore. Atg18 binds to Atg2 in a PtdIns3P-dependent manner, gating the lipid channel and enabling efficient lipid transfer for phagophore expansion.
Overall, this study is of great significance for the autophagy field and beyond. The described function of Atg18 as a gatekeeper of Atg2 is exciting and new. There are, however, some fundamental concerns that need to be addressed before a recommendation on the publication of the manuscript in the EMBO journal can be made.
1. A major concern is the orientation of Atg18 in the complex structure. Compared to the structure of the human ATG2/WIPI4 complex, Atg18 is twisted by 46{degree sign}, which would detach Atg18 from the membrane, since its PI3P and membrane binding sites are oriented such that an interaction with membranes becomes impossible. The reason for this movement and the biologic significance of this orientation remains unclear. This becomes even more relevant as the binding site between Atg18 and Atg2, involving Atg2 residues 922-934, is located in a loop region of Atg2, suggesting that this interaction could be maintained even if Atg18 had another orientation relative to Atg2. The question of the biological relevance of the observed structure needs to be evaluated to strengthen the conclusions of the manuscript. A possibility would be to show that the orientation of Atg18 on membranes is maintained as seen in the structure of the soluble complex.
2. Given the limitation of the interpretation of the Atg18/Atg2 complex structure, the correlation between functional assay (Figure 1) and the structure is difficult. In particular, the stimulation of the lipid transfer capacity of Atg2 by Atg18 was shown to strictly depend on the presence of PI3P, which is inconsistent with the observed orientation of Atg18 in the complex. This contradiction needs to be resolved by providing experimental evidence. For example, it could be demonstrated that Atg18 undergoes a conformational change if it engages Atg2 after it has been recruited to the membrane in an PI3P dependent manner.
3. Furthermore, the orientation of Atg18 appears to induce a steric clash of the membrane binding region of Atg2, in particular

the entrance of the lipid transport channel, with Atg9. The model in Figure 7 is not very informative, but MD simulations could be carried out to show that the complex indeed interacts with membranes in a way shown in the cartoon and that the tunnel opening is in direct contact with Atg9 and in close proximity to membranes.
4. Another major limitation of the study is the missing structure of N-terminal part of Atg2. The argument that structural flexibility and biased orientation is a reason that this part of Atg2 is not resolved is not entirely conclusive. The beta-sheet structure is usually quite stable suggesting that the overall structure of Atg2 should be quite stable as well. Moreover, a biased orientation of Atg2 should not only affect the C-terminal part, but the overall structure, since Atg2 is a long rod-shaped protein. Can the authors provide a more convincing argumentation? They could, for example, determine the structural flexibility of Atg2 by NMR or hydrogen exchange experiments.

Referee #2:

Ramirez report an atomically interpretable structure of the yeast Atg2-Atg18 complex and in vitro and in vivo functional assays of the main Atg18-binding loop of Atg2. This is a very straightforward exposition of a structure corroborated by the obvious assays, and the conclusion is clear and internally consistent. In some ways this is a stronger study than the related papers by on the ATG2A:WIPI3/4 interaction by Wang et al. NSMB (2025) and Ren et al. N. Comms. (2020), which did not report functional assessment of mutations in ATG2A and thus left a gap. In principle the contribution merits publication in EMBO J but substantial improvements are needed.

The structure suffers from substantial preferred orientations and an unhealthy-looking FSC. The map quality is at the borderline where side-chain assignments can be trusted. Given an Alpha Fold starting model, it is probably OK. The clash score is passable although not great.

I note that the Wang NSMB paper on ATG2A suffered even more, and symptoms were apparent even in the Kang 2025 Nature paper on LPD-3. I don't know how much more it can be improved, but it would be worth at least noting this issue in the results section.

Fig. 2F should show the side chains in the 922-934 sequence, not just the main chain trace.

One major issue is that the nature and identity of the functionally relevant WIPI binding sites in human vs yeast ATG2A.

The authors should read and cite the paper by J. Ren et al in N. Comms. (2020). The binding site on the WIPI side in the present work and Ren. On the other hand, Ren et al. implicate the Sc Atg2 sequence 1193-PWPHSSPMF-1201 as the Atg18 binding site, which does not match even the secondary (non-functional) 1149-1157 sequence. Is there some chance the 1149-1157 sequence in this paper was mis-assigned, or did Ren get it completely wrong? This should be clarified with additional figures showing structural superpositions of the current structure and Ren's ATG2A-WIPI3 complex, and detailed density for the 1149-1157 sequence showing side chains as docked in density and (if the density alone is insufficient to justify the assignment), also the Alpha Fold metrics.

The superposition with the Wang structure is shown as aligned based on the ATG2 portion, but the WIPI4/Atg18 comparison should also be carried out. The structural alignment of the Atg2 and ATG2A should be shown and the sequences written out and aligned on the basis of the WIPI-driven structural alignment, and the results discussed relative to Ren et al and relative to the functional data.

The use of the word "gates" in the title implies to me that Atg18 regulates the opening of the Atg2 lipid channel relative to the unbound Atg2 protein. The authors don't show this, however. The word "targets" would be more honest.

Overall, the discussion and Fig. 7 are overly focused on restating various speculative opinions in the field relating to gating and the role of Atg9. I recommend removing Fig. 7 which shows a tight complex between Atg2 and Atg9 that is backed up by no data that I am aware of, and certainly not supported by the data in this manuscript. Refocus the discussion on the comparative structural analysis of the various Atg2/ATG2A plus Atg18/WIPI3/4 structures now available.

Watanabe is correctly cited for pointing out the Atg18 residues important for Atg2 interaction, however, the authors should still cite the original Hsv2 structure papers that predated Watanabe, from Baskaran et al 2012 and Krick et al 2012.

Referee #3:

Summary

The manuscript by presents a structural and functional analysis of the yeast Atg2-Atg18 complex, a key lipid transfer module at the ER-phagophore membrane contact site during macroautophagy. Using cryo-electron microscopy, the authors determine the structure of Atg2 in complex with Atg18 and characterize the Atg18-binding interface in Atg2 that is required for Atg18 recruitment and lipid transfer activity. They visualise continuous lipid densities along the internal hydrophobic cavity of Atg2, providing direct structural evidence that the Atg2 channel is filled with lipids. Complementary in vitro and in vivo experiments support a model in which Atg18 positions and gates the Atg2 lipid channel to promote directional lipid flux and efficient phagophore elongation during both bulk and selective autophagy. Overall, the manuscript is very nice with strong structural and functional data; the in vitro and in vivo validation makes a compelling case for the importance of the newly identified Atg2-Atg18 interface in lipid transfer-mediated phagophore expansion. The manuscript is well written, with a concise and clear text.

Major points

Why have the authors not introduced point mutations (e.g. alanine substitutions) into the Atg18-Atg2 interface instead of deleting such a long stretch of Atg2? Removing residues 922-934 may have broader functional consequences and could affect Atg9 binding. The authors should test whether the Atg2Δ922-934 mutant can still bind Atg9 and, if not, design alternative Atg18-Atg2 interface mutants that more selectively disrupt Atg18 binding.

For key experiments, the authors use a HaloTag on endogenous Atg18, a GFP tag on Atg2 and mCherry on Atg8. They should demonstrate that autophagy is not significantly affected by these tags. Pho8Δ60 assays should be included for the relevant tagged constructs to directly address this point.

Minor points

For the cryo-EM structure, the authors should map the local resolution onto the density map (and include this as a supplementary figure) and briefly describe in the main text how the atomic model was built into the map.

Can Atg9 and Atg18 bind Atg2 simultaneously, or are these interactions mutually exclusive? Could the authors use their system together with recombinant Atg9 to address these questions experimentally?

In Fig. 4A and 5C, the authors should clarify how mCherry-Atg8 and Atg2-GFP were expressed. Were the endogenous proteins tagged at the genomic locus, or were tagged versions overexpressed in the presence of wild-type proteins?

The authors should explain why they chose GFP-tagged constructs, which can dimerise, rather than using monomeric EGFP-tagged constructs.

The constructs used for the Pho8Δ60 experiments in Fig. 3C need to be more clearly specified in both the figure and the legend. If the comparison is between untagged wild-type Atg2 and a GFP-tagged Atg2 mutant, the authors should also include wild-type Atg2-GFP. Conversely, if all constructs are GFP-tagged, untagged Atg2 (ideally both wild type and mutant) should be included to disentangle the effect of the GFP tag from the mutation.

In Fig. 4A it would be helpful to include an example of a giant Ape1 structure that is similar in size to the example shown for wild-type Atg2. In addition, the authors should quantify the diameter of the giant Ape1 structures for all 27 cells and compare these measurements between the Atg2 wild-type and mutant datasets, to rule out the possibility that systematically smaller Ape1 structures in the mutant condition bias the analysis.

In Fig. 5D, the authors use an unpaired two-tailed t-test. Given that the data do not appear to be normally distributed, is a t-test the most appropriate statistical test here? The authors should justify the use of the t-test or consider using an alternative.

We thank the reviewers for the critical analysis of our work and their input. Our responses are below each point and written in blue. According to their recommendations and the Journal specifications we included the following changes in our manuscript and figures:

1- We changed the title of the manuscript to "Atg18 interaction positions Atg2 for efficient lipid transfer into phagophore elongation".
2- Supplementary figures are now labeled as Expansion view (EV) or Appendix Figure S.
3- We updated Figure 2E, now showing the PtdIns3P-binding sites in the structure.
4- We corrected the labelling of plots in Figure 3B and 3C and replaced the plot in panel 3C by the results using the no tagged versions of the Atg2 variants.
5- We updated Figure 4 as suggested by reviewer #3.
6- We updated Figure 5 as requested by reviewer #3 and corrected the labelling of the Figure 5A (before Atg2-TAP, now Atg2-GFP).
7- We included a new Figure 6, and a new Appendix Figure S6, showing the FM analysis of Ypt1 and Trs85 recruitment to the PAS, and the PAS association with the ER and ERES. We included the results in lines 266-272.
8- We performed molecular dynamics (MD) simulations to reinforce our conclusions, and included a new main figure, i.e., Figure 8.
9- We removed our working model and now show the MD data in Figure 8.
10- We included three new supplementary figures: Figure EV3, EV4 and EV5.
11- The previous supplementary Figure 2 (FigureS2_Atg2-Atg18_AF) is now Appendix Figure S2
12- We included four new supplementary figures: Appendix Figure S1, S3, S4 and S5.
13- We modified the abstract on lines 40-12 and the introduction on lines 89-92.
14- We modified the result section on lines 132-148, 155-168, 181-183, 188-199, 209-217, 225-228 and 239-260.
15- We included a new section in the *Results* describing the MD simulations.
16- We modified the discussion section on lines: 329-332, 335, 337, 339, 350, 384-389 and 397-402.
17- We have updated the *Materials and Method* section.
18- We updated the legend of Figures 3C (lines 913-916), 4A (lines 923-927), 4B (line 933), 4C (lines 936-937), 5C (lines 948-950) and 5D (lines 958-966).
19- We added the legends of new Figure 7 (lines 992-1003).

<u>Referee #1:</u>

The study investigates the molecular mechanism of lipid transfer during autophagosome formation, focusing on the interaction between Atg2 and Atg18 in yeast. The cryo-EM structure of the complex between Atg2 and Atg18 is reported. Consistent with previous findings, Atg2 was found to adapt a tube-like shape with a hydrophobic cavity filled with lipids densities that correspond to lipids. Atg18 interacts with Atg2 via a critical interface involving Atg2 residues 922-934 and Atg18 blades 2 and 3. This interaction strongly increases lipid transport by Atg2 in the presence of PI3P, but not in the absence of PI3P. This suggests that Atg18 requires PI3P to support Atg2 function, which is consistent with the requirement of PI3P for Atg18 binding and its proper orientation on membranes.
The lipid transfer assay also shows that Atg9 increases lipid transport of Atg2 alone, or in presence of Atg18, in a PI3P independent manner. Consistent with the observed interaction interface between Atg2 and Atg18, deletion of Atg2 residues 922-934 disrupts its interaction with Atg18, impairing Atg18 recruitment to the PAS and abolishing Atg18-mediated stimulation of lipid transfer. This mutation is also deficient in promoting phagophore elongation and autophagy progression. Based on the experimental data, a model is proposed in which Atg2 receives lipids from the ER and interacts with Atg9 trimers at the phagophore. Atg18 binds to Atg2 in a PtdIns3P-dependent manner, gating the lipid channel and enabling efficient lipid transfer for phagophore expansion.

Overall, this study is of great significance for the autophagy field and beyond. The described function of Atg18 as a gatekeeper of Atg2 is exciting and new. There are, however, some fundamental concerns that need to be addressed before a recommendation on the publication of the manuscript in the EMBO journal can be made.

1. A major concern is the orientation of Atg18 in the complex structure. Compared to the structure of the human

ATG2/WIPI4 complex, Atg18 is twisted by 46{degree sign}, which would detach Atg18 from the membrane, since its PI3P and membrane binding sites are oriented such that an interaction with membranes becomes impossible. The reason for this movement and the biologic significance of this orientation remains unclear. This becomes even more relevant as the binding site between Atg18 and Atg2, involving Atg2 residues 922-934, is located in a loop region of Atg2, suggesting that this interaction could be maintained even if Atg18 had another orientation relative to Atg2. The question of the biological relevance of the observed structure needs to be evaluated to strengthen the conclusions of the manuscript. A possibility would be to show that the orientation of Atg18 on membranes is maintained as seen in the structure of the soluble complex.

We thank the reviewer for the detailed analysis of our data. As the reviewer indeed pointed, the orientation of Atg18 is shifted 46° in respect to the position of WIPI4 reported in complex with ATG2A; however, the orientation of phosphoinositide-binding sites in Atg18 still remains in a position allowing their interaction with the membrane surface. We have assigned the residues of Atg18 mediating phosphoinositide binding, based on amino acid sequence and structural alignments (Figure EV3) to the PROPPIN Hsv2 reported by Krick et al. PNAS 2012, Watanabe et al. JBC 2012 and Baskaran et al. Mol Cell 2012. We highlighted the putative phosphoinositide-binding motif (FRRG) in our structures in Figure 2E, and the binding pockets (Figure EV3), for more clarity. The sites, including the conserved R285 and R286, previously shown to be necessary for autophagy and for Atg18 membrane binding (Krick et al., 2006, Krick et al., 2012), face the same direction as the Atg2 cavity. Thus, we expect that the interaction of the reported Atg2-Atg18 complex with the membrane still occurs. We believe that differences between Atg2-Atg18 and ATG2A-WIPI4 might reflect different conformations of the complexes, and indeed, we do not exclude rearrangements of the complex when associated to a membrane and interacting with other proteins. For this reason, we analyzed the compatibility of our structure with membrane binding by MD simulations. We observed that the resolved complex stably binds a lipid bilayer, establishing interactions with PtdIns3P lipids via Atg18, and with the Atg2 tunnel oriented towards the bilayer in both, flat or highly curved model membranes (Figures 7B and 7C). The simulations also showed that the complex assembled in the conformation that we have uncovered, is capable to bend the lipid bilayer in proximity to the Atg2 cavity allowing the lipid exchange. Thus, the MD simulations show that the resolved structure is compatible with the biological lipid transfer function of the complex. These results are now incorporated in Figures 7, EV3 and EV5, and described in the main text (lines 163-168 and lines 273-317).

2. Given the limitation of the interpretation of the Atg18/Atg2 complex structure, the correlation between functional assay (Figure 1) and the structure is difficult. In particular, the stimulation of the lipid transfer capacity of Atg2 by Atg18 was shown to strictly depend on the presence of PI3P, which is inconsistent with the observed orientation of Atg18 in the complex. This contradiction needs to be resolved by providing experimental evidence. For example, it could be demonstrated that Atg18 undergoes a conformational change if it engages Atg2 after it has been recruited to the membrane in an PI3P dependent manner.

The concern regarding the orientation of Atg18 was addressed (Figures 8, EV3 and EV5) and explained in our rebuttal to point #1. Our MD results show that the resolved structure can bind membranes and indeed bend the bilayer towards the hydrophobic cavity of Atg2, providing the hydrophobic continuity needed for the transfer of lipids (Figure 8B).

3. Furthermore, the orientation of Atg18 appears to induce a steric clash of the membrane binding region of Atg2, in particular the entrance of the lipid transport channel, with Atg9. The model in Figure 7 is not very informative, but MD simulations could be carried out to show that the complex indeed interacts with membranes in a way shown in the cartoon and that the tunnel opening is in direct contact with Atg9 and in close proximity to membranes.

In addition to our MD showing the Atg2 tunnel in proximity to membranes (Figure 8), we have modelled the Atg2-Atg18 complex is association with the Atg9 trimer using AlphaFold3 (Figure EV4C). In the predicted complex, the Atg2-Atg18 dimer adopts a conformation very similar to the one that we resolved by cryo-EM. Moreover, the Atg2-Atg18 complex binds the lateral side of the Atg9 trimer, allowing its simultaneous interaction with the membrane via both Atg2 and Atg18, with the Atg2 C-terminal gate of the lipid transport channel facing the lipid bilayer. In contrast to the previously reported model for Atg2-Atg9 complex (van Vliet et. al Mol Cell. 2022), our model does not predict a direct contact of the Atg2 tunnel with Atg9 trimer. In both cases, however, the regions of interactions involve the C-terminal fragment of Atg2, including alpha-helical domains located there (Figure EV4C, magenta colored region), which are opposite to the binding region that we identified for Atg18, and thus generate no interference for simultaneous Atg18-Atg2-Atg9 interaction.

We have added the Atg9 trimer-Atg2-Atg18 structural model in Figure EV4C to illustrate these interactions. We have modified the manuscript text accordingly on lines 245-250, 397-402.

4. Another major limitation of the study is the missing structure of N-terminal part of Atg2. The argument that structural flexibility and biased orientation is a reason that this part of Atg2 is not resolved is not entirely conclusive. The beta-sheet structure is usually quite stable suggesting that the overall structure of Atg2 should be quite stable as well. Moreover, a biased orientation of Atg2 should not only affect the C-terminal part, but the overall structure, since Atg2 is a long rod-shaped protein. Can the authors provide a more convincing argumentation? They could, for example, determine the structural flexibility of Atg2 by NMR or hydrogen exchange experiments.

We acknowledge that the N-terminal part of Atg2 is poorly resolved in our structure. We ran a 3D variability analysis on the final map using the cryoSPARC software, and demonstrated movements confined to the N-terminal part of Atg2 in one of the analysis using eigenvectors of the 3D covariance. Our cryo-EM structure appears to suffer from continuous heterogeneity in the N-terminus, which limits the resolution there. The reasons for that could possibly be the absence of the membrane and/or the binding partner that might stabilize the N-terminal part of Atg2 associated with the ER/ERES. We also plotted the pLDDT score on the AlphaFold3 model of the Atg2-Atg18 complex, This analysis revealed lower confidence (50% or less) in the Atg2 N-terminal part, suggesting that the structure of this region might not be extremely stable in physiological conditions or become so only as part of a complex. We have added Figure S3 to address the Atg2 N-terminal instability and modified the text accordingly on lines 132-138.

Referee #2:

Ramirez report an atomically interpretable structure of the yeast Atg2-Atg18 complex and in vitro and in vivo functional assays of the main Atg18-binding loop of Atg2. This is a very straightforward exposition of a structure corroborated by the obvious assays, and the conclusion is clear and internally consistent. In some ways this is a stronger study than the related papers by on the ATG2A:WIPI3/4 interaction by Wang et al. NSMB (2025) and Ren et al. N. Comms. (2020), which did not report functional assessment of mutations in ATG2A and thus left a gap. In principle the contribution merits publication in EMBO J but substantial improvements are needed.

The structure suffers from substantial preferred orientations and an unhealthy-looking FSC. The map quality is at the borderline where side-chain assignments can be trusted. Given an Alpha Fold starting model, it is probably OK. The clash score is passable although not great.

1- I note that the Wang NSMB paper on ATG2A suffered even more, and symptoms were apparent even in the Kang 2025 Nature paper on LPD-3. I don't know how much more it can be improved, but it would be worth at least noting this issue in the results section.

We agree with the reviewer that large parts of the cryo-EM map have poor resolution, especially towards the N-terminal region of Atg2. This is probably due to a couple of reasons. First, as mentioned by the reviewer, the sample suffered from preferred orientation, probably due to the extended shape of the Atg2-Atg18 complex. Second, the N-terminal part of Atg2 is predicted to be unstructured at least in our preparations (as also pointed out by Reviewer 1). During our cryo-EM data processing, we have tried different approaches to improve the quality of the map, including extensive particle classifications in 2D and 3D, as well as rebalancing of particle angular view distribution using the tools available in cryoSPARC, reaching no improvement. We have discussed this issue in the revised manuscript on lines 132-138.

2- Fig. 2F should show the side chains in the 922-934 sequence, not just the main chain trace.

We have displayed the side chains in revised Fig. 2F as requested.

3- One major issue is that the nature and identity of the functionally relevant WIPI binding sites in human vs yeast ATG2A. The authors should read and cite the paper by J. Ren et al in N. Comms. (2020). The binding site on the WIPI side in the present work and Ren. On the other hand, Ren et al. implicate the Sc Atg2 sequence

1193-PWPHSSPMF-1201 as the Atg18 binding site, which does not match even the secondary (non-functional) 1149-1157 sequence. Is there some chance the 1149-1157 sequence in this paper was mis-assigned, or did Ren get it completely wrong? This should be clarified with additional figures showing structural superpositions of the current structure and Ren's ATG2A-WIPI3 complex, and detailed density for the 1149-1157 sequence showing side chains as docked in density and (if the density alone is insufficient to justify the assignment), also the Alpha Fold metrics.

We thank the reviewer for stressing this critical point and agree on addressing it in our manuscript. As a first step, we made the sequence alignment described in Ren et al. 2020 have a broader view of the consensus sequences among the Atg2 homologous. We performed our analysis using MAFFT (Katoh et al. Nucleic Acids Res. 2002, Katoh and Standley. Mol Biol Evol. 2013) and found that the region 1193-1201 was not aligned with the WIR-motif of human ATG2A described in Ren et al. (Figure R1B_for the reviewer). We also tested the alignment in the default setting provided by UNIPROT (Clustal omega alignment), as the results may differ according to the software used. However, also in this case, we found no alignment of this region (Figure R1A_for the reviewer). Unfortunately, the software and alignment conditions used by Ren and co-workers are not described, and thus we cannot explain why our alignment results do not match those reported. We nevertheless located the 1193-1201 region in our structure and identified the regions in Atg18 that would bind the WIR-motif. We observed that the 1193-1201 fragment is positioned in the opposite site of the uncovered Atg2-Atg18 interaction interface and seems to not play a role in the contacts between these two proteins (Appendix Figure S3B). We also located the WIR-binding sites in Atg18 according to the sequence alignment with WIPI3 and other PROPPINs, and found that the pockets are located in the close proximity of the 922-934 region that we identified as the specific binding region in Atg2 (Appendix Figure S3). We do not think that 1149-1157 is a major interaction region, as deletion of the complete stretch has not impacted autophagy. The AlphaFold3 pLDDT score for this fragment is close to 50%, indicating its structural instability, however it is flanked by beta-strands predicted with high confidence (see Figure R2, left panel), allowing accurate model building. The cryo-EM map region covering 1149-1157 is of poorer quality probably due to higher flexibility of this loop, as also predicted by AlphaFold. However, it still allows to fit the sequence, showing that the interaction with Atg18 is unlikely in the determined configuration of the complex (see Figure R2, right panel, 1149-1157 colored chartreuse). We speculate that the region 922-934 might resemble the WIR-motif described for WIPI3 and the other homologous in higher eukaryotes, although we are aware that this region does not fully match the characteristics proposed for the WIR-motif, nor show high conservation among fungus.

Proposed WIR-motif: xxx(Φ)x(Φ)xxx(φ)F >> Sc Atg2: -922-QLKNPIDVFKDID-934

We have addressed these observations in our manuscript and discussed the results accordingly (lines 155-163, and lines 329-332).

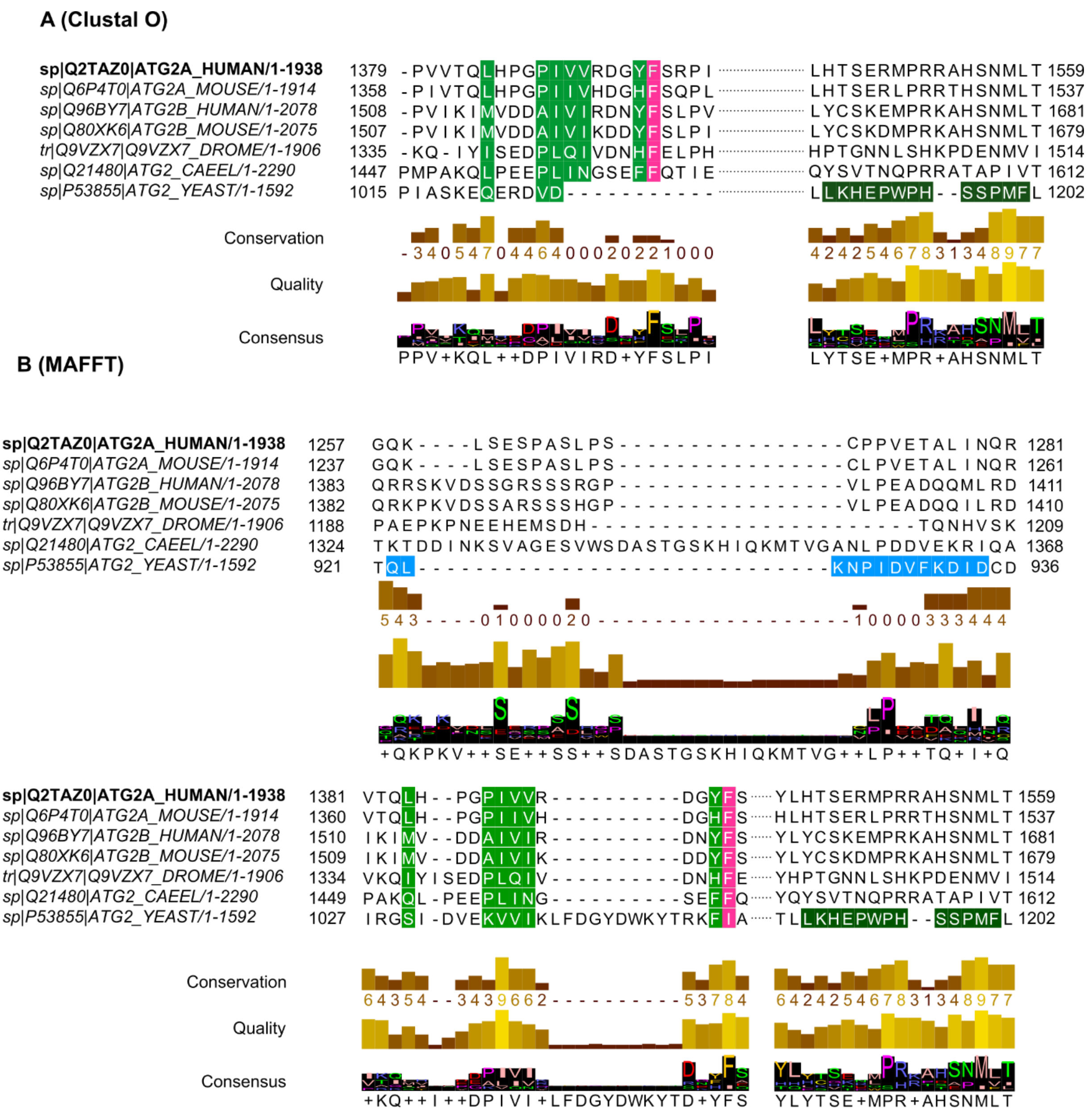

Figure R1 (for the reviewer). Amino acid sequence alignment of various ATG2 proteins performed with the Clustal O (A) or MAFFT (B) software. The WIR-motif, according to the ATG2A sequence, is highlighted in green with the conserved Phe highlighted in pink. The 1193-1201 region in yeast Atg2 is highlighted in dark green. The 922-934 region is highlighted in blue.

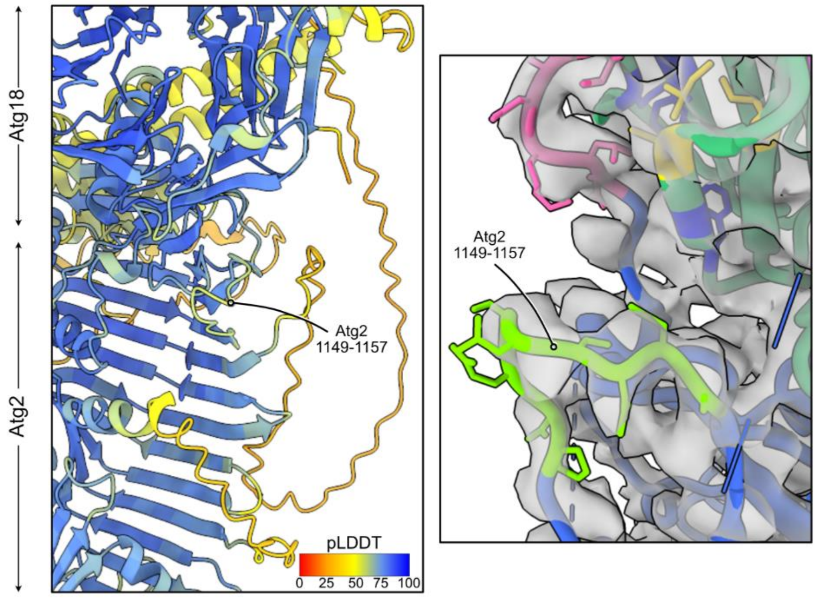

Figure R2 (for the reviewer). Model of the interaction of Atg18 and Atg2. Shown is region 1149-1157 in Atg2, whose deletion does not impair autophagy. The AlphaFold3 pLDDT score for this fragment is close to 50%, indicating its structural instability. It is flanked by beta-strands predicted with high confidence (left panel), allowing accurate model building. The cryo-EM map region covering 1149-1157 (right panel) is of poorer quality probably due to higher flexibility of this loop, as also predicted by AlphaFold.

4- The superposition with the Wang structure is shown as aligned based on the ATG2 portion, but the WIPI4/Atg18 comparison should also be carried out. The structural alignment of the Atg2 and ATG2A should be shown and the sequences written out and aligned on the basis of the WIPI-driven structural alignment, and the results discussed relative to Ren et al and relative to the functional data.

We have updated the structures comparison in Figure EV2, where we added panels D and E, which align the complexes based on the Atg18/WIPI4 overlay. While in both cases the blade 2 of the beta-propeller of both Atg18 and WIPI4 is involved in the interaction with Atg2 and ATG2A, respectively, the binding interfaces on these blades are different. In the WIPI4-ATG2A structure (Wang et al. 2025, PDB 8KBX), the contact between the proteins is mostly established via the residues 87-92 of WIPI4, which are outside of the interaction sites reported for WIPI3 by Ren et al. 2020. We have revised the manuscript accordingly on lines 145-148, 350.

5- The use of the word "gates" in the title implies to me that Atg18 regulates the opening of the Atg2 lipid channel relative to the unbound Atg2 protein. The authors don't show this, however. The word "targets" would be more honest.

We changed the title into *Atg18 interaction positions Atg2 for efficient lipid transfer into phagophore elongation*, to reflect more our findings.

6- Overall, the discussion and Fig. 7 are overly focused on restating various speculative opinions in the field relating to gating and the role of Atg9. I recommend removing Fig. 7 which shows a tight complex between Atg2 and Atg9 that is backed up by no data that I am aware of, and certainly not supported by the data in this manuscript. Refocus the discussion on the comparative structural analysis of the various Atg2/ATG2A plus Atg18/WIPI3/4 structures now available.

The interaction of Atg2 and Atg9, and the assembly of the complex was previously shown by van Vliet et al. Mol Cell. 2020. These authors propose a model of interaction through the C-terminal region of Atg2, which our AlphaFold3 prediction of the complete Atg2-Atg18-Atg9 complex also identified as the interaction region between Atg2 and Atg9 (Figure EV4C, magenta highlight). Although we did not further explore the structure of

the complete complex, we consider that it is important to illustrate that the interface identified between Atg2-Atg18 and its structural arrangement is compatible with the interaction with Atg9.

7- Watanabe is correctly cited for pointing out the Atg18 residues important for Atg2 interaction, however, the authors should still cite the original Hsv2 structure papers that predated Watanabe, from Baskaran et al 2012 and Krick et al 2012.

We agree and thank the reviewer for this observation. We included the corresponding citations in our introduction and results sections.

Referee #3:

The manuscript by presents a structural and functional analysis of the yeast Atg2-Atg18 complex, a key lipid transfer module at the ER-phagophore membrane contact site during macroautophagy. Using cryo-electron microscopy, the authors determine the structure of Atg2 in complex with Atg18 and characterize the Atg18-binding interface in Atg2 that is required for Atg18 recruitment and lipid transfer activity. They visualise continuous lipid densities along the internal hydrophobic cavity of Atg2, providing direct structural evidence that the Atg2 channel is filled with lipids. Complementary in vitro and in vivo experiments support a model in which Atg18 positions and gates the Atg2 lipid channel to promote directional lipid flux and efficient phagophore elongation during both bulk and selective autophagy. Overall, the manuscript is very nice with strong structural and functional data; the in vitro and in vivo validation makes a compelling case for the importance of the newly identified Atg2-Atg18 interface in lipid transfer-mediated phagophore expansion. The manuscript is well written, with a concise and clear text.

Major points

1- Why have the authors not introduced point mutations (e.g. alanine substitutions) into the Atg18-Atg2 interface instead of deleting such a long stretch of Atg2? Removing residues 922-934 may have broader functional consequences and could affect Atg9 binding. The authors should test whether the Atg2Δ922-934 mutant can still bind Atg9 and, if not, design alternative Atg18-Atg2 interface mutants that more selectively disrupt Atg18 binding.

The identified 922-934 region is located in a loop region. Thus, we hypothesized that a complete deletion might not affect the overall structure of the protein and we could use it to specifically examine the relevance of the Atg2-Atg18 binding in autophagy. However, we agree with the reviewer that such deletion could cause additional effects in the interaction with other Atg proteins. For this reason, we tested the interaction between the mutant Atg2 and Atg9 as required. We also performed pull-down experiments in autophagy inducing conditions (i.e., nitrogen starvation), and observed that the mutant Atg2 associated with Atg9, indicating that the deletion of the region 922-934 does not abolish the interaction between the proteins (Figure EV4A and EV4B), which indeed is higher than with the wild-type version. We speculate that the higher detection of the mutant in our pull-downs might be due to the block in the progression of the autophagosome formation in those cells, which could lock the complex allowing its detection. We included this result in Figure EV4 with the corresponding citation in the results section (lines 239-245).

2- For key experiments, the authors use a HaloTag on endogenous Atg18, a GFP tag on Atg2 and mCherry on Atg8. They should demonstrate that autophagy is not significantly affected by these tags. Pho8Δ60 assays should be included for the relevant tagged constructs to directly address this point.

We agree with the reviewer. The fluorescent tags that we chose to track the Atg proteins are widely used to monitor autophagy *in vivo*, but we agree that an analysis of functionality of these fusion proteins is missing in this work (but presented in previous ones). We have followed the recommendation of this reviewer and tested the functionality of the Atg18-Halo, mCherry-Atg8 and Atg2-GFP chimeras using the Pho8Δ60 assay. We have included a strain simultaneously expressing these three fusion proteins to assess autophagy functionality in the conditions used in Figure 5. We found that the sole expressing of Atg2-GFP or Atg18-Halo does not affect the autophagic flux, while the expression of mCherry-Atg8 resulted in approx. 50% reduction of Pho8Δ60 activity (Appendix Figure S4A). Importantly, simultaneous expression of the three tagged proteins did not further increase the impairment due to mCherry-Atg8. Although the reduction is significant, it might reflect a delay in the autophagy flux and is not a complete block. Given that the analysis of experiments showed in Figures 3A, 3B and 5 compare differences between Atg2 variants expressed in the same background, the interpretations of our results are still valid. However, we reported this control in our results section on lines 182-186, as we consider

important to point that the mCherry tagging of Atg8 should be carefully used to monitor autophagy and results must be validated by other methods.

Minor points

3- For the cryo-EM structure, the authors should map the local resolution onto the density map (and include this as a supplementary figure) and briefly describe in the main text how the atomic model was built into the map.

The local resolution map is shown in Figure EV1C. The details of the atomic model building are provided in the methods section under “Model building and refinement”, at page 16-17 in the revised manuscript.

4- Can Atg9 and Atg18 bind Atg2 simultaneously, or are these interactions mutually exclusive? Could the authors use their system together with recombinant Atg9 to address these questions experimentally?

We speculate that the interactions are not mutually exclusive, as the binding interface between Atg2 and Atg18 locates opposite to the alpha-helices in the C-terminal of Atg2, previously described to mediate the interaction with Atg9 (van Vliet et. al. Mol Cell. 2022). Our AlphaFold3 prediction of the complex composed byAtg2, Atg18 and the Atg9 trimer, confirms this (Figure EV4C) and shows that the structure described in our work is compatible with a concomitant interaction of Atg2 with both Atg18 and the Atg9 trimer. We are aware that only binding experiments might provide a direct answer. However, we have not been able to optimize the interaction of the purified proteins yet and thus we have not been able to validate these observation biochemically. We speculate that the presence of detergent required for Atg9 purification might influence the binding or we are missing and additional interactor required to promote binding. Thus, the outcome of those experiments goes beyond what we aim to present in this work. Nevertheless, we appreciate the concern of the reviewer and therefore we have included this point in our discussion (lines 397-402).

5- In Fig. 4A and 5C, the authors should clarify how mCherry-Atg8 and Atg2-GFP were expressed. Were the endogenous proteins tagged at the genomic locus, or were tagged versions overexpressed in the presence of wild-type proteins?

We thank the reviewer for this observation and we clarified this aspect in the legends of Figures 4A and 5C.

6- The authors should explain why they chose GFP-tagged constructs, which can dimerise, rather than using monomeric EGFP-tagged constructs.

The tagging of Atg2 and other Atg proteins with GFP has used in our previous *in vivo* experiments (Gomez-Sanchez JCB, 2018, Chumpen Ramirez and Gomez Sanchez, Autophagy, 2023). In order to maintain consistency between our observations, we performed the assays using the same tag. Although the possibility of dimerization exists, this seems not to lead to the deleterious effect that we observed with the 922-934 mutant nor rescue the activity of the 1149-1157 deletion, as we obtained identical results with untagged and tagged proteins (see our answer to point # 7 of this reviewer and Appendix Figure S4A).

7- The constructs used for the Pho8Δ60 experiments in Fig. 3C need to be more clearly specified in both the figure and the legend. If the comparison is between untagged wild-type Atg2 and a GFP-tagged Atg2 mutant, the authors should also include wild-type Atg2-GFP. Conversely, if all constructs are GFP-tagged, untagged Atg2 (ideally both wild type and mutant) should be included to disentangle the effect of the GFP tag from the mutation.

Thank you to point this out; this has been clarified in the main figures and their legends. We also adjusted the labels in Figure 3B to maintain consistency and avoid confusions. Given that many of our analysis include the GFP tagged versions of Atg2, we choose these constructs to perform our Pho8D60 assay using the tagged proteins. However, we agree with the reviewer that the effect the GFP tag on Atg2 should be addressed here as well. Thus, we included this control in Appendix Figure S4A. In agreement with identical verification done in previous publications, our data shows that GFP-tagging of Atg2 does not impair the functionality of this protein, strongly suggesting that the autophagy block observed in cells expressing Atg2$^{D922-934}$-GFP is due to the introduced truncation (Figure 3C). In order to rule out that this Atg2 mutant is particularly sensitive to the fusion with GFP, we back-transformed the Pho8D60 *atg2D* strain with untagged versions of Atg2, Atg2$^{D922-934}$ or Atg2$^{D1149-1157}$ under the control of the endogenous promoter and performed the Pho8D60 assay. As observed with the tagged proteins, only the deletion of 922-934 region in Atg2 impaired the autophagy flux, demonstrating that

the defect observed with the fusion protein is not due to the tag. This experiment has been included in Appendix Figure S4B and mentioned in the results section on lines 193-197.

8- In Fig. 4A it would be helpful to include an example of a giant Ape1 structure that is similar in size to the example shown for wild-type Atg2. In addition, the authors should quantify the diameter of the giant Ape1 structures for all 27 cells and compare these measurements between the Atg2 wild-type and mutant datasets, to rule out the possibility that systematically smaller Ape1 structures in the mutant condition bias the analysis.

We thank the reviewer for this observation and replaced the images in Figure 4A by representative phagophores with similar size. Additionally, we analyzed our data again, this time including the measurement of the diameter and the area of the Ape1 cargo structures corresponding to each of the analyzed phagophores. The data corresponding to the phagophores was replaced in Figure 4C and is accordingly indicated in the manuscript on lines 206-209. The new data about giant Ape1 cargo are added in Appendix Figure S5. Our results confirmed that there are no significant differences in the size of the cargoes expressed in both strains that could bias the analysis of the phagophore sizes (Appendix Figure S5A-B). Nonetheless, to completely rule out this possibility, we analyzed the correlation between the areas of the Ape1 structure and the phagophore, or the phagophore elongation in cells expressing Atg2-GFP or Atg2$^{D922-934}$-GFP. We found that the area of the phagophore (Appendix Figure S5C), nor its elongation (Appendix Figure S5D) linearly correlated with the area of the giant Ape1 cargo, indicating that the phagophore elongation does not depend on the cargo size. These observations are now included in our results section on lines 210-215.

9- In Fig. 5D, the authors use an unpaired two-tailed t-test. Given that the data do not appear to be normally distributed, is a t-test the most appropriate statistical test here? The authors should justify the use of the t-test or consider using an alternative.

We thank the reviewer for this observation. We tested our data for normality and corroborated that the data from the plots showed in Figures 5 D-E do not follow a normal distribution. Although some authors consider that parametric tests are not very sensitive to discrete deviations from normality, it seems that there is not to be a clear consensus about how much deviation can be tolerated (McDonald, J.H. 2014. Handbook of Biological Statistics (3rd ed) Sparky House Publishing, Baltimore, Maryland). Thus, we adjusted our statistical analysis to the non-parametric Mann-Whitney test for two sample comparisons (Figures 5D and 5F), or Kruskal-Wallis test for multiple comparisons (Figures 5E). The new analysis showed a similar result than before, and therefore our conclusions remain unchanged. The p-values are now indicated in the main figure.

Dear Christian,

We have now received re-review reports from three referees, which I have included below. As you will see, you have addressed their concerns satisfactorily. Before I can finally accept the manuscript, there are some remaining editorial points which need to be addressed. In this regard would you please:

- acknowledge the following funding in our online submission system: missing funding info in eJP: Lundbeck Foundation (R383-2022-180) grants. S.V. acknowledges support from the Swiss National Science Foundation (grant IDs 310030_219264),
- include a "Disclosure and competing interests statement" that declares employment in a biotech company,
- remove the AC/CrediT section from the text,
- list callouts in the manuscript text sequentially; there are currently callouts missing for Fig. 6A-D,
- place EV figure legends in the main manuscript below the main figure legends,

save the appendix file in PDF format with all Appendix figures inside, instead of uploading individually; the title page should contain "Appendix for Atg18 interaction positions Atg2 for efficient lipid transfer into phagophore elongation" and table of contents with the page numbers for the listed items; the nomenclature should be Appendix Figure Sx and Appendix Table Sx throughout the main manuscript file and Appendix PDF,
- ensure full datasets (PDB: 9T0C, EMBD: EMD-55395, Zenodo: 10.5281/zenodo.18824109, BioImage Archive: S-BIAD2987) are publically accessible,
- define the annotated p values ****/***/**/* as well as provide the exact p-values for the same in the legend of figure EV 4b as appropriate,

provide the exact p values in the legends of figures 1c; 3b, c; 6d; S 4a, b,
state the statistical test used for data analysis in the legends of figure EV 4b,
- define error bars in the legends of figure EV 4b,
- define the measure of centre for the error bars in the legends of figures 6b, d; and S6b, d,
- define the white dotted lines in the legends of figures 3a; 4a; 5c; 6a, c; S6a, c,
- define the yellow dotted lines in the legends of figures 4c,
- rename the "Materials and methods" section as "Methods", and
- use the following section order: Title page - Abstract - Keywords - Introduction - Results - Discussion - Methods - Data Availability - Acknowledgements - Disclosure and Competing Interests Statement - References - Figure Legends - Table(s) - Expanded View Figure Legends.

We include a synopsis of the paper (see http://emboj.embopress.org/). Please provide me with a general summary image, a two sentence statement and 3-5 bullet points that capture the key findings of the paper.

I am looking forward to receiving your revised manuscript.

EMBO Press is an editorially independent publishing platform for the development of EMBO scientific publications.

Best wishes,

William

William Teale, PhD
Senior Editor
The EMBO Journal
w.teale@embojournal.org

Instructions for preparing your revised manuscript:

Read our guidance for manuscript revisions and related editorial policies: https://link.springer.com/journal/44318/submission-guidelines#cms-Revised-submissions

Please also check that the title and abstract of the manuscript are brief, yet explicit, even to non-specialists.

When assembling figures, please refer to our figure preparation guideline in order to ensure proper formatting and readability in print as well as on screen:
https://media.springernature.com/original/springer-cms/rest/v1/content/27825798/data/v1

IMPORTANT: When you send the revision we will require

- a point-by-point response to the referees' comments, with a detailed description of the changes made (as a word file).
- a word file of the manuscript text.
- individual production quality figure files (one file per figure)
- a complete author checklist
- Expanded View files (replacing Supplementary Information)
- a Reagents and Tools Table as part of the Methods section

Please remember: Digital image enhancement is acceptable practice, as long as it accurately represents the original data and conforms to community standards. If a figure has been subjected to significant electronic manipulation, this must be noted in the figure legend or in the 'Methods' section. The editors reserve the right to request original versions of figures and the original images that were used to assemble the figure.

We realize that it is difficult to revise to a specific deadline. In the interest of protecting the conceptual advance provided by the work, we recommend a revision within 3 months (30th Jun 2026). Please discuss the revision progress ahead of this time with the editor if you require more time to complete the revisions. Use the link below to submit your revision:

https://emboj.msubmit.net/cgi-bin/main.plex

Referee #1:

The authors addressed all my concerns. The manuscript has significantly improved. The MD simulations revealed very interesting insights into the molecular mechanism of membrane interaction and function of the Atg2/Atg18 complex. The findings are significant and relevant for a broad readership and I highly recommend the publication of the manuscript in its current form. I congratulate the authors to this exciting study.

Referee #2:

This is a terrific revision and should be published with high priority.

Referee #3:

We would like to thank the authors for addressing our revision requests! We are satisfied that these points have been appropriately addressed and we have no further comments.

We have now received re-review reports from three referees, which I have included below. As you will see, you have addressed their concerns satisfactorily. Before I can finally accept the manuscript, there are some remaining editorial points which need to be addressed. In this regard would you please:

1 - acknowledge the following funding in our online submission system: missing funding info in eJP: Lundbeck Foundation (R383-2022-180) grants. S.V. acknowledges support from the Swiss National Science Foundation (grant IDs 310030_219264)

2- include a "Disclosure and competing interests statement" that declares employment in a biotech company

3 - remove the AC/CrediT section from the text.

- *We removed the section Authors Contributions from the text.*

4 - list callouts in the manuscript text sequentially; there are currently callouts missing for Fig. 6A-D,

- *We included the callouts of Figures 6A-D in result sections, lines 257-258.*

5- place EV figure legends in the main manuscript below the main figure legends

- *We did this as required.*

6 - save the appendix file in PDF format with all Appendix figures inside, instead of uploading individually; the title page should contain "Appendix for Atg18 interaction positions Atg2 for efficient lipid transfer into phagophore elongation" and table of contents with the page numbers for the listed items; the nomenclature should be Appendix Figure Sx and Appendix Table Sx throughout the main manuscript file and Appendix PDF.

- *We did this as required*.

7 - ensure full datasets are publically accessible:

- PDB: 9T0C, EMBD: EMD-55395 > *the PDB and EMDB entries will be automatically released as soon as the paper is published according to the PDB release status*.
- Zenodo: 10.5281/zenodo.18824109 > *This data is already accessible*.
- BioImage Archive: S-BIAD2987. > *This data is already accessible*.

8 - define the annotated p values ****/***/**/* as well as provide the exact p-values for the same in the legend of figure EV 4b as appropriate.

- *The significance level of the p values was included in the Methods section (line 645) and the exact p values were included in the figure legends as required (lines 1145-1148).*

9 - provide the exact p values in the legends of figures 1c; 3b, c; 6d; S 4a, b,

- *The exact p values were provided in the corresponding figure legends as required.*
- *In order to maintain the same format of the figures, we replaced the exact p values shown in Figures 3B, 3C, S4A, and S4B by the significance levels (stars). Figure 3 is newly provided with*

these changes. Changes in Appendix Fig S4 are included in the new Appendix document. The significance levels are accordingly described in the Methods section.

- *In the case of Figure 6, the exact p values were previously provided in the figure legends. Thus, in order to avoid confusions, the significance levels were removed from the legend and provide it in the methods section.*

10 - state the statistical test used for data analysis in the legends of figure EV 4b, define error bars in the legends of figure EV 4b

- *This information was included as required (lines 1145-1147)*

11 - define the measure of centre for the error bars in the legends of figures 6b, d; and S6b, d,

- *This information was included in the corresponding figure legends as required (line 1058 of the main text, and Appendix Fig S6).*

12 - define the white dotted lines in the legends of figures 3a; 4a; 5c; 6a, c; S6a, c, define the yellow dotted lines in the legends of figures 4c.

- *This information was included in the corresponding figure legends, as required.*

13 - rename the "Materials and methods" section as "Methods".

- *We renamed the section as required*.

14 - use the following section order: Title page - Abstract - Keywords - Introduction - Results - Discussion - Methods - Data Availability - Acknowledgements - Disclosure and Competing Interests Statement - References - Figure Legends - Table(s) - Expanded View Figure Legends.

- *We organized the manuscript as required*.

15- We include a synopsis of the paper (see http://emboj.embopress.org/). Please provide me with a general summary image, a two sentence statement and 3-5 bullet points that capture the key findings of the paper.

Dear Christian,

I am pleased to inform you that your manuscript has been accepted for publication in the EMBO Journal.

Congratulations to your team and all of the groups involved!

Your manuscript will be processed for publication by EMBO Press. It will be copy edited and you will receive page proofs prior to publication. Please note that you will be contacted by Springer Nature Author Services to complete licensing and payment information.

You may qualify for financial assistance for your publication charges - either via a Springer Nature fully open access agreement or an EMBO initiative. Check your eligibility: https://link.springer.com/journal/44318/how-to-publish-with-us

Should you be planning a Press Release on your article, please get in contact with embo_production@springernature.com as early as possible in order to coordinate publication and release dates.

If you have any questions, please do not hesitate to contact the Editorial Office. Thank you for your contribution to The EMBO Journal.

Best wishes,

William

William Teale, PhD
Editor
The EMBO Journal
w.teale@embojournal.org

*****
Please note that it is The EMBO Journal policy for the transcript of the editorial process (containing referee reports and your response letters) to be published as an online supplement to each paper. If you should prefer removal of any referee-only figures included in the point-by-point response(s), e.g. because they may still be used for future publication or because they have been reproduced from published work by others, please do let us know immediately via response email.
More information is available here: https://link.springer.com/partners/embo-press/editorial-policies#Peer%20review

** Click here to be directed to your login page: https://emboj.msubmit.net

EMBO Press Author Checklist

Corresponding Author Name: Christian Ungermann	
Journal Submitted to: The EMBO Journal	
Manuscript Number: EMBOJ-2025-122766	

USEFUL LINKS FOR COMPLETING THIS FORM
The EMBO Journal - Author Guidelines
EMBO Reports - Author Guidelines
Molecular Systems Biology - Author Guidelines
EMBO Molecular Medicine - Author Guidelines

Reporting Checklist for Life Science Articles (updated January
This checklist is adapted from Materials Design Analysis Reporting (MDAR) Checklist for Authors. MDAR establishes a minimum set of requirements in transparent reporting in the life sciences (see Statement of Task: 10.31222/osf.io/9sm4x). Please follow the journal's guidelines in preparing your
Please note that a copy of this checklist will be published alongside your article.

Abridged guidelines for figures
1. Data
The data shown in figures should satisfy the following conditions:
- the data were obtained and processed according to the field's best practice and are presented to reflect the results of the experiments in an accurate and unbiased manner.
- ideally, figure panels should include only measurements that are directly comparable to each other and obtained with the same assay.
- plots include clearly labeled error bars for independent experiments and sample sizes. Unless justified, error bars should not be shown for technical
- if n<5, the individual data points from each experiment should be plotted. Any statistical test employed should be justified.
- Source Data should be included to report the data underlying figures according to the guidelines set out in the authorship guidelines on Data

2. Captions
Each figure caption should contain the following information, for each panel where they are relevant:
- a specification of the experimental system investigated (eg cell line, species name).
- the assay(s) and method(s) used to carry out the reported observations and measurements.
- an explicit mention of the biological and chemical entity(ies) that are being measured.
- an explicit mention of the biological and chemical entity(ies) that are altered/varied/perturbed in a controlled manner.
- the exact sample size (n) for each experimental group/condition, given as a number, not a range;
- a description of the sample collection allowing the reader to understand whether the samples represent technical or biological replicates (including how many animals, litters, cultures, etc.).
- a statement of how many times the experiment shown was independently replicated in the laboratory.
- definitions of statistical methods and measures:
  - common tests, such as t-test (please specify whether paired vs. unpaired), simple χ2 tests, Wilcoxon and Mann-Whitney tests, can be unambiguously identified by name only, but more complex techniques should be described in the methods section;
  - are tests one-sided or two-sided?
  - are there adjustments for multiple comparisons?
  - exact statistical test results, e.g., P values = x but not P values < x;
  - definition of 'center values' as median or average;
  - definition of error bars as s.d. or s.e.m.

Please complete ALL of the questions below.
Select "Not Applicable" only when the requested information is not relevant for your study.

Materials

Newly Created Materials	Information included in the manuscript?	In which section is the information available? (Reagents and Tools Table, Materials and Methods, Figures, Data Availability Section)
New materials and reagents need to be available; do any restrictions apply?	Yes	Materials and Methods

Antibodies	Information included in the manuscript?	In which section is the information available? (Reagents and Tools Table, Materials and Methods, Figures, Data Availability Section)
For **antibodies** provide the following information:<br>- Commercial antibodies: RRID (if possible) or supplier name, catalogue number and or/clone number<br>- Non-commercial: RRID or citation	Not Applicable	

DNA and RNA sequences	Information included in the manuscript?	In which section is the information available? (Reagents and Tools Table, Materials and Methods, Figures, Data Availability Section)
**Short novel DNA or RNA including primers, probes**: provide the sequences.	Not Applicable	

Cell materials	Information included in the manuscript?	In which section is the information available? (Reagents and Tools Table, Materials and Methods, Figures, Data Availability Section)
**Cell lines:** Provide species information, strain. Provide accession number in repository **OR** supplier name, catalog number, clone number, and/**OR** RRID.	Yes	EMBO Reagents and Tools Table
**Primary cultures:** Provide species, strain, sex of origin, genetic modification status.	Not Applicable	
Report if the cell lines were recently **authenticated** (e.g., by STR profiling) and tested for mycoplasma contamination.	Not Applicable	

Experimental animals	Information included in the manuscript?	In which section is the information available? (Reagents and Tools Table, Materials and Methods, Figures, Data Availability Section)
**Laboratory animals or Model organisms:** Provide species, strain, sex, age, genetic modification status. Provide accession number in repository **OR** supplier name, catalog number, clone number, **OR** RRID.	Yes	EMBO Reagents and Tools Table
**Animal observed in or captured from the field:** Provide species, sex, and age where possible.	Not Applicable	
Please detail **housing and husbandry conditions**.	Not Applicable	

Plants and microbes	Information included in the manuscript?	In which section is the information available? (Reagents and Tools Table, Materials and Methods, Figures, Data Availability Section)
**Plants:** provide species and strain, ecotype and cultivar where relevant, unique accession number if available, and source (including location for collected wild specimens).	Not Applicable	
**Microbes:** provide species and strain, unique accession number if available, and source.	Not Applicable	

Human research participants	Information included in the manuscript?	In which section is the information available? (Reagents and Tools Table, Materials and Methods, Figures, Data Availability Section)
If collected and within the bounds of privacy constraints report on age, sex and gender or ethnicity for all study participants.	Not Applicable	

Core facilities	Information included in the manuscript?	In which section is the information available? (Reagents and Tools Table, Materials and Methods, Figures, Data Availability Section)
If your work benefited from core facilities, was their service mentioned in the acknowledgments section?	Yes	Materials and Methods

Design

Study protocol	Information included in the manuscript?	In which section is the information available? (Reagents and Tools Table, Materials and Methods, Figures, Data Availability Section)
If study protocol has been **pre-registered, provide DOI in the manuscript**. For clinical trials, provide the trial registration number **OR** cite DOI.	Not Applicable	
Report the **clinical trial registration number** (at ClinicalTrials.gov or equivalent), where applicable.	Not Applicable	

Laboratory protocol	Information included in the manuscript?	In which section is the information available? (Reagents and Tools Table, Materials and Methods, Figures, Data Availability Section)
Provide DOI OR other citation details if **external detailed step-by-step protocols** are available.	Not Applicable	

Experimental study design and statistics	Information included in the manuscript?	In which section is the information available? (Reagents and Tools Table, Materials and Methods, Figures, Data Availability Section)
Include a statement about **sample size** estimate even if no statistical methods were used.	Yes	EMBO Reagents and Tools Table; Figure legends
Were any steps taken to minimize the effects of subjective bias when allocating animals/samples to treatment (e.g. **randomization procedure**)? If yes, have they been described?	Yes	EMBO Reagents and Tools Table; Figure legends
Include a statement about **blinding** even if no blinding was done.	Not Applicable	
Describe **inclusion/exclusion criteria** if samples or animals were excluded from the analysis. Were the criteria pre-established?<br><br>If sample or data points were omitted from analysis, report if this was due to attrition or intentional exclusion and provide justification.	Not Applicable	
For every figure, are **statistical tests** justified as appropriate? Do the data meet the assumptions of the tests (e.g., normal distribution)? Describe any methods used to assess it. Is there an estimate of variation within each group of data? Is the variance similar between the groups that are being statistically compared?	Yes	EMBO Reagents and Tools Table; Figure legends

Sample definition and in-laboratory replication	Information included in the manuscript?	In which section is the information available? (Reagents and Tools Table, Materials and Methods, Figures, Data Availability Section)
In the figure legends: state number of times the experiment was **replicated** in laboratory.	Yes	EMBO Reagents and Tools Table; Figure legends
In the figure legends: define whether data describe **technical or biological replicates**.	Yes	EMBO Reagents and Tools Table; Figure legends

Ethics

Ethics	Information included in the manuscript?	In which section is the information available? (Reagents and Tools Table, Materials and Methods, Figures, Data Availability Section)
Studies involving **human participants**: State details of **authority granting ethics approval** (IRB or equivalent committee(s), provide reference number for approval.	Not Applicable	
Studies involving **human participants**: Include a statement confirming that **informed consent** was obtained from all subjects and that the experiments conformed to the principles set out in the WMA Declaration of Helsinki and the Department of Health and Human Services Belmont Report.	Not Applicable	
Studies involving **human participants:** For publication of **patient photos**, include a statement confirming that consent to publish was obtained.	Not Applicable	
Studies involving experimental **animals**: State details of **authority granting ethics approval** (IRB or equivalent committee(s), provide reference number for approval. Include a statement of compliance with ethical regulations.	Not Applicable	
Studies involving **specimen and field samples:** State if relevant **permits** obtained, provide details of authority approving study; if none were required, explain why.	Not Applicable	

Dual Use Research of Concern (DURC)	Information included in the manuscript?	In which section is the information available? (Reagents and Tools Table, Materials and Methods, Figures, Data Availability Section)
Could your study fall under dual use research restrictions? Please check biosecurity documents and list of **select agents and toxins** (CDC): https://www.selectagents.gov/sat/list.htm	Not Applicable	
If you used a select agent, is the security level of the lab appropriate and reported in the manuscript?	Not Applicable	
If a study is subject to dual use research of concern regulations, is the name of the **authority granting approval and reference number** for the regulatory approval provided in the manuscript?	Yes	

Reporting

The MDAR framework recommends adoption of discipline-specific guidelines, established and endorsed through community initiatives. Journals have their own policy about requiring specific guidelines and recommendations to complement MDAR.

Adherence to community standards	Information included in the manuscript?	In which section is the information available? (Reagents and Tools Table, Materials and Methods, Figures, Data Availability Section)
State if relevant guidelines or checklists (e.g., **ICMJE, MIBBI, ARRIVE, PRISMA**) have been followed or provided.	Not Applicable	
For **tumor marker prognostic studies,** we recommend that you follow the **REMARK** reporting guidelines (see link list at top right). See author guidelines, under 'Reporting Guidelines'. Please confirm you have followed these guidelines.	Not Applicable	
For **phase II and III randomized controlled trials**, please refer to the **CONSORT** flow diagram (see link list at top right) and submit the CONSORT checklist (see link list at top right) with your submission. See author guidelines, under 'Reporting Guidelines'. Please confirm you have submitted this list.	Not Applicable	

Data Availability

Data availability	Information included in the manuscript?	In which section is the information available? (Reagents and Tools Table, Materials and Methods, Figures, Data Availability Section)
Have **primary datasets** been deposited according to the journal's guidelines (see 'Data Deposition' section) and the respective accession numbers provided in the Data Availability Section?	Yes	Materials and Methods; Figure legends
Were **human clinical and genomic datasets** deposited in a public access-controlled repository in accordance to ethical obligations to the patients and to the applicable consent agreement?	Not Applicable	
Are **computational models** that are central and integral to a study available without restrictions in a machine-readable form? Were the relevant accession numbers or links provided?	Yes	Materials and Methods,C31 Figure legends
If publicly available data were reused, provide the respective **data citations in the reference list**.	Yes	References and Materials and Methods
